# Antimicrobial Activity of Anionic Bis(*N*-Heterocyclic Carbene) Silver Complexes

**DOI:** 10.3390/molecules29194608

**Published:** 2024-09-27

**Authors:** Carlos J. Carrasco, Francisco Montilla, Eduardo Villalobo, Manuel Angulo, Eleuterio Álvarez, Agustín Galindo

**Affiliations:** 1 Departamento de Química Inorgánica, Facultad de Química, Universidad de Sevilla, 41012 Sevilla, Spain; montilla@us.es; 2 Departamento de Microbiología, Facultad de Biología, Universidad de Sevilla, 41012 Sevilla, Spain; evpolo@us.es; 3 Servicio de Resonancia Magnética Nuclear, CITIUS, Universidad de Sevilla, 41012 Sevilla, Spain; mangulo@us.es; 4 Instituto de Investigaciones Químicas, CSIC-Universidad de Sevilla, 41092 Sevilla, Spain; ealvarez@iiq.csic.es

**Keywords:** antimicrobial, silver, *N*-heterocyclic carbene, nuclear magnetic resonance, density functional theory, X-ray crystallography

## Abstract

The antimicrobial properties of a series of anionic bis(carbene) silver complexes Na_3_[Ag(NHC^R^)_2_] were investigated (**2a**–**2g** and **2c′**, where NHC^R^ is a 2,2′-(imidazol-2-ylidene)dicarboxylate-type *N*-heterocyclic carbene). The complexes were synthesized by the interaction of imidazolium dicarboxylate compounds with silver oxide in the presence of aqueous sodium hydroxide. Complexes **2f**,**g** were characterized analytically and spectroscopically, and the ligand precursor **1f** and complexes **2c** and **2g** were structurally identified by X-ray diffraction methods. The anions of **2c** and **2g**, [Ag(NHC^R^)_2_]^3−^, showed a typical linear disposition of C_carbene_-Ag-C_carbene_ atoms and an uncommonly eclipsed conformation of carbene ligands. The antimicrobial properties of complexes **2a**–**g**, which contains chiral (**2b**–**2e** and **2c′**) and non-chiral derivatives (**2a**,**f**,**g**), were evaluated against Gram-negative bacteria, *Escherichia coli* and *Pseudomonas aeruginosa*, and a Gram-positive bacterium, *Staphylococcus aureus*. From the observed values of the minimal inhibitory concentration and minimal bactericidal concentration, complexes **2a** and **2b** showed the best antimicrobial activity against all strains. An interesting chirality–antimicrobial relationship was found, and eutomer **2c′** showed better activity than its enantiomer **2c** against the three bacteria. Furthermore, these complexes were investigated experimentally and theoretically by ^109^Ag nuclear magnetic resonance, and the electronic and steric characteristics of the dianionic carbene ligands were also examined.

## 1. Introduction

*N*-heterocyclic carbenes (NHCs) were isolated and characterized several decades ago, but they still attract great interest due to the huge number of applications they exhibit in a variety of areas [1,2,3]. Their strong coordination to transition metals provides a stable metal–NHC framework that can be used conveniently in chemical, material, and biochemical sciences [4,5]. Recent advances in this area have appeared in basic research, for example, the isolation of a crystalline doubly oxidized carbene [6], and in applied research, the antiviral activity of NHC–silver complexes against SARS-CoV-2 [7]. Regarding the latter, the application of transition metal NHCs in medicinal chemistry is an area of great relevance, where gold and silver complexes have demonstrated antimicrobial and anticancer properties [8,9,10,11,12,13,14,15]. In this area, the development of biologically compatible NHC ligands, for example, chiral [16] and potentially water-soluble NHC ligands [17,18], is of particular importance.

**Scheme 1:**
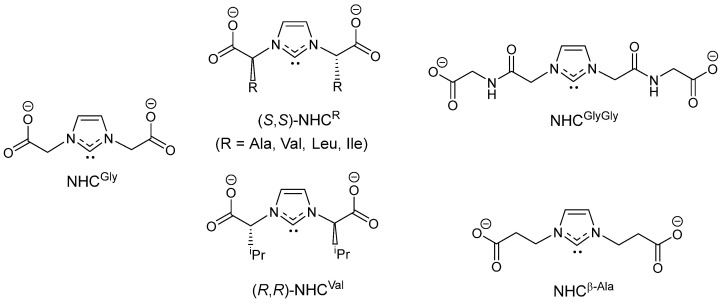
2,2′-(imidazol-2-ylidene)dicarboxylate-type *N*-heterocyclic carbene ligands, NHC^R^ (R = amino acid from which they were prepared), coordinated to silver in complexes **2a**–**g**.

Stereochemistry plays a crucial role in antimicrobial performance, since biological systems usually prefer a specific enantiomer. For example, chirality–activity relationships were described for amino acid-based ionic liquids and poly(ionic liquid) membranes, where those containing *D*-amino acid groups exhibited higher antibacterial activities against *Escherichia coli* and *Staphylococcus aureus* compared to those containing *L*-enantiomeric amino acids [19]. A similar correlation between chirality and antimicrobial activity was described in silver imidazolium–dicarboxylate, [Ag(L^R^)]_n_ [20], and NHC–silver complexes, [Ag(NHC^Mes,R^)]_n_ [21], where compounds {Ag[(*R*,*R*)-L^R^]}_n_ and {Ag[(*R*,*R*)-NHC^Mes,R^]}_n_ showed better antimicrobial properties than their respective (*S*,*S*)-enantiomers. Very recently, the best results regarding the activity of NHC–silver complexes as antimicrobial agents were reviewed [22,23], and surprisingly, the only chiral examples were our complexes [Ag(NHC^Mes,R^)]_n_ [21]. For this reason, and following our interest in the study of transition metal complexes containing ligands derived from amino acids [24,25,26,27,28,29] and their applications as antimicrobial agents [20,21], we decided to study the antimicrobial activity of complexes Na_3_[Ag(NHC^R^)_2_], **2a**–**g** (NHC^R^ is a 2,2′ (imidazol-2-ylidene)dicarboxylate-type *N*-heterocyclic carbene, Scheme 1), against Gram-negative bacteria, *Escherichia coli* and *Pseudomonas aeruginosa*, and a Gram-positive bacterium, *Staphylococcus aureus*. The synthesis and anticancer activity of **2a**–**e** were recently described, and different anticancer behaviors of enantiomerically related complexes were also observed [30]. This set of complexes was extended here with two new examples Na_3_[Ag(NHC^R^)_2_] containing NHC ligands derived from glycine dipeptide (R = CH_2_CONHCH_2_COO^−^, **2f**) and the β-alanine amino acid (R = CH_2_CH_2_COO^−^, **2g**) (Scheme 1). The correlation of chirality–biocidal activity was confirmed for the complex derived from *D*-valine, Na_3_[Ag{(*R*,*R*)-NHC^Val^}_2_], **2c′**, which shows better antimicrobial performance for all strains than its enantiomer derived from *L*-valine Na_3_[Ag{(*S*,*S*)-NHC^Val^}_2_], **2c**. Furthermore, the ^109^Ag NMR chemical shifts in **2a**–**2g** were experimentally determined and theoretically calculated, and a reasonably good correlation between these parameters was found. Finally, the electronic (Tolman’s electronic parameter, TEP) and steric (percent buried volume, %*V*_bur_) properties of the dianionic carbene NHC^R^ ligands, shown in Scheme 1, were also determined.

## 2. Results and Discussion

### 2.1. Synthesis, Characterization, and Theoretical Analysis of Complexes ***2a**–**g***

**Scheme 2:**
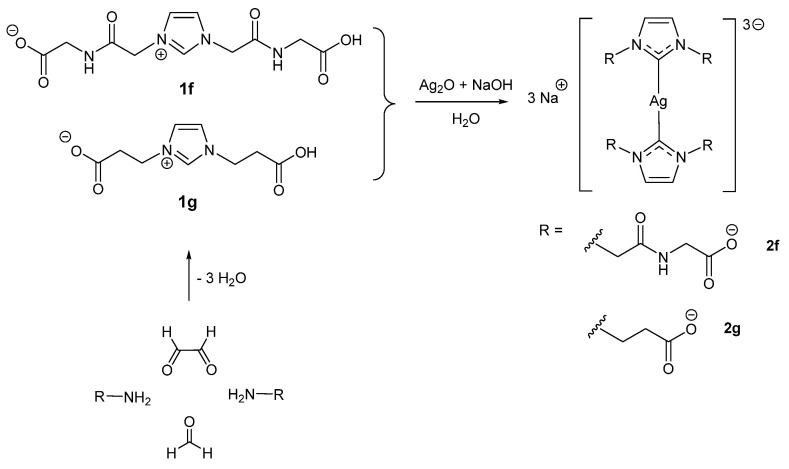
Synthesis of complexes **2f**–**g**.

Complexes Na_3_[Ag(NHC^R^)_2_] (R = Gly, **2a**; Ala, **2b**; Val, **2c**; Leu, **2d**; and Ile, **2e**) were prepared by reaction of imidazolium-dicarboxylate compounds HL^R^, **1a**–**e**, with Ag_2_O in the presence of aqueous sodium hydroxide [30]. The new complexes Na_3_[Ag(NHC^R^)_2_], **2f**–**g**, were synthesized similarly but starting from compounds HL^GlyGly^, **1f**, and HL^β−Ala^, **1g** (Scheme 2). Compounds **1f**–**g** were obtained in a straightforward way by the Debus–Radziszewski reaction using glyoxal, formaldehyde, and the dipeptide glycylglycine or the β-alanine amino acid, respectively (Scheme 2) [31,32,33]. The spectroscopic properties of **1f**–**g** are well matched to those previously described (see Section 3 and Figures S1 and S2) [34,35]. Complexes Na_3_[Ag(NHC^R^)_2_], **2f**–**g**, were obtained as colorless crystals or solids in good yields and were soluble in water, sparingly soluble in methanol, but insoluble in organic solvents. Their IR spectra were characterized by an intense and broad band centered around 1600 cm^−1^, which was assigned to the antisymmetric COO vibrations of the carboxylate groups. This absorption appeared at a lower wavenumber than those of the parent compounds **1f**–**g** (around 1660 cm^−1^, Figure S1), in agreement with a major delocalization of the carboxylate group. Complexes **2f**–**g** showed in the ^1^H and ^13^C{^1^H} NMR spectra the typical pattern due to the NHC ring with signals at ca. 7.3 and 122 ppm for the equivalent CH groups at 4 and 5 ring positions, respectively. In the ^1^H NMR spectrum, the CH_2_ groups of **2f** appear as singlets at 3.78 and 5.11 ppm, while those corresponding to **2g** are a multiplet and a triplet at 2.70 and 4.37 ppm, respectively. In the ^13^C{^1^H} NMR spectra, singlets were observed at around 176 ppm due to carboxylate carbon atoms, in agreement with related NHC–carboxylate silver complexes [29]. Carbene ^13^C resonances, which are often difficult to observe [36], were not detected here. These assignments for the ^1^H and ^13^C NMR signals were confirmed by 2D ^1^H-^13^C correlation NMR spectra.

Precursor compounds **1f**–**g** and their silver complexes **2f**–**g** were analyzed using density functional theory (DFT). Geometry optimizations were performed with the actual compounds, and the resulting optimized structures are shown in Figures S3 and S4. Concerning the anions [Ag(NHC^R^)_2_]^3−^ of complexes **2f**–**g** (Figure S4), the selected combination of the method and basis sets, B3LYP/LANL2DZ/6-311G*, provides a good structural description of these complexes according to the comparison of the calculated and experimental structural parameters of complex **2g** (see the discussion below and Table S1). The conformation of the NHC^R^ ligands in the optimized complexes is not eclipsed but alternated with an angle between the NHC planes of 51.7° (**2f**) and 48.3° (**2g**). The eclipsed disposition of NHC ligands observed in the solid state for **2g** (see discussion below) is due to the interactions of carboxylate groups with sodium cations. In fact, optimization of the actual **2g**, namely Na_3_[Ag(NHC^β−Ala^)_2_], gives an optimized structure in which the NHC ligands are eclipsed (torsion angle of around 12°, see Figure S4). For optimized anions [Ag(NHC^R^)_2_]^3−^ of **2f**–**g**, the chemical shifts of ^1^H- and ^13^C NMR were calculated and excellent correlations with the experimental values were found (Figures S5 and S6). This fact supports the existence of these derivatives as bis(NHC) silver species in solution and additionally confirms the ^1^H and ^13^C NMR assignments.

### 2.2. Structural Characterization of ***1f***, ***2c***, and ***2g*** in the Solid State

**Figure 1:**
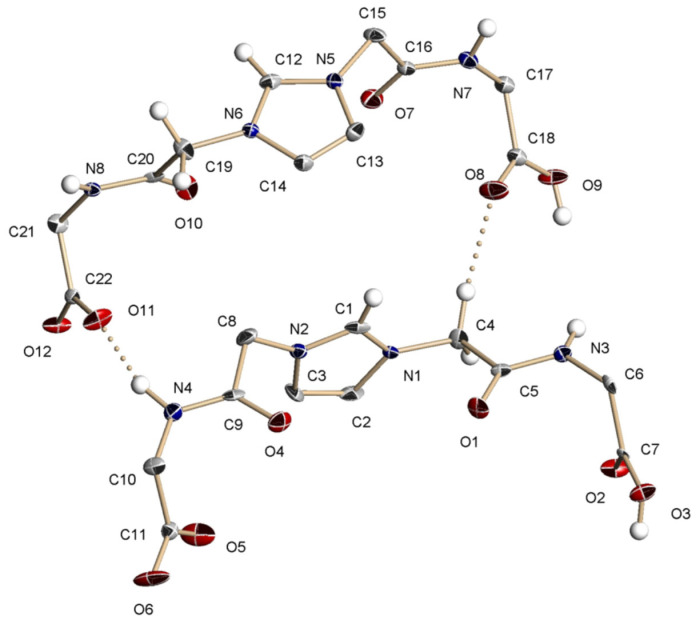
Asymmetric unit of compound **1f**.

During the crystallization of **1f**, we consistently obtained very thin needles or plates of poor quality, leading to weak reflections in its X-ray analysis. This situation was further accentuated by the absence of heavy elements in this compound. This fact is the reason for the somewhat high final *R* value (see Section 3 and Supporting Information), despite having analyzed several crystalline samples obtained in various crystallization processes by X-ray. However, the structure was satisfactorily solved and the structural integrity of **1f** was clearly described. It crystallizes in the triclinic system within the centrosymmetric space group $P\overset{-}{1}$. Selected structural data are collected in Table S2. The asymmetric unit contains two independent molecules of zwitterionic **1f**, linked by intermolecular hydrogen bonds (Figure 1). These molecules also form additional intermolecular hydrogen bonds with symmetric molecules, which control the crystal packing. Structurally, **1f** consists of discrete 2D layers extending along the *ab* plane, with these layers stacked perpendicularly to the *c* axis along the crystal (Figure S7).

**Figure 2:**
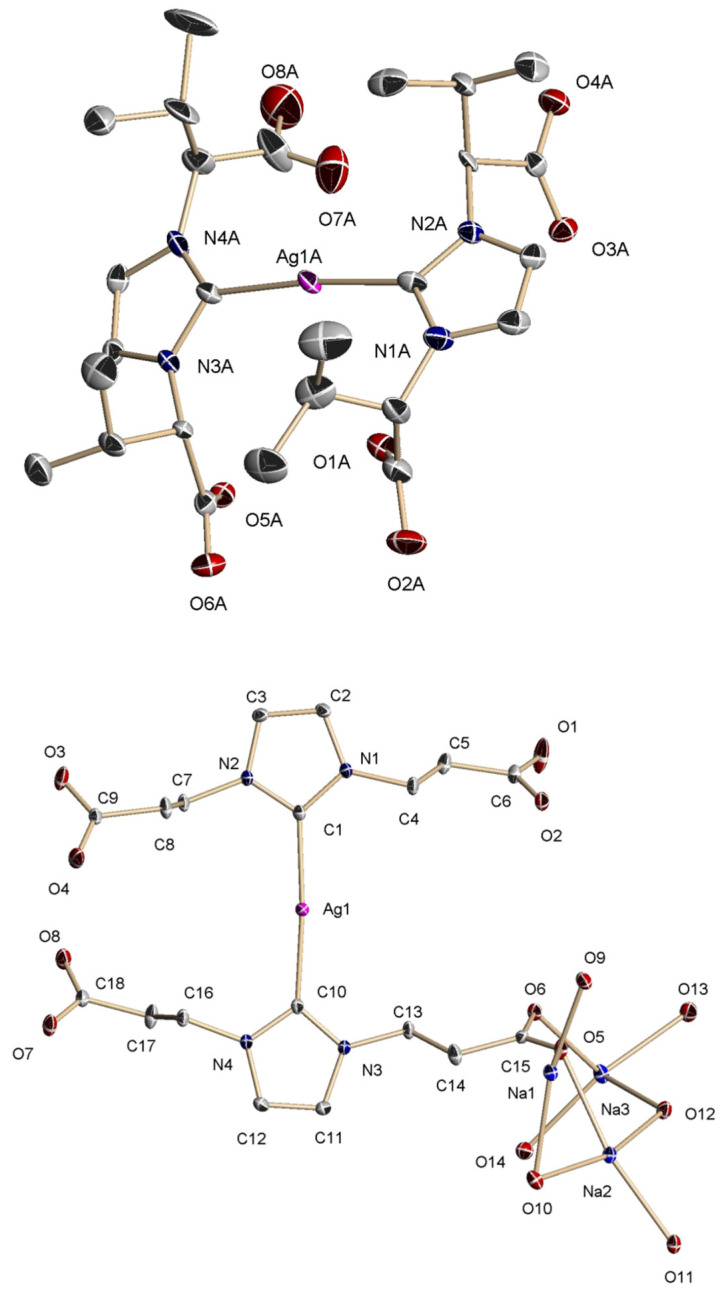
Anion of **2c** (**up**). Asymmetric unit of complex **2g** (**bottom**). Hydrogen atoms and molecules of water crystallization have been omitted for the sake of clarity.

Successful crystallization of Na_3_[Ag(NHC^Val^)_2_], **2c**, and Na_3_[Ag(NHC^β−Ala^)_2_], **2g**, occurred by cooling a concentrated aqueous solution of the complex at 0 °C. Both complexes crystallize in the triclinic system in the space groups *P*1 and $P\overset{-}{1}$, respectively. One of the four symmetrically independent but equivalent anions of **2c** and the asymmetric unit of **2g** are shown in Figure 2, while selected structural data are collected in Tables S9 and S10, respectively. The anionic part of **2c** and **2g** consists of the silver(I) ion and two NHC^R^ ligands that showed a typical linear disposition (C_carbene_-Ag-C_carbene_ angles of 174.5(4) and 175.39(9)°, respectively). Coordinated NHC^R^ ligands appeared in an eclipsed conformation with a maximum torsion angle of around 11°. The Ag-C bond distances were slightly higher in **2c** for any of the four anions in the asymmetric unit (for example, 2.123(11) and 2.148(12) Å) than those found in **2g** (2.081(2) and 2.084(2) Å). In both complexes, the C-O carboxylate distances are typical of delocalized carboxylate groups (for example, the range 1.232(3)-1.263(3) Å for **2g**). These structural data agree well with those of the complex Na_3_[Ag(NHC^Gly^)_2_], **2a** [20], and with other related silver mononuclear bis(carbene) complexes [37,38,39,40]. The asymmetric unit of **2c** is composed of four [Ag(NHC^Val^)_2_]^3−^ anions and twelve hydrated sodium cations, [Na_4_(H_2_O)_42_]^4+^. These ions were arranged along the *ab* plane in a 2D polymeric disposition with some carboxylate–sodium interactions (the shortest distance is Na6-O1B, 2.341(15) Å) and a complex hydrogen bonding network between carboxylate groups and water molecules of hydration (Figure S8). In **2g**, three of the four carboxylate groups were involved in interactions with sodium cations (from the shortest Na1-O5 distance of 2.328(2) Å to the longest distance of 2.757(3) Å for Na3-O1(#6)). Furthermore, there is a complex hydrogen bonding network between hydrated sodium cations (as [Na_3_(H_2_O)_6_]^3+^ units), carboxylate groups of carbene ligands and water molecules of hydration. The 3D crystal packing was controlled by these hydrogen bonds and showed along the *a* axis a 1D assembly of hydrated sodium ions that were coordinated by carboxylate groups of [Ag(NHC^β−Ala^)_2_]^3−^ arranged along the *c* axis (Figure S9). The planes defined by the eclipsed carbene ligands of each [Ag(NHC^β−Ala^)_2_]^3−^ entity corresponding to two neighbor arrangements along the *c* axis were perfectly parallel with a distance between the planes of ca. 3.49 Å.

### 2.3. Antimicrobial Studies

The antimicrobial activity of complexes **2** against Gram-negative bacteria, *E. coli* and *P. aeruginosa*, and the Gram-positive bacterium, *S. aureus*, was evaluated by determining the minimal inhibitory growth concentration (MIC) and the minimal bactericidal concentration (MBC). The results, presented in Table 1 as mM concentrations (the data in µg/mL are shown in Table S7), suggested that the dissociation of the Ag^+^ ion from complexes **2** cannot be solely responsible for antimicrobial activity. This is evident, as some MIC and MBC values are lower than those of AgNO_3_, a widely used antimicrobial agent against these bacteria. Since the precursor compounds HL^R^ did not show any biocidal activity, the biocidal effect was attributed to the silver–NHC^R^ complex. The values in Table 1 can be compared with other NHC–silver complexes from recent reviews that summarized the best biocidal results for this type of complexes [22,23]. A significant difference from other silver complexes we studied, which showed activity only against Gram-negative bacteria [20,21], is the antimicrobial effect observed here against the Gram-positive bacterium *S. aureus*. The MIC values for complexes **2** against *S. aureus* were in the range of 0.13–0.27 mM, with complex **2b** exhibiting the highest activity (0.134 mM). Although this value is higher than the best inhibition reported for a NHC–silver complex [22,41], it correlates well with the values reported for related bis(NHC)–silver complexes [22,42]. With respect to Gram-negative bacteria, the MIC values of complexes **2** against *P. aeruginosa* were in the range of 0.16–0.30 mM, with complexes **2a** and **2b** showing the best biocidal activity (0.167 mM). This was higher than the value reported for the chiral NHC–silver complex {Ag[(*R*)-NHC^Mes,Me^]}_n_, 39 μM [21], or that for a silver complex with a bidentate biphenyl NHC ligand functionalized with picolyl and benzyl substituents [43]. The presence of the mesityl substituent in the former and the biphenyl bridge and the benzimidazolium ring in the latter provided higher lipophilicity to these compounds compared to **2b**, justifying their increased antimicrobial activity. For the *E. coli* strain, complexes **2** showed moderate antimicrobial activity (MIC values within the range of 0.20–0.27 mM). Complex **2a** showed the best MIC result, although it is higher than the best reported values of 4–8 μg/mL for other NHC–silver complexes [41,44,45,46,47].

Recently, we described a relationship between the antimicrobial activity of related NHC–silver complexes {Ag[NHC^Mes,R^]}_n_ and the steric properties of the alkyl R group for *E. coli* and *P. aeruginosa* [21]. However, this trend was not evident for complexes **2**, despite the fact that the best results in Table 1 were found for **2a** and **2b**, which possess the less bulky demanding R groups. The synthesis of a derivative with glycylglycine functionality was planned with the aim of obtaining a better biocompatibility, but unfortunately, complex **2f**, and similarly **2g**, displayed higher MIC values than the simplest counterpart of the series, **2a**. Interestingly, the comparison of MIC and MBC values for all strains of the enantiomeric complexes **2c** and **2c′** confirmed the relationship between chirality and antimicrobial activity, as we previously observed in related silver systems. Complex Na_3_[Ag{(*R*,*R*)-NHC^Val^}_2_], **2c′**, which was derived from *D*-valine, is the eutomer of all strains and showed better biocidal activity than its enantiomer Na_3_[Ag{(*S*,*S*)-NHC^Val^}_2_], **2c**, derived from *L*-valine. This result confirmed the connection between chirality and biological activity, suggesting a possible generalization of this chirality–antimicrobial trend.

Complexes **2** showed interesting activity properties to be considered as antimicrobial biocidal agents. For this reason, the possible biocompatibility of complexes **2** was analyzed through a qualitative study of its hemolytic activity. For this purpose, complex **2a**, which showed the highest biocidal activity, was selected, and its hemolytic activity was investigated in a culture medium containing sheep blood on agar (see Section 3). The qualitative results obtained are shown in Figure S15. Complex **2a** did not show appreciable hemolytic activity after 18 h at the concentrations studied (10 mM, two orders of magnitude higher than the observed MIC values). This contrasts with the hemolysis observed for sodium dodecyl sulfate, SDS 1%, and hydrogen peroxide 10%, used as positive controls (Figure S15).

### 2.4. NMR Solution Behavior of Complexes ***2***: Experimental and Computational Determination of ^109^Ag NMR Chemical Shifts

The ^1^H and ^13^C{^1^H} NMR spectra of complexes Na_3_[Ag(NHC^R^)_2_] were previously reported for **2a**–**e** [30] and discussed here in a previous section for **2f**–**g**. In some of these spectra, a set of signals of minor intensity occasionally appeared, suggesting the presence of an isomer of the [Ag(NHC^R^)_2_]^3−^ species. Using variable temperature studies and selective ROESY experiments, no interconversion was observed between the possible isomer and the corresponding complex **2**. This fact rules out the possibility of a rotamer, due to the restricted rotation of the carbene ligands around the Ag-C_carbene_ vector. Furthermore, ^1^H DOSY NMR experiments performed on solutions of complexes **2b** and **2c** in methanol-*d*_4_ at 233 K (Figure S12) showed a higher diffusion coefficient for the minor species, with a diffusion coefficient ratio of 1.2 and 1.3 for **2b** and **2c**, respectively. These results indicated different hydrodynamic radii for **2b** (or **2c**) and the minor compound, thereby eliminating the assignment of this minor species as an isomer of **2b** (or **2c**). Since the observation of multiplet signals corresponding to carbene carbon atoms is difficult in a standard ^13^C{^1^H} NMR spectrum, a band selective constant time ^1^H-^13^C HMBC NMR experiment was performed on compound **2c**, increasing the resolution of the ^13^C dimension using non-uniform sampling (NUS). This experiment allowed the observation of a correlation signal at 135.9 ppm (Figure S13) with triplet multiplicity (^1^*J*_C-D_ ≈ 34 Hz). This is typical of the deuteration of the C^2^-H group of the imidazolium ring and was observed by us in related silver carboxylate complexes {Ag[(*S*,*S*)-L^R^]}_n_ [20]. Therefore, these minor signals in the spectra of **2** were identified as complexes {Ag[(*S*,*S*)-L^R^]}_n_. We previously demonstrated that the interaction of {Ag[(*S*,*S*)-L^R^]}_n_ with NaOH produced complexes **2**, and thus, the presence of small amounts of {Ag[(*S*,*S*)-L^R^]}_n_ may be due to an incomplete reaction. In fact, no increase in the signals due to {Ag[(*S*,*S*)-L^R^]}_n_ was observed over time and, consequently, the formation of this carboxylate species by hydrolysis of carbene complexes was excluded.

**Figure 3:**
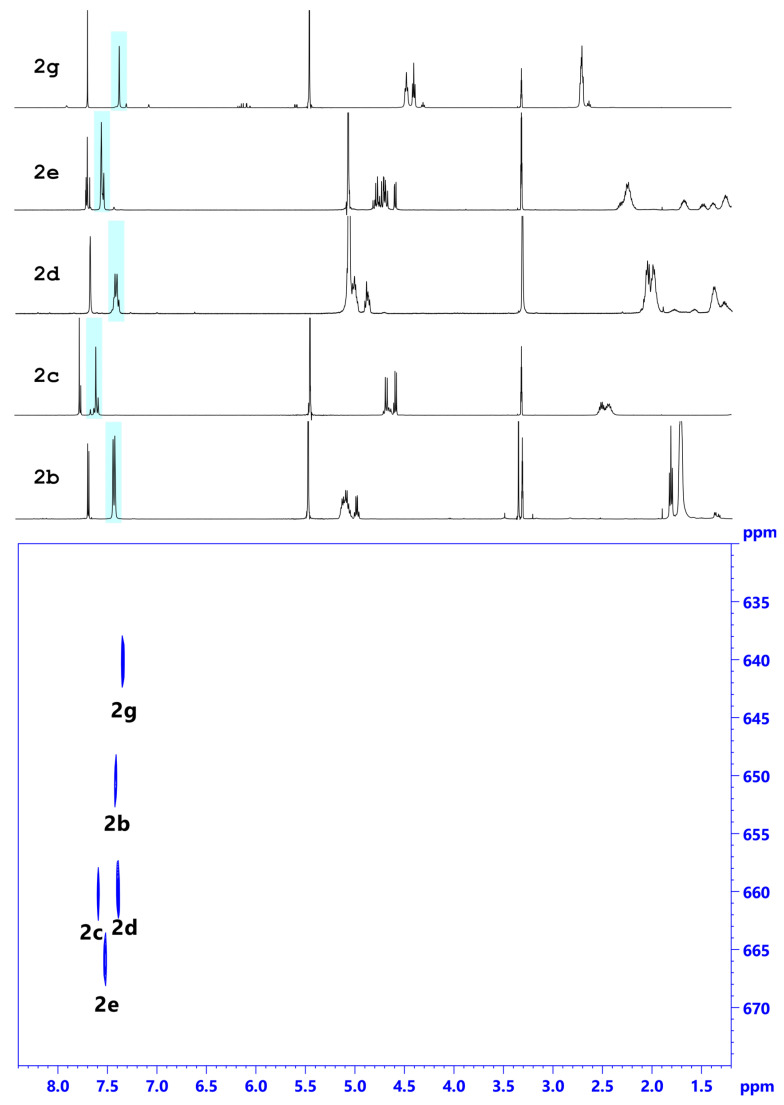
Superposition of the ^1^H-^109^Ag-HMBC NMR spectra of **2b**,**c**,**d**,**e**, and **2g**.

Due to the low sensitivity and long spin-lattice relaxation time, silver NMR is usually observed indirectly via ^1^H detected through bond heteronuclear correlation experiments, using the presence of coupled protons located at several bonds. For these studies, ^109^Ag is the preferred isotope due to its higher receptivity compared to ^107^Ag. Indirect detection of ^109^Ag resonances in complexes **2** was performed using two-dimensional ^1^H-^109^Ag-HMBC spectra. In the spectra of these complexes, except for **2a** and **2f**, ^1^H-^109^Ag cross-peaks were observed (Figure 3) between the ^109^Ag resonance and hydrogen atoms of the equivalent CH groups at the 4 and 5 ring positions (*J* through four bonds). Table 2 collects the ^109^Ag chemical shifts of complexes **2**, which appear in the narrow range of 640–666 ppm. This fact suggests that δ(^109^Ag) is not dependent on the nature of the R substituent in this Na_3_[Ag(NHC^R^)_2_] series.

**Figure 4:**
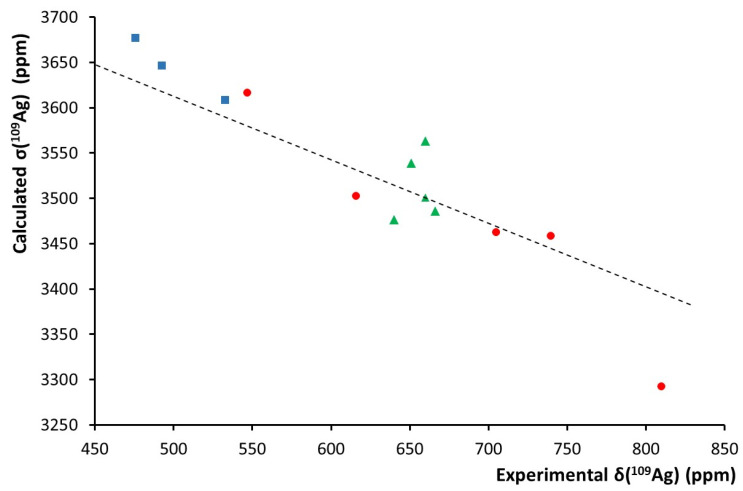
Correlation between experimental δ(^109^Ag) and calculated σ(^109^Ag) for a series of NHC–silver complexes ([Ag(NHC^R^)_2_]^3−^, green triangles; [Ag(SIMes)X], red circles; and [Ag(NHC^R2,Ph2^)(OOCCH_3_)], blue squares).

The experimental determination of the ^109^Ag resonances led us to determine these values theoretically as well. The number of theoretical studies devoted to these calculations is limited and, to our knowledge, there are no reports for NHC–silver derivatives [48]. Alkorta et al. recently reported one of the most interesting studies in which ^109^Ag NMR chemical shifts of trinuclear pyrazolate silver complexes were obtained both experimentally and computationally [49,50]. To select the best computational method to perform these δ(^109^Ag) determinations for silver carbene complexes, a set of eight complexes was selected. These include complexes [Ag(SIMes)X] (X = Me, Mes, N(SiMe_3_)_2_, O^t^Bu and O(2,6-^t^Bu-4-Me-C_6_H_2_); SIMes = 1,3-bis-(2,4,6- trimethylphenyl)-4,5-dihydroimidazol-2-ylidene) recently described by Coperet [51] and complexes [Ag(NHC^R2,Ph2^)(OOCCH_3_)] (R2 = 2 Me; Me, ^i^Pr; and 2 ^i^Pr) reported by Tacke [52]. They were well characterized, most of them by X-ray crystallography, and all with experimental ^109^Ag NMR data. The eight complexes were optimized without symmetry restrictions at the B3LYP/LANL2DZ/6-311G* level of theory and a good concordance was found with the experimental structural data (Table S6). For NMR calculations, the use of LANL2DZ for silver gave poor results for the δ(^109^Ag) values, and instead, the DGDZVP basis set was used [53]. This basis set has been described as a good alternative to the triple zeta basis sets due to its small size [54,55]. The correlation between experimental ^109^Ag chemical shifts and the calculated absolute chemical shielding σ(^109^Ag) (Figure S14) for these NHC complexes, [Ag(SIMes)X] and [Ag(NHC^R2,Ph2^)(OOCCH_3_)], is quite good. The *R*^2^ coefficient of the correlation was 0.946 and the relationship found was *δ_exp_*(^109^Ag) = (4152.9 − *σ_calc_*)/1.0088. This validates the computational method for its application to our derivatives [Ag(NHC^R^)_2_]^3−^. In fact, the inclusion of the experimental and calculated ^109^Ag resonances for complexes **2** in this correlation (see Table 2) is shown in Figure 4. The new data did not significantly reduce the *R*^2^ coefficient, 0.9028, and the relationship with all data is *δ_exp_*(^109^Ag) = (3962.8 − *σ_calc_*)/0.7003. This correlation *R*^2^ result is better than the value obtained by Alkorta (*R*^2^ = 0.71). The weak correlation obtained for pyrazolate complexes was attributed to the low Δδ/range ratio (0.02) [49]. For this reason, we included three different types of silver–NHC complexes that cover a Δδ of 334 ppm and included the complex [Ag(H_2_O)_2_]^+^ as δ = 0 ppm reference. Therefore, a higher Δδ/range ratio of 0.62 was obtained for our series. In any case, the corrected calculated δ(^109^Ag) values show an average deviation of approximately 27 ppm, with **2d** being the most poorly described complex.

### 2.5. Electronic and Steric Properties of NHC^R^ Ligands

**Figure 5:**
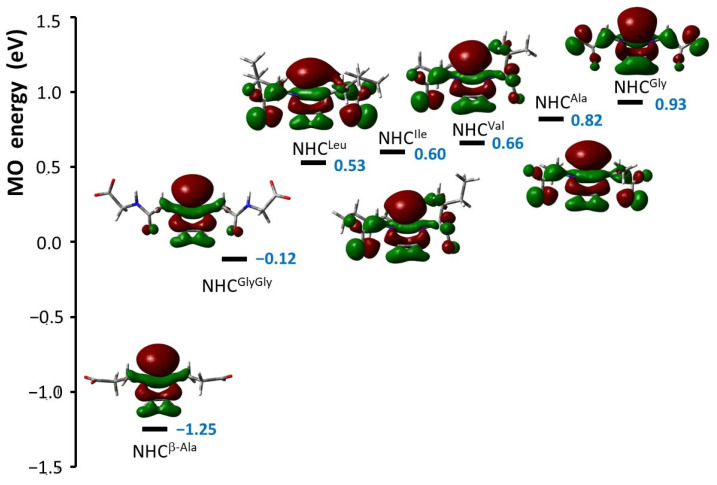
Energies of MOs involved in the σ donation of carbene to silver from NHC^R^ ligands.

The influence of the electron-donating/withdrawing properties of the aryl substituents on the bonding capabilities of the related carbene-monocarboxylate [NHC^Ar,R^]^−^ ligands was previously analyzed by us [21]. Similarly, to determine the electronic and steric properties of the dianionic carbene ligands of complexes **2** (chiral (*S*,*S*)-NHC^R^, for R = Ala, Val, Leu, Ile, and achiral NHC^Gly^, NHC^GlyGly^ and NHC^β−Ala^ ligands, Scheme 1), they were optimized at the B3LYP/6-311G* theoretical level without symmetry restrictions. Analysis of the MOs obtained for NHC^GlyGly^ and NHC^β−Ala^ revealed that the σ lone pair of the carbene carbon atom was found in HOMO-6, while the analogous MO for the NHC^Gly^ and (*S*,*S*)-NHC^R^ counterparts was found in HOMO-4. These MOs and their energies are shown in Figure 5, where the influence of the substituent on the MO energy is clearly observed. An increase in the alkyl chain results in a decrease in the energy of the σ donor MO. Lower values were found in NHC^GlyGly^ and NHC^β−Ala^ carbenes, indicating weaker σ donor capabilities than their homologues. To confirm these results, the Tolman Electronic Parameter [56] (TEP) of NHC^R^ ligands [57] was calculated following the approach proposed by Gusev [58]. The TEP values were obtained through the calculated and scaled ν_CO_ vibration of A_1_ symmetry from the optimization of the complexes [Ni(CO)_3_(NHC^R^)]^2−^ (see Table 3 and Table S4). The calculated TEP values for NHC^R^ fall within the range of 2018–2040 cm^−1^ and are lower than those of the commonly encountered IMes and SIMes carbenes (2050.5 and 2051.2 cm^−1^, respectively). This difference is due to the dianionic nature of these carbenes compared with that of neutral NHCs. In fact, the calculated TEP value of the neutral methyl diester of NHC^Gly^ gave a TEP value of 2057.7 cm^−1^, which is close to those of IMes and SIMes. As noted by Gusev [58], TEP decreases as the size of the *N*-substituent increases, in agreement with the same conclusion obtained from the MO energy (Figure 5). Moreover, there is an interesting correlation between the calculated TEP of NHC^R^ and the MO energy of the σ lone pair orbitals of the carbene carbon atom (see Figure S10), confirming the superior σ donor capacity of the NHC^Gly^ ligand and the weaker σ donation of the NHC^GlyGly^ and NHC^β−Ala^ ligands.

The steric pressure of the NHC^R^ ligands was determined by calculating the percent buried volume, %*V*_bur_, using the SambVca 2.1 software [59,60]. Optimized structures of the complexes [Ni(CO)_3_(NHC^R^)]^2−^ and [Ag(NHC^R^)_2_]^3−^ were used as input, with the distances for the M-C bond fixed to 2 Å. The results are shown in Table 3 and Table S5, where two types of carbene can be distinguished. (*S*,*S*)-NHC^R^ ligands have values close to 30, which are lower, as expected, than those found for IMes and SIMes (36.1 [61]). They exhibit more steric pressure than the non-chiral ligands NHC^Gly^, NHC^GlyGly^, and NHC^β−Ala^, which have a %*V*_bur_ value of around 26, similar to other NHC ligands with methyl substituents bonded to the N atom (26.1 [61]). Selected steric maps for both types of ligands are shown in Figure S11. Furthermore, %*V*_bur_ was determined from the X-ray data (Table S5) for the NHC^Gly^, NHC^Val^, and NHC^β−Ala^ ligands. Higher values were observed in comparison to those obtained from optimized structures. The discrepancy can be attributed to the different relative orientations of carboxylate groups in the gas phase calculation compared to the solid state, where such orientations were controlled by interactions with sodium counterions and the hydrogen-bonding network.

## 3. Materials and Methods

### 3.1. General

All preparations and other operations were carried out under anaerobic conditions. Solvents were properly purified prior to use, following standard procedures. Chemicals were obtained from various commercial sources and used as supplied. Zwitterionic imidazolium dicarboxylate compounds **1f**–**g** [34,35] and complexes **2a**–**e** [30] were prepared according to the literature procedures. Infrared spectra were recorded using the ATR technique on a PerkinElmer FT-IR Spectrum Two (Waltham, MA, USA). NMR spectra were recorded at the Centro de Investigaciones, Tecnología e Innovación (CITIUS) of the University of Sevilla using Avance III spectrometers (Billerica, MA, USA). ^1^H and ^13^C{^1^H} NMR shifts were referenced to residual signals from deuterated solvents. All data are reported in ppm downfield from Si(CH_3_)_4_. Polarimetry was carried out using a JASCO P-2000 digital polarimeter (JASCO Analitica Spain s.l., Madrid, Spain) and the measurements were carried out at room temperature (concentration of ca. 10 mg/mL, Table S3). Elemental analyses (C, H, N) were performed by CITIUS of the University of Sevilla on an Elemental LECO CHNS 93 analyzer (LECO Corporation, St. Joseph, MI, USA). High-resolution mass spectra were obtained on a QExactive Hybrid Quadrupole-Orbitrap mass spectrometer from Thermo Scientific (Waltham, MA, USA) (CITIUS of the University of Sevilla).

### 3.2. Synthesis

(2-(1-(2-((carboxymethyl)amino)-2-oxoethyl)-1H-imidazol-3-ium-3-yl) acetyl) glycinate, HL^GlyGly^ (**1f**). Compounds glycylglycine (10 g, 0.076 mol), glyoxal (4.4 mL, 0.038 mol), and formaldehyde (2.9 mL, 0.038 mol) were mixed in a 100 mL flask, dissolved in Millipore H_2_O (30 mL) and heated at 60 °C overnight. The solution was then left to crystallize in air, yielding a pale brown solid of compound **1f**, which was filtered, washed with cold water and dried under vacuum (4.85 g, 43% yield). IR (ATR, cm^−1^): 3245 (w), 3081 (w), 3037 (w), 2951 (w), 2983 (w), 1663 (vs), 1594 (m), 1564 (s), 1440 (m), 1414 (m), 1376 (m), 1353 (m), 1308 (m), 1264 (m), 1241 (s), 1219 (s), 1188 (vs), 1095 (s), 1034 (vs), 975 (m), 905 (m), 889 (s), 778 (vs), 717 (s), 689 (s), 662 (s), 630 (s), 597 (s), 572 (m), 553 (m), 531 (m), 503 (m), 420 (w). ^1^H NMR (D_2_O, 300 MHz): δ 3.93 (s, 4H, C*H*_2_COO), 5.15 (s, 4H, C*H*_2_Im), 7.56 (d, 2H, *J* = 2 Hz, C*H*, H^4^/H^5^), 8.94 (s, 1H, C*H*, H^2^). ^13^C{^1^H} NMR (D_2_O, 75 MHz): δ 42.4 (s, *C*H_2_COO), 50.9 (s, *C*H_2_Im), 123.6 (s, *C*H, C^4^/C^5^), 138.5 (s, *C*H, C^2^), 167.1 (s, *C*ONH), 174.6 (s, *C*OO). HR-MS (negative mode), found: *m*/*z* = 299.0987, calculated for C_11_H_15_N_4_O_6_ [HL^GlyGly^ + H]^−^, 299.0986. Elemental Anal. Calc. for C_11_H_14_N_4_O_6_ (**1f**): C, 44.30; H, 4.73; N, 18.79. Found: C, 44.44; H, 4.76, N, 18.25%.

3-(1-(2-carboxyethyl)-1H-imidazol-3-ium-3-yl)propanoate), HL^β−Ala^ (**1g**). Compounds β-alanine, (10 g, 0.11 mol), glyoxal (6.5 mL, 0.056 mol) and formaldehyde (4.3 mL, 0.056 mol) were mixed in a 100 mL flask, dissolved in Millipore H_2_O (20 mL) and heated at 70 °C for 2.5 h. The solution was then left to crystallize in air, yielding a pale brown solid of compound **1g**, which was filtered, washed with cold water and dried under vacuum (9.9 g, 83% yield). IR (ATR, cm^−1^): 3251 (w), 3168 (w), 3151 (m), 3108 (m), 3087 (m), 3038 (m), 2954 (m), 2885 (w), 1664 (s), 1635 (s), 1568 (m), 1553 (s), 1442 (m), 1413 (m), 1370 (m), 1318 (w), 1291 (w), 1242 (m), 1220 (w), 1188 (m), 1150 (vs), 1132 (m), 1064 (m), 1033 (m), 1007 (w), 978 (m), 944 (m), 906 (m), 893 (m), 871 (m), 836 (vs), 778 (s), 755 (vs), 702 (s), 666 (s), 641 (vs), 632 (vs), 599 (m), 569 (m), 551 (m), 525 (vs), 409 (m). ^1^H NMR (D_2_O, 300 MHz): δ 2.82 (t, 4H, *J* = 7Hz, C*H*_2_COO), 4.39 (t, 4H, *J* = 7 Hz), C*H*_2_Im), 7.46 (d, 2H, *J* = 2 Hz), C*H*, H^4^/H^5^), 8.77 (s, 1H, C*H*, H^2^). ^13^C{^1^H} NMR (D_2_O, 75 MHz): δ 35.7 (s, *C*H_2_COO), 45.9 (s, *C*H_2_Im), 122.4 (s, *C*H, C^4^/C^5^), 136.1 (s, *C*H, C^2^), 176.0 (s, *C*OO). HR-MS (negative mode), found: *m*/*z* = 211.0723, calculated for C_9_H_11_N_2_O_4_ [HL^β−Ala^]^−^, 211.0724. Elemental Anal. Calc. for C_9_H_12_N_2_O_4_ (**1g**): C, 50.94; H, 5.70; N, 13.20. Found: C, 50.88; H, 5.74, N, 12.87%.

Sodium bis(1,3-bis(2-((carboxylatomethyl)amino)-2-oxoethyl)-imidazol-2-ylidene)argentate(3-), Na_3_[Ag(NHC^GlyGly^)_2_] (**2f**). Compounds HL^GlyGly^, **1f**, (0.298 g, 1.00 mmol) and Ag_2_O (0.058 g, 0.25 mmol) were mixed in a Schlenk flask and dissolved in deoxygenated H_2_O (5 mL) under a nitrogen atmosphere. NaOH (0.060 g, 1.5 mmol) was then added, and the mixture was stirred for 16 h at room temperature in the dark. Afterward, the mixture was centrifuged and filtered, and the filtrate was concentrated to one-quarter of the volume using an intermediate trap. EtOH was added as a cosolvent until precipitation of a white solid was observed. The solution was then cooled to 0 °C. Uncolored crystals of compound **2f** were obtained (0.180 g, 47% yield). IR (ATR, cm^−1^): 3294 (m), 3096 (w), 1662 (s), 1601 (vs), 1558 (s), 1454 (m), 1387 (vs), 1328 (m), 1270 (m), 1251 (m), 1183 (m), 1031 (m), 957 (w), 914 (m), 818 (w), 769 (m), 757 (m), 675 (s), 633 (m), 559 (s), 536 (s), 515 (s), 413 (w). ^1^H NMR (D_2_O, 300 MHz): δ 3.78 (s, 4H, C*H*_2_COO), 5.11 (s, 4H, C*H*_2_CONH), 7.54 (d, 2H, *J* = 2 Hz, C*H*, H^4^/H^5^). ^13^C{^1^H} NMR (D_2_O, 75 MHz): δ 43.4 (s, *C*H_2_COO), 51.0 (s, *C*H_2_CONH), 123.5 (s, *C*H, C^4^/C^5^), 166.8 (s, *C*ONH), 176.3 (s, *C*OO). Elemental Anal. Calc. for C_22_H_26_N_8_O_13_AgNa_3_ (**2f**·H_2_O): C, 33.56; H, 3.33; N, 14.23. Found: C, 33.65; H, 3.38, N, 13.83%.

Sodium bis(1,3-bis(2-carboxylatoethyl)-imidazol-2-ylidene)argentate(3-), Na_3_[Ag(NHC^β−Ala^)_2_] (**2g**). Compounds HL^β−Ala^, **1g**, (0.212 g, 1.00 mmol) and Ag_2_O (0.058 g, 0.25 mmol) were mixed in a Schlenk flask and dissolved in deoxygenated H_2_O (5 mL) under a nitrogen atmosphere. NaOH (0.060 g, 1.5 mmol) was then added and a dark brown solid was observed. The mixture was stirred for 16 h at room temperature in the dark. Afterward, the mixture was centrifuged and filtered, and the filtrate was concentrated to one-quarter of the volume using an intermediate trap. EtOH was then added as a cosolvent until precipitation of a white solid was observed. The solution was then cooled to 0 °C and uncolored crystals of compound **2g** were obtained (0.200 g, 67% yield). IR (ATR, cm^−1^): 3137 (m), 3088 (m), 2937 (m), 1567(vs), 1448 (m), 1400 (vs), 1338 (m), 1307 (m), 1284 (m), 1252 (m), 1231 (m), 1182 (w), 1158 (m), 1110 (w), 1052 (m), 1024 (w), 980 (w), 936 (m), 865 (m), 838 (w), 789 (m), 747 (s), 686 (s), 665 (s), 643 (m), 618 (m), 542 (m), 475 (m), 441 (s), 414 (m). ^1^H NMR (D_2_O, 300 MHz): δ 2.70 (m, 8H, C*H*_2_COO), 4.37 (t, 8H, C*H*_2_NHC), 7.17 (s, 4H, C*H*, H^4^/H^5^). ^13^C{^1^H} NMR (D_2_O, 75 MHz): δ 39.8 (s, *C*H_2_COO), 48.8 (s, *C*H_2_NHC), 121.2 (s, *C*H, C^4^/C^5^), 179.2 (s, *C*OO). Elemental Anal. Calc. for C_18_H_26_N_4_O_11_AgNa_3_ (**2g**·3H_2_O): C, 33.20; H, 4.02; N, 8.60. Found: C, 33.26; H, 4.34, N, 8.33%.

### 3.3. Antimicrobial Studies

The bacteria used in this work were purchased from the Spanish type of culture collection (CECT), and included *Escherichia coli* (CECT 434), *Pseudomonas aeruginosa* (CECT 108) and *Staphylococcus aureus* (CECT 5190). The growth medium (YPD: 10 g/L yeast extract, 20 g/L bactopeptone, 20 g/L glucose, and 2% agar if needed) was sterilized by autoclaving (115 °C for 20 min) before use. Bacterial strains were streaked onto YPD agar plates for colony isolation, from which a single, well-isolated colony was selected to prepare the pre-inocula in YPD. Pre-inocula were grown at 37 °C for 18 h with agitation (180 rpm) on an orbital shaker. To determine the minimum inhibitory concentration (MIC), 30 μL of the pre-inocula (diluted at 1/50) were inoculated into tubes containing either 3 mL of YPD (positive control) or 3 mL of YPD with various concentrations of the complex **2** under study. Stock solutions of the different complexes **2** were prepared in water at 10 mM. Each assay included a negative control, a tube containing 3 mL of YPD without bacterial inoculation. Subsequently, the tubes were incubated at 37 °C with agitation at 180 rpm. After 18 h of incubation, the tubes were visually inspected for the absence of turbidity or its presence, the latter indicating bacterial growth. The MIC was the lowest concentration of complex **2** that inhibited growth (no turbidity in the tube, similar to the negative control tube). For the minimum bactericidal concentration (MBC), 30 μL of the media from the MIC tubes were re-inoculated in tubes containing 3 mL of YPD alone. These assays also included negative control tubes. All tubes were incubated again at 37 °C on an orbital shaker at 180 rpm for 18 h and visually inspected for turbidity. The MBC was the lowest concentration of complex **2** that did not result in turbidity, indicating bacterial death due to complex **2** from the previous MIC tube. As a well-known growth inhibitory and bactericidal control, AgNO_3_ was used. AgNO_3_ was prepared as a 10 mM solution in water and subjected to the same test conditions as described for complex **2**. Both complex **2** and AgNO_3_ were freshly prepared and independently tested three times with the three bacterial strains.

### 3.4. Hemolysis Assays

An on-agar diffusion assay was conducted using a culture medium commonly employed to test for bacterial-induced hemolysis. The medium contained 15 g/L casein peptone, 5 g/L soy peptone, 5 g/L NaCl, 5% sheep blood, and 15 g/L agar, pH 7.3. Five microliter volumes of control and experimental samples, complex **2a**, were applied onto the blood agar medium (see additional details in Figure S15). The plates were then incubated at 37 °C and photographed at various time intervals to monitor hemolysis. A positive hemolytic reaction was identified by the release of hemoglobin from erythrocytes, indicated by the appearance of red coloration due to oxyhemoglobin (positive control of sodium dodecyl sulfate, SDS 1%) and/or white coloration due to complete denaturation of hemoglobin (positive control of H_2_O_2_ 10%).

### 3.5. NMR Details

All ^1^H-^109^Ag HMBC spectra were recorded on a Bruker Avance NEO 500 MHz spectrometer equipped with a broad band observe (BBFO) probe. The standard gradient selected Bruker HMBC pulse sequence (hmbcgplpndprqf) was used, including a low power presaturation to reduce the intensity of the residual water signal, to improve the dynamic range of the receiver. The delay corresponding to the evolution of the long-range coupling constant was optimized for 1.6 Hz, in agreement with data previously described for similar compounds [62]. The experiments were recorded in methanol-*d*_4_ at 233 K (for **2b**, **2c,** and **2g**) or 278 K (for **2d** and **2e**). Diffusion experiments (DOSY) were acquired in methanol-*d_4_* at 233 K, using the stimulated echo sequence with bipolar gradient pulses and longitudinal eddy current delay (ledbpgp2s) using a linear ramp of 16 values of the pulsed field gradient, from 2 to 95% of the maximum gradient strength. The band-selective constant-time ^1^H-^13^C HMBC experiment was acquired at 298 K using the standard Bruker (Billerica, MA, USA) pulse sequence (shmbcctetgpl2nd) with a spectral width of 36.5 ppm in the ^13^C dimension, with 2K × 512 data points, measuring only 25% of all data points in the indirect dimension, by using non-uniform sampling (NUS). Selective excitation of ^13^C was achieved using a 180° Q3_surbop pulse.

### 3.6. Computational Details

The electronic structure and geometries of ligand precursors **1f**–**g** and NHC^R^ ligands were investigated using density functional theory at the B3LYP level [63,64] with the 6-311G* basis set. For the NHC–silver complexes, [Ag(NHC^R^)_2_]^3−^ anions of **2a**–**g** and Na_3_[Ag(NHC^β−Ala^)_2_], the Ag atom was described with the LANL2DZ basis set [65]. Molecular geometries were optimized without symmetry restrictions. Frequency calculations were carried out at the same level of theory to identify the stationary points as minima (zero imaginary frequencies). The GIAO method was used for NMR calculations (^1^H- and ^13^C-NMR isotropic shielding tensors), which were carried out at the 6-311+G(2d,p) level of theory. For calculations of ^109^Ag chemical shifts in NHC–silver complexes, a selection of these complexes (see Table S6) was optimized with the B3LYP functional [63,64], the 6-311G* basis set for light atoms, and LANL2DZ for silver ones [65]. Frequency calculations were carried out to verify that the optimized structures are energy minima. NMR calculations were carried out with the GIAO method at the B3LYP/6-311+G(2d,p) level of theory and using full electron DGDZVP basis set for silver atoms [53,66]. The use of a pseudopotential description for the basis set of silver atoms did not give reasonable results. For the calculation of the TEP parameter of NHC^R^ ligands, the approach used by Gusev was adopted (see details in Table S4) [58]. The Density Functional Theory (DFT) calculations were executed using the Gaussian 09 program package [67]. Coordinates of optimized compounds are collected in the Supplementary data (Tables S11–S13).

### 3.7. Single-Crystal X-ray Analysis

A summary of the crystallographic data and the structure refinement results for compounds **1f**, **2c**, and **2g** is given in Tables S8–S10. Crystals of suitable size for X-ray diffraction analysis were coated with dry perfluoropolyether, mounted on glass fibers, and fixed in a cold nitrogen stream (T = 193 K) to the goniometer head. Data collection was carried out on a Bruker-AXS, D8 QUEST ECO, PHOTON II area detector diffractometer (Billerica, MA, USA), using monochromatic radiation *λ*(Mo K_α_) = 0.71073 Å, by means of ω and φ scans with a width of 0.50 degrees. Data were reduced (SAINT [68]) and corrected for absorption effects using the multi-scan method (SADABS) [69]. Structures were solved by intrinsic phasing modification of direct methods (SHELXT [70]) and refined against all *F*^2^ data by full-matrix least-squares techniques (SHELXL-2018/3 [71]), minimizing *w*[*F*_o_^2^*-F*_c_^2^]^2^. All non-hydrogen atoms were refined anisotropically. Hydrogen atoms were included from calculated positions and refined, riding on their respective carbon atoms with isotropic displacement parameters. A search for solvent-accessible voids in the crystal structure of **2c** using SQUEEZE [72] showed a small volume of potential solvent of 403 Å^3^ (176 electron count), whose solvent content could not be identified or refined with the most severe restrictions. However, based on the volume and the electrons present, it would correspond to twenty molecules of disordered water. The corresponding CIF data represent SQUEEZE-treated structures with solvent molecules handled as a diffuse contribution to the overall scattering, without specific atom positions and excluded from the structural model. The SQUEEZE results were appended to the CIF. The corresponding crystallographic data were deposited with the Cambridge Crystallographic Data Centre as supplementary publications. CCDC 2335068 (**1f**), 2335069 (**2c**), and 2335070 (**2g**) contain the supplementary crystallographic data for this paper. The data can be obtained free of charge via https://www.ccdc.cam.ac.uk/structures/ (accessed on 22 September 2024).

## 4. Conclusions

Complexes Na_3_[Ag(NHC^R^)_2_], **2a**–**g** (NHC^R^ = 2,2′-(imidazol-2-ylidene)dicarboxylate-type *N*-heterocyclic carbene) were obtained by the reaction between Ag_2_O and imidazolium precursors HL^R^, **1a**–**g**, in the presence of aqueous sodium hydroxide. Complexes **2f**–**g** were spectroscopically and analytically characterized. These were similar to the previously reported complexes Na_3_[Ag(NHC^R^)_2_], **2a**–**e**, and the complete series **2a**–**g** was investigated. The imidazolium precursor **1f**, the homochiral Na_3_[Ag{(*S*,*S*)-NHC^Val^}_2_] **2c** and the complex Na_3_[Ag(NHC^β−Ala^)_2_], **2g** were structurally characterized by X-ray crystallography. Complexes **2c** and **2g** represent new examples of anionic bis(carbene)silver complexes in which the silver ion is coordinated by two carbenes in the expected linear fashion, while these NHC ligands adopt an unusually eclipsed disposition. These structural features, as well as the ^1^H and ^13^C NMR properties of the new complexes, were well described using DFT calculations. The antimicrobial properties of these silver complexes were investigated against Gram-negative bacteria *E. coli* and *P. aeruginosa*, as well as Gram-positive *S. aureus*. Based on the observed MIC and MBC values, complex **2b** exhibited the best antimicrobial properties against all tested strains. Remarkably, a relationship between chirality and antimicrobial effect was detected once again. The eutomer was the complex Na_3_[Ag{(*R*,*R*)-NHC^Val^}_2_], **2c′**, that showed lower MIC and MBC values for all strains compared to its enantiomer Na_3_[Ag{(*S*,*S*)-NHC^Val^}_2_], **2c**. This result confirms the preferential biocidal activity of one enantiomer, previously observed by us in related silver systems, and suggests a possible generalization of the chirality–antimicrobial trend. Additionally, the solution behavior of complexes **2** was analyzed by NMR and the ^109^Ag NMR chemical shifts were experimentally determined. A good correlation was found between these values and the theoretically calculated δ(^109^Ag) data. Furthermore, the electronic and steric characteristics of the NHC^R^ ligands were determined by calculating the TEP and %*V*_bur_ parameters. A correlation was established between the MO energy of the orbital responsible for carbene σ-donation and the TEP value of the NHC^R^ ligand.

## Figures and Tables

**Table 1:** Antimicrobial activities of complexes **2** evaluated by MIC and MBC (mM).

Complex	*E. coli*		*P. aeruginosa*		*S. aureus*	
	MIC	MBC	MIC	MBC	MIC	MBC
**2a**	0.167	0.200	0.167	>0.400	0.167	0.267
**2b**	0.200	0.200	0.167	0.200	0.134	0.134
**2c**	0.267	0.300	0.267	0.300	0.233	0.300
**2c′**	0.200	0.200	0.200	0.200	0.200	0.233
**2d**	0.267	0.300	0.300	0.300	0.267	0.300
**2e**	0.233	0.267	0.233	0.300	0.233	0.433
**2f**	0.233	0.267	0.233	0.300	0.233	0.300
**2g**	0.233	0.267	0.200	0.300	0.200	0.465
AgNO_3_	0.167	0.200	0.200	0.400	0.167	0.333

**Table 2:** Experimental δ(^109^Ag), calculated absolute σ(^109^Ag) values, and corrected calculated δ(^109^Ag) for the series of complexes **2**.

Complex	^109^Ag Resonances (ppm)		
	Experimental	Calculated	
	δ(^109^Ag)	σ(^109^Ag)	δ(^109^Ag)
**2a**	not observed	3550	598
**2b**	651	3539	609
**2c**	660	3501	646
**2d**	660	3563	585
**2e**	666	3486	661
**2f**	not observed	3508	639
**2g**	640	3476	671

**Table 3:** Electronic and steric properties of NHC^R^ ligands.

NHC^R^ Ligand	TEP (cm^−1^) ^a^	%*V*_bur_ ^b^
NHC^Gly^	2018.4	26.3
NHC^Ala^	2019.4	29.0
NHC^Val^	2019.6	30.2
NHC^Ile^	2020.3	30.4
NHC^Leu^	2021.5	28.6
NHC^β−Ala^	2021.6	26.2
NHC^GlyGly^	2039.6	26.2

^a^ Determined from optimized [Ni(CO)_3_(NHC^R^)]^2−^. ^b^ Determined from optimized [Ag(NHC^R^)_2_]^3−^.

## Data Availability

The data that support the findings of this study are available from the corresponding author upon reasonable request.

## Supplementary Materials

The following supporting information can be downloaded at: https://www.mdpi.com/article/10.3390/molecules29194608/s1, Figure S1. IR spectra of compounds **1f,g** and complexes **2f,g**. Figure S2. ^1^H NMR and ^13^C{^1^H] NMR spectra of compounds **1f**–**g**. Figure S3. Optimized structures of compounds **1f**–**g** and ligands NHC^β−Me^ and NHC^GlyGly^. Figure S4. Optimized structures of complexes: (a) [Ag(NHC^β−Me^)_2_]^3−^; (b) [Ag(NHC^GlyGly^)_2_]^3−^; and (c) Na_3_[Ag(NHC^β−Me^)_2_], **2g**. Two views of each complex are shown. Figure S5. Comparison of the calculated and experimental ^1^H and ^13^C NMR spectra for **2f**. Figure S6. Comparison of the calculated and experimental ^1^H and ^13^C NMR spectra for **2g**. Table S1. Comparison of experimental selected structural parameters of complexes **2c** and **2g** with the calculated parameters for the anions [Ag(NHC^R^)_2_]^3−^ of these complexes. Table S2. Selected structural data of **1f**, **2c** and **2g**. Bond distances (Å) and angles (°). Table S3. Specific rotations [α]_D_ for complexes **2**. Figure S7. Crystal packing views of complex **1f**. Figure S8. Crystal packing views of complex **2c**. Figure S9. Crystal packing views of complex **2g** viewed along *b* axis. Table S4. Optimized structures of complexes [Ni(CO)_3_(NHC^R^)]^2−^ and their calculated properties. Figure S10. Correlation between calculated TEP values (ν_CO_) of the NHC^R^ carbenes and the MO energy of the σ lone pair orbitals. Table S5. Percent buried volume, %*V*_bur_, of NHC^R^ ligands. Figure S11. Selected steric maps from optimized [Ag(NHC^R^)_2_]^3−^ complexes viewed along the Ag-C_carbene_ vector. Figure S12. ^1^H DOSY NMR spectra of **2b**–**c**. Figure S13. Band selective constant time ^1^H-^13^C HMBC NMR spectra (resolution enhanced by NUS) of **2c**. Figure S14. Correlation between calculated σ(^109^Ag) and experimental δ(^109^Ag) chemical shifts for selected NHC silver complexes. Table S6. Calculated σ(^109^Ag) and experimental δ(^109^Ag) data for selected NHC silver complexes. Table S7. Antimicrobial activities of complexes **2** evaluated by MIC and MBC (μg/mL). Figure S15. Qualitative hemolysis test of complex **2a** and several control chemicals (AgNO_3_, H_2_O_2_, sodium dodecyl sulfate, SDS and NaOH) for comparison. Table S8. Crystal data and structure refinement for **1f**. Table S9. Crystal data and structure refinement for **2c**. Table S10. Crystal data and structure refinement for **2g**. Table S11. Coordinates and optimized structures of NHC-silver complexes used for the correlation between calculated and experimental ^109^Ag NMR chemical shifts. Table S12. Coordinates of complexes [Ni(CO)_3_(NHC^R^)]^2−^ used for the determination of the Tolman Electronic Parameter (TEP). Table S13. Coordinates of the optimized structures.
