# Supplementary material for: Antimicrobial Activity of Anionic Bis(N-Heterocyclic Carbene) Silver Complexes
Source: Molecules. 2024 Sep 27;29(19):4608. doi: 10.3390/molecules29194608 (PMC11478204; doi:10.3390/molecules29194608)
Supplement: Supplementary file 1 [file molecules-29-04608-s001.zip › CheckCIF PLATON_JIB.pdf]

checkCIF (basic structural check) running

Checking for embedded fcf data in CIF ...
Found embedded fcf data in CIF. Extracting fcf data from uploaded CIF, please wait . . . .

checkCIF/PLATON (basic structural check)

Structure factors have been supplied for datablock(s) 1f, 2c, 2g

THIS REPORT IS FOR GUIDANCE ONLY. IF USED AS PART OF A REVIEW PROCEDURE FOR PUBLICATION, IT SHOULD NOT REPLACE THE EXPERTISE OF AN EXPERIENCED CRYSTALLOGRAPHIC REFEREE.

No syntax errors found. CIF dictionary
Please wait while processing .... Interpreting this report

Structure factor report

Datablock: 1f

Bond precision:	C-C = 0.0103 A		Wavelength=0.71073
Cell:	a=9.0622(17)	b=10.8523(14)	c=13.124(2)
	alpha=85.417(7)	beta=82.59(1)	gamma=89.894(8)
Temperature:	193 K		

	Calculated	Reported
Volume	1275.8(4)	1275.8(4)
Space group	P -1	P -1
Hall group	-P 1	-P 1
Moiety formula	C11 H14 N4 O6	C11 H14 N4 O6
Sum formula	C11 H14 N4 O6	C11 H14 N4 O6
Mr	298.26	298.26
Dx,g cm-3	1.553	1.553
Z	4	4
Mu (mm-1)	0.128	0.128
F000	624.0	624.0
F000'	624.36	
h,k,lmax	10,13,15	10,13,15
Nref	4604	4586
Tmin,Tmax	0.988,0.994	0.585,0.746
Tmin'	0.975	

Correction method= # Reported T Limits: Tmin=0.585 Tmax=0.746
AbsCorr = MULTI-SCAN

Data completeness= 0.996 Theta(max)= 25.249

R(reflections)= 0.1386(3454) wR2(reflections)= 0.3012(4586)

S = 1.162 Npar= 381

The following ALERTS were generated. Each ALERT has the format
test-name_ALERT_alert-type_alert-level.
Click on the hyperlinks for more details of the test.

Alert level B

PLAT097_ALERT_2_B Large Reported Max. (Positive) Residual Density 0.97 eA-3
PLAT213_ALERT_2_B Atom O10 has ADP max/min Ratio ..... 4.6 oblate
PLAT213_ALERT_2_B Atom C20 has ADP max/min Ratio ..... 4.3 prolat
PLAT340_ALERT_3_B Low Bond Precision on C-C Bonds .... 0.0103 Ang.

Alert level C

CRYSC01_ALERT_1_C The word below has not been recognised as a standard identifier.
colouless
CRYSC01_ALERT_1_C No recognised colour has been given for crystal colour.
DIFMX02_ALERT_1_C The maximum difference density is > 0.1*ZMAX*0.75
The relevant atom site should be identified.

RINTA01_ALERT_3_C The value of Rint is greater than 0.12
Rint given 0.156
PLAT020_ALERT_3_C The Value of Rint is Greater Than 0.12 .... 0.156 Report
PLAT082_ALERT_2_C High R1 Value .... 0.14 Report
PLAT084_ALERT_3_C High wR2 Value (i.e. > 0.25) .... 0.30 Report
PLAT213_ALERT_2_C Atom O2 has ADP max/min Ratio ..... 3.3 prolat
PLAT213_ALERT_2_C Atom O11 has ADP max/min Ratio ..... 3.6 prolat
PLAT220_ALERT_2_C NonSolvent Resd 1 C Ueq(max)/Ueq(min) Range 3.4 Ratio
PLAT353_ALERT_3_C Long N-H (N0.87,N1.01A) N3 - H3N . 1.01 Ang.
PLAT906_ALERT_3_C Large K Value in the Analysis of Variance ...... 36.064 Check
And 2 other PLAT906 Alerts
More ...
PLAT911_ALERT_3_C Missing FCF Refl Between Thmin & STh/L= 0.600 15 Report
-1 3 1, -1 4 1, 0-12 2, -2 4 2, -1 4 2, 5-11 3,
-1-12 4, 0-12 4, 5-11 4, 5-10 4, 6-10 5, -7 -6 7,
-4 -7 9, -7 3 9, -1 -7 12,
PLAT975_ALERT_2_C Check Calcd Resid. Dens. 1.06Ang From O9 . 0.60 eA-3
PLAT977_ALERT_2_C Check Negative Difference Density on H14 . -0.35 eA-3

Alert level G

PLAT003_ALERT_2_G Number of Uiso or Uij Restrained non-H Atoms ... 15 Report
PLAT007_ALERT_5_G Number of Unrefined Donor-H Atoms .... 6 Report
H3N H3O H4N H7N H8N H9O
PLAT083_ALERT_2_G SHELXL Second Parameter in WGHT Unusually Large 14.50 Why ?
PLAT177_ALERT_4_G The CIF-Embedded .res File Contains DELU Records 2 Report
PLAT178_ALERT_4_G The CIF-Embedded .res File Contains SIMU Records 2 Report
PLAT186_ALERT_4_G The CIF-Embedded .res File Contains ISOR Records 2 Report
PLAT188_ALERT_3_G A Non-default SIMU Restraint Value has been used 0.0020 Report
PLAT188_ALERT_3_G A Non-default SIMU Restraint Value has been used 0.0020 Report
PLAT192_ALERT_3_G A Non-default DELU Restraint Value for First Par 0.0020 Report
PLAT192_ALERT_3_G A Non-default DELU Restraint Value for First Par 0.0020 Report
PLAT480_ALERT_4_G Long H...A H-Bond Reported H8B ..O1 . 2.62 Ang.
PLAT860_ALERT_3_G Number of Least-Squares Restraints .... 97 Note
PLAT883_ALERT_1_G No Info/Value for _atom_sites_solution_primary . Please Do !
PLAT899_ALERT_4_G SHELXL2018 is Deprecated and Succeeded by SHELXL 2019/3 Note
PLAT909_ALERT_3_G Percentage of I>2sig(I) Data at Theta(Max) Still 60% Note
PLAT910_ALERT_3_G Missing # of FCF Reflection(s) Below Theta(Min). 2 Note
0 1 0, 0 0 1,
PLAT912_ALERT_4_G Missing # of FCF Reflections Above STh/L= 0.600 1 Note
PLAT961_ALERT_5_G Dataset Contains no Negative Intensities .... Please Check
PLAT965_ALERT_2_G The SHELXL WEIGHT Optimisation has not Converged Please Check
PLAT967_ALERT_5_G Note: Two-Theta Cutoff Value in Embedded .res .. 50.5 Degree
PLAT969_ALERT_5_G The 'Henn et al.' R-Factor-gap value .... 3.98 Note
Predicted wR2: Based on SigI**2 7.57 or SHELX Weight 27.08
PLAT978_ALERT_2_G Number C-C Bonds with Positive Residual Density. 0 Info

0 **ALERT level A** = Most likely a serious problem - resolve or explain
4 **ALERT level B** = A potentially serious problem, consider carefully
17 **ALERT level C** = Check. Ensure it is not caused by an omission or oversight
22 **ALERT level G** = General information/check it is not something unexpected

4 ALERT type 1 CIF construction/syntax error, inconsistent or missing data
13 ALERT type 2 Indicator that the structure model may be wrong or deficient
16 ALERT type 3 Indicator that the structure quality may be low
6 ALERT type 4 Improvement, methodology, query or suggestion
4 ALERT type 5 Informative message, check

Datablock: 2c

Bond precision:	C-C = 0.0218 A		Wavelength=0.71073
Cell:	a=16.038(9)	b=17.017(7)	c=18.571(10)
	alpha=82.98(2)	beta=71.63(3)	gamma=88.37(2)
Temperature:	193 K		

	Calculated	Reported
Volume	4774(4)	4774(4)
Space group	P 1	P 1
Hall group	P 1	P 1
Moiety formula	C26 H116 Ag N4 Na12 O50, 3(C26 H36 Ag N4 O8) [+ solvent]	C26 H116 Ag N4 Na12 O50, 3(C26 H36 Ag N4 O8)
Sum formula	C104 H224 Ag4 N16 Na12 O74 [+ solvent]	C104 H224 Ag4 N16 Na12 O74
Mr	3590.36	3590.34

Dx,g cm-3	1.249	1.249
Z	1	1
Mu (mm-1)	0.513	0.513
F000	1872.0	1872.0
F000'	1869.76	
h,k,lmax	19,20,22	19,20,22
Nref	34622[17311]	33843
Tmin,Tmax	0.884,0.950	0.649,0.746
Tmin'	0.836	

Correction method= # Reported T Limits: Tmin=0.649 Tmax=0.746
AbsCorr = MULTI-SCAN

Data completeness= 1.95/0.98 Theta(max)= 25.250

R(reflections)= 0.0708(28621) wR2(reflections)= 0.2119(33843)

S = 1.059 Npar= 1891

The following ALERTS were generated. Each ALERT has the format
test-name_ALERT_alert-type_alert-level.
Click on the hyperlinks for more details of the test.

Alert level B

PLAT342_ALERT_3_B Low Bond Precision on C-C Bonds .... 0.02185 Ang.
PLAT416_ALERT_2_B Short Intra D-H..H-D H9E ..H20M . 1.26 Ang.
x,y,z = 1_555 Check
And 3 other PLAT416 Alerts
More ...
1+x,1+y,z = 1_665 Check
PLAT416_ALERT_2_B Short Intra D-H..H-D H17F ..H31A . 1.52 Ang.
1+x,1+y,z = 1_665 Check
And 4 other PLAT416 Alerts
More ...
PLAT417_ALERT_2_B Short Inter D-H..H-D H8A ..H35A . 1.83 Ang.
x,1+y,z = 1_565 Check
PLAT420_ALERT_2_B D-H Bond Without Acceptor O1 --H1B . Please Check
And 8 other PLAT420 Alerts
More ...
PLAT971_ALERT_2_B Check Calcd Resid. Dens. 2.74Ang From O1 2.61 eA-3
PLAT987_ALERT_1_B The Flack x is >> 0 - Do a BASF/TWIN Refinement Please Check

---

Alert level C

PLAT094_ALERT_2_C Ratio of Maximum / Minimum Residual Density .... 2.84 Report
PLAT214_ALERT_2_C Atom C18D (Anion/Solvent) ADP max/min Ratio 4.6 prolat
PLAT220_ALERT_2_C NonSolvent Resd 1 C Ueq(max)/Ueq(min) Range 3.3 Ratio
And 2 other PLAT220 Alerts
More ...
PLAT221_ALERT_2_C Solv./Anion Resd 2 C Ueq(max)/Ueq(min) Range 5.3 Ratio
And 2 other PLAT221 Alerts
More ...
PLAT222_ALERT_3_C NonSolvent Resd 1 H Uiso(max)/Uiso(min) Range 5.9 Ratio
PLAT223_ALERT_4_C Solv./Anion Resd 2 H Ueq(max)/Ueq(min) Range 6.7 Ratio
And 2 other PLAT223 Alerts
More ...
PLAT241_ALERT_2_C High 'MainMol' Ueq as Compared to Neighbors of Na6 Check
And 4 other PLAT241 Alerts
More ...
PLAT242_ALERT_2_C Low 'MainMol' Ueq as Compared to Neighbors of Na5 Check
And 19 other PLAT242 Alerts
More ...
PLAT309_ALERT_2_C Single Bonded Oxygen (C-O > 1.3 Ang) .... O1C Check
PLAT361_ALERT_2_C Long C(sp3)-C(sp3) Bond C11D - C13D . 1.66 Ang.
PLAT361_ALERT_2_C Long C(sp3)-C(sp3) Bond C19D - C20D . 1.72 Ang.
PLAT363_ALERT_2_C Long C(sp3)-C(sp2) Bond C17D - C18D . 1.63 Ang.
PLAT430_ALERT_2_C Short Inter D...A Contact O4C ..O11 . 2.85 Ang.
x,y,z = 1_555 Check
PLAT481_ALERT_4_C Long D...A H-Bond Reported O3 ..O7D . 3.55 Ang.
PLAT910_ALERT_3_C Missing # of FCF Reflection(s) Below Theta(Min). 7 Note
1 0 0, -1 1 0, 0 1 0, 0 -1 1, 0 0 1, 1 0 1,
0 1 1,
PLAT911_ALERT_3_C Missing FCF Refl Between Thmin & STh/L= 0.600 213 Report
9 0 0, 12 3 0, 14 4 0, 13 7 0, 8 8 0, -5 12 0,
-6 -9 1, -12 -6 1, 1 -2 1, -11 -1 1, 1 -1 1, 13 -1 1,
-12 0 1, 14 0 1, -8 1 1, 11 11 1, 4 -7 2, 7 -6 2,

```
2 -3  2,   1 -1  2,   2 -1  2,   8 -1  2,   1  1  2,   7  2  2,
7  4  2,  14  4  2,   8  5  2,  13  5  2,  13  9  2,  10-12  3,
11-11  3,  -5-10  3,   7 -4  3,   2 -1  3,   3 -1  3,   0  0  3,
5  2  3,  13  5  3,  -7  7  3,  14  8  3,  12 10  3,  11 11  3,
13 11  3,  -6 13  3,  -6 14  3,   7-15  4,   6-14  4,   7 -6  4,
6 -3  4,   3 -1  4,   4 -1  4,   6 -1  4,   6  1  4,   8  1  4,
5  2  4,   6  3  4,   9  4  4,  16  4  4, -11  7  4, -10  9  4,
-9 10  4, -14 11  4,  11 11  4,   8-13  5,   8 -8  5, -15 -6  5,
5 -3  5, -10  1  5,   8  3  5,   9  4  5,  15  5  5,   6  7  5,
7  7  5,  14  8  5,  -8  9  5,   7  9  5,   6 12  5,   4 16  5,
7-15  6,  10-12  6,  15 -5  6, -15 -4  6,   8 -3  6,   5 -2  6,
9 -2  6,   6 -1  6,   9  0  6,   9  2  6,  10  3  6,  17  3  6,
3  4  6,   6  6  6,  -9  7  6,  -4  8  6, -14  9  6,  -6 11  6,
```

PLAT918_ALERT_3_C Reflection(s) with I(obs) much Smaller I(calc) . 3 Check
PLAT971_ALERT_2_C Check Calcd Resid. Dens. 1.46Ang From O30 1.63 eA-3
PLAT975_ALERT_2_C Check Calcd Resid. Dens. 1.06Ang From O11 . 1.22 eA-3
And 2 other PLAT975 Alerts
More ...
PLAT977_ALERT_2_C Check Negative Difference Density on H12M . -0.34 eA-3
And 6 other PLAT977 Alerts
More ...

Alert level G

PLAT003_ALERT_2_G Number of Uiso or Uij Restrained non-H Atoms ... 74 Report
PLAT004_ALERT_5_G Polymeric Structure Found with Maximum Dimension 2 Info
PLAT007_ALERT_5_G Number of Unrefined Donor-H Atoms .... 80 Report
H1A H1B H2E H2F H3E H3F H4E H4F H5A H5B H7A
H7B H8A H8B H9E H9F H10A H10B H12M H12N H13M H13N
PLAT033_ALERT_4_G Flack x Value Deviates > 3.0 * sigma from Zero . 0.034 Note
PLAT177_ALERT_4_G The CIF-Embedded .res File Contains DELU Records 1 Report
PLAT178_ALERT_4_G The CIF-Embedded .res File Contains SIMU Records 1 Report
PLAT186_ALERT_4_G The CIF-Embedded .res File Contains ISOR Records 1 Report
PLAT188_ALERT_3_G A Non-default SIMU Restraint Value has been used 0.0050 Report
PLAT192_ALERT_3_G A Non-default DELU Restraint Value for First Par 0.0050 Report
PLAT309_ALERT_2_G Single Bonded Oxygen (C-O > 1.3 Ang) .... O2B Check
And 2 other PLAT309 Alerts
More ...
PLAT480_ALERT_4_G Long H...A H-Bond Reported H3F ..O7D . 2.63 Ang.
PLAT480_ALERT_4_G Long H...A H-Bond Reported H32B ..O5D . 2.64 Ang.
PLAT606_ALERT_4_G Solvent Accessible VOID(S) in Structure .... ! Info
PLAT720_ALERT_4_G Number of Unusual/Non-Standard Labels .... 24 Note
H7A1 H7A2 H7A3 H8A1 H8A2 H8A3 H7B1 H7B2
H7B3 H8B1 H8B2 H8B3 H7C1 H7C2 H7C3 H8C1
H8C2 H8C3 H7D1 H7D2 H7D3 H8D1 H8D2 H8D3
PLAT773_ALERT_2_G Check long C-C Bond in CIF: C19D --C20D 1.71 Ang.
PLAT774_ALERT_1_G Check X-Y Bond in CIF: Na1 --Na11 .. 4.05 Ang.
And 2 other PLAT774 Alerts
More ...
PLAT780_ALERT_1_G Coordinates do not Form a Properly Connected Set Please Do !
PLAT860_ALERT_3_G Number of Least-Squares Restraints .... 447 Note
PLAT869_ALERT_4_G ALERTS Related to the Use of SQUEEZE Suppressed ! Info
PLAT883_ALERT_1_G No Info/Value for _atom_sites_solution_primary . Please Do !
PLAT899_ALERT_4_G SHELXL2018 is Deprecated and Succeeded by SHELXL 2019/3 Note
PLAT909_ALERT_3_G Percentage of I>2sig(I) Data at Theta(Max) Still 68% Note
PLAT912_ALERT_4_G Missing # of FCF Reflections Above STh/L= 0.600 1 Note
PLAT961_ALERT_5_G Dataset Contains no Negative Intensities .... Please Check
PLAT965_ALERT_2_G The SHELXL WEIGHT Optimisation has not Converged Please Check
PLAT967_ALERT_5_G Note: Two-Theta Cutoff Value in Embedded .res .. 50.5 Degree
PLAT969_ALERT_5_G The 'Henn et al.' R-Factor-gap value .... 4.25 Note
Predicted wR2: Based on SigI**2 4.99 or SHELX Weight 20.65
PLAT978_ALERT_2_G Number C-C Bonds with Positive Residual Density. 0 Info

---

0 **ALERT level A** = Most likely a serious problem - resolve or explain
22 **ALERT level B** = A potentially serious problem, consider carefully
57 **ALERT level C** = Check. Ensure it is not caused by an omission or oversight
32 **ALERT level G** = General information/check it is not something unexpected

6 ALERT type 1 CIF construction/syntax error, inconsistent or missing data
76 ALERT type 2 Indicator that the structure model may be wrong or deficient
9 ALERT type 3 Indicator that the structure quality may be low
15 ALERT type 4 Improvement, methodology, query or suggestion
5 ALERT type 5 Informative message, check

Datablock: 2g

Bond precision: C-C = 0.0037 A Wavelength=0.71073

Cell: a=8.6893(3) b=11.9503(4) c=15.6733(4)
alpha=77.673(1) beta=88.470(1) gamma=75.612(1)

Temperature: 193 K

	Calculated	Reported
Volume	1539.52(8)	1539.52(8)
Space group	P -1	P -1
Hall group	-P 1	-P 1
Moiety formula	C18 H32 Ag N4 Na3 O14, 3(H2 O)	C18 H32 Ag N4 Na3 O14, 3(H2 O)
Sum formula	C18 H38 Ag N4 Na3 O17	C18 H38 Ag N4 Na3 O17
Mr	759.36	759.36
Dx,g cm-3	1.638	1.638
Z	2	2
Mu (mm-1)	0.776	0.776
F000	780.0	780.0
F000'	778.81	
h,k,lmax	10,14,18	10,14,18
Nref	5584	5521
Tmin,Tmax	0.696,0.856	0.627,0.746
Tmin'	0.672	

Correction method= # Reported T Limits: Tmin=0.627 Tmax=0.746
AbsCorr = MULTI-SCAN

Data completeness= 0.989 Theta(max)= 25.246

R(reflections)= 0.0335(5384) wR2(reflections)= 0.0888(5521)

S = 1.087 Npar= 388

The following ALERTS were generated. Each ALERT has the format
test-name_ALERT_alert-type_alert-level.
Click on the hyperlinks for more details of the test.

Alert level B

PLAT416_ALERT_2_B Short Intra D-H..H-D H29 ..H113 . 1.43 Ang.
1-x,1-y,-z = 2_665 Check
PLAT416_ALERT_2_B Short Intra D-H..H-D H211 ..H214 . 1.19 Ang.
1-x,2-y,-z = 2_675 Check
PLAT417_ALERT_2_B Short Inter D-H..H-D H213 ..H216 . 2.02 Ang.
x,y,-1+z = 1_554 Check
PLAT417_ALERT_2_B Short Inter D-H..H-D H115 ..H217 . 1.84 Ang.
1-x,1-y,1-z = 2_666 Check
PLAT420_ALERT_2_B D-H Bond Without Acceptor O14 --H114 . Please Check
And 2 other PLAT420 Alerts
More ...

Alert level C

PLAT213_ALERT_2_C Atom O1 has ADP max/min Ratio ..... 3.1 prolat
PLAT250_ALERT_2_C Large U3/U1 Ratio for <U(i,j)> Tensor(Resd 1) 2.1 Note
PLAT415_ALERT_2_C Short Inter D-H..H-X H3 ..H116 . 2.14 Ang.
x,-1+y,z = 1_545 Check
PLAT911_ALERT_3_C Missing FCF Refl Between Thmin & STh/L= 0.600 61 Report
2 0 0, -1 1 0, 1 1 0, 3 1 0, 5 1 0, 2 2 0,
3 3 0, 1 6 0, 2 6 0, -3 -6 1, -2 -3 1, -3 -1 1,
-2 -1 1, -1 -1 1, -2 0 1, 2 0 1, 1 1 1, 2 1 1,
0 2 1, 2 2 1, -4 4 1, 1 5 1, 3 5 1, 1 8 1,
-2 -5 2, 0 -5 2, 0 -3 2, 1 -2 2, 3 -1 2, -3 0 2,
-2 0 2, 2 0 2, 1 2 2, 0 3 2, 1 3 2, 1 4 2,
2 4 2, 1 5 2, 2 6 2, -2 -3 3, -2 -1 3, -1 0 3,
1 0 3, 3 0 3, 0 3 3, 1 3 3, 2 3 3, -4 -6 4,
2 -6 4, 1 -5 4, -1 -2 4, 2 -1 4, -1 2 4, 2 5 4,
2 6 4, -3 -3 5, 0 -2 6, 0 0 6, 2 0 6, -1 1 7,
-2 -1 8,
PLAT913_ALERT_3_C Missing # of Very Strong Reflections in FCF .... 9 Note
2 0 0, 3 1 0, -2 -3 1, -2 0 1, 2 0 1, -3 0 2,
1 5 2, 2 3 3, 0 0 6,
PLAT975_ALERT_2_C Check Calcd Resid. Dens. 1.01Ang From O13 . 0.62 eA-3
And 7 other PLAT975 Alerts
More ...
PLAT976_ALERT_2_C Check Calcd Resid. Dens. 0.83Ang From O13 . -0.73 eA-3
And 10 other PLAT976 Alerts
More ...

PLAT977_ALERT_2_C Check Negative Difference Density on H17A . -0.32 eA-3
And 10 other PLAT977 Alerts
More ...

Alert level G

PLAT004_ALERT_5_G Polymeric Structure Found with Maximum Dimension 3 Info
PLAT007_ALERT_5_G Number of Unrefined Donor-H Atoms .... 18 Report
H19 H29 H110 H111 H112 H113 H114 H210 H211 H212 H213
H214 H115 H215 H116 H216 H117 H217
PLAT154_ALERT_1_G The s.u.'s on the Cell Angles are Equal ..(Note) 0.001 Degree
PLAT232_ALERT_2_G Hirshfeld Test Diff (M-X) Ag1 --C10 . 5.1 s.u.
PLAT480_ALERT_4_G Long H...A H-Bond Reported H19 ..O13 . 2.64 Ang.
PLAT764_ALERT_4_G Overcomplete CIF Bond List Detected (Rep/Expd) . 1.10 Ratio
PLAT883_ALERT_1_G No Info/Value for _atom_sites_solution_primary . Please Do !
PLAT899_ALERT_4_G SHELXL2018 is Deprecated and Succeeded by SHELXL 2019/3 Note
PLAT909_ALERT_3_G Percentage of I>2sig(I) Data at Theta(Max) Still 95% Note
PLAT910_ALERT_3_G Missing # of FCF Reflection(s) Below Theta(Min). 2 Note
0 1 0, 0 0 1,
PLAT961_ALERT_5_G Dataset Contains no Negative Intensities .... Please Check
PLAT967_ALERT_5_G Note: Two-Theta Cutoff Value in Embedded .res .. 50.5 Degree
PLAT969_ALERT_5_G The 'Henn et al.' R-Factor-gap value .... 2.93 Note
Predicted wR2: Based on SigI**2 3.03 or SHELX Weight 8.47
PLAT978_ALERT_2_G Number C-C Bonds with Positive Residual Density. 1 Info

---

0 **ALERT level A** = Most likely a serious problem - resolve or explain
7 **ALERT level B** = A potentially serious problem, consider carefully
35 **ALERT level C** = Check. Ensure it is not caused by an omission or oversight
14 **ALERT level G** = General information/check it is not something unexpected

2 ALERT type 1 CIF construction/syntax error, inconsistent or missing data
42 ALERT type 2 Indicator that the structure model may be wrong or deficient
4 ALERT type 3 Indicator that the structure quality may be low
3 ALERT type 4 Improvement, methodology, query or suggestion
5 ALERT type 5 Informative message, check

It is advisable to attempt to resolve as many as possible of the alerts in all categories. Often the minor alerts point to easily fixed oversights, errors and omissions in your CIF or refinement strategy, so attention to these fine details can be worthwhile. In order to resolve some of the more serious problems it may be necessary to carry out additional measurements or structure refinements. However, the purpose of your study may justify the reported deviations and the more serious of these should normally be commented upon in the discussion or experimental section of a paper or in the "special_details" fields of the CIF. checkCIF was carefully designed to identify outliers and unusual parameters, but every test has its limitations and alerts that are not important in a particular case may appear. Conversely, the absence of alerts does not guarantee there are no aspects of the results needing attention. It is up to the individual to critically assess their own results and, if necessary, seek expert advice.

Publication of your CIF in IUCr journals

A basic structural check has been run on your CIF. These basic checks will be run on all CIFs submitted for publication in IUCr journals (*Acta Crystallographica*, *Journal of Applied Crystallography*, *Journal of Synchrotron Radiation*); however, if you intend to submit to *Acta Crystallographica Section C* or *E* or *IUCrData*, you should make sure that full publication checks are run on the final version of your CIF prior to submission.

Publication of your CIF in other journals

Please refer to the *Notes for Authors* of the relevant journal for any special instructions relating to CIF submission.

PLATON version of 06/01/2024; check.def file version of 05/01/2024

Datablock 1f - ellipsoid plot

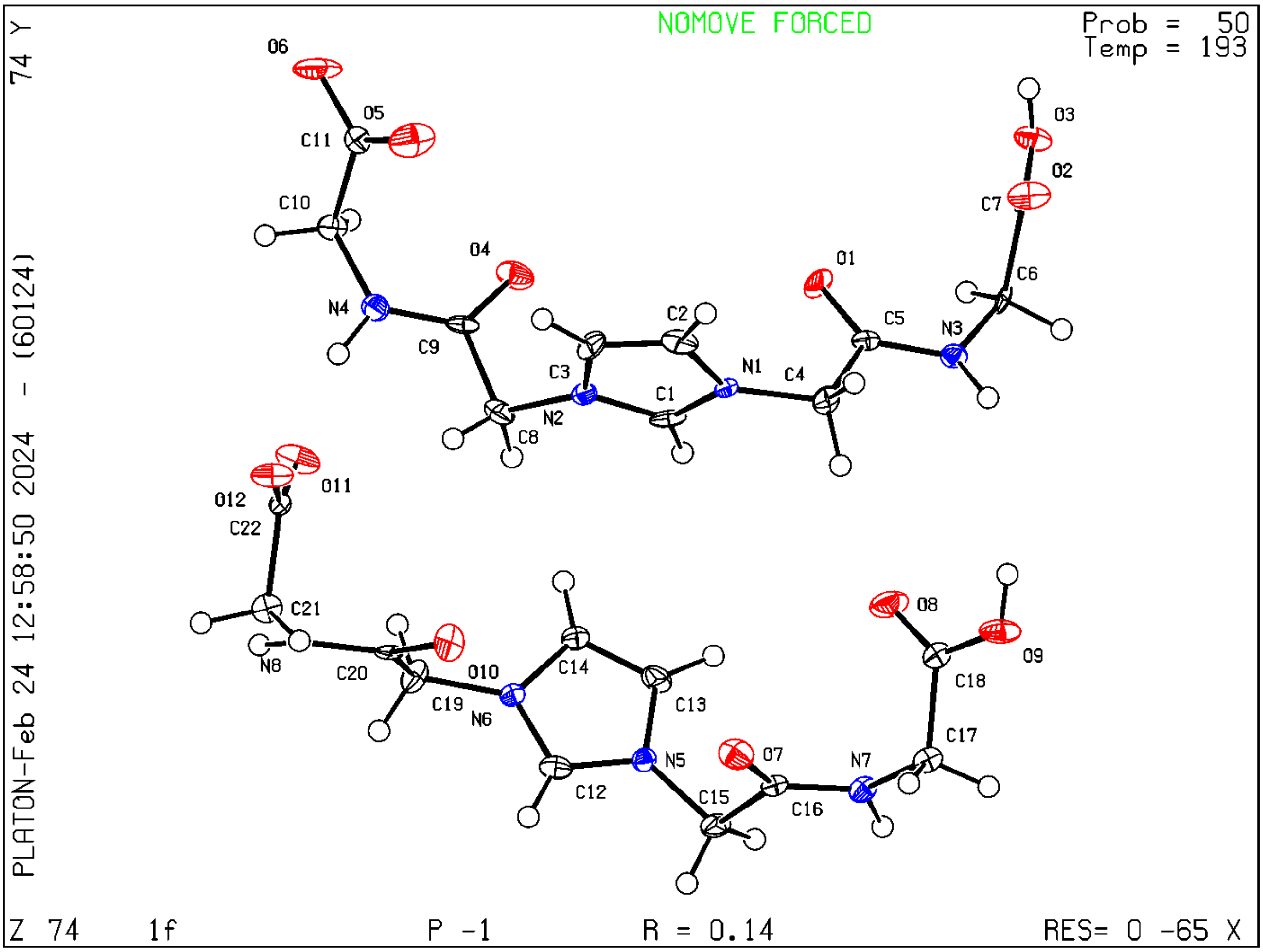

Datablock 2c - ellipsoid plot

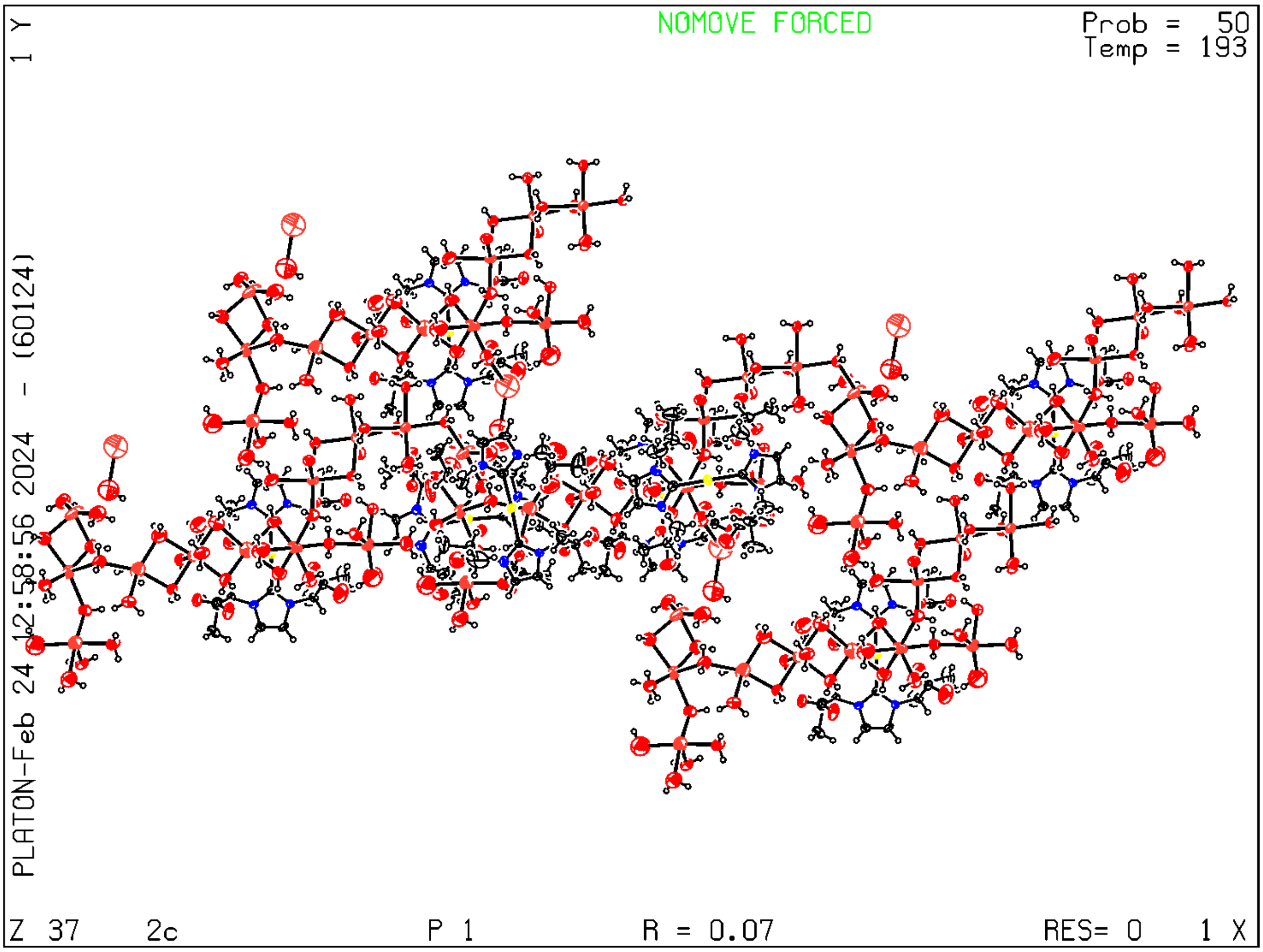

Datablock 2g - ellipsoid plot

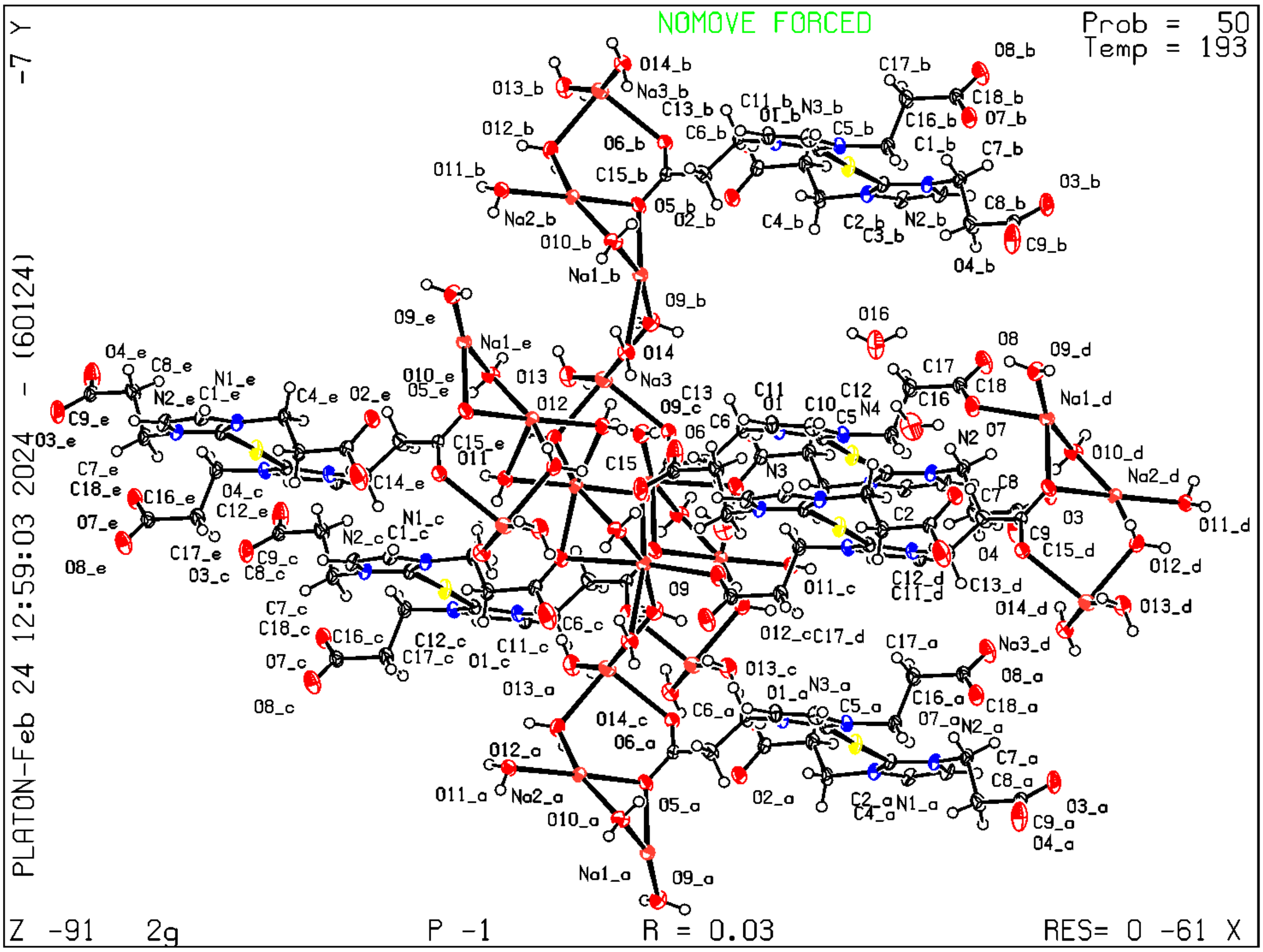

Download CIF editor (publCIF) from the IUCr
Download CIF editor (enCIFer) from the CCDC
Test a new CIF entry
