# Supplementary material for: Antimicrobial Activity of Anionic Bis(N-Heterocyclic Carbene) Silver Complexes
Source: Molecules. 2024 Sep 27;29(19):4608. doi: 10.3390/molecules29194608 (PMC11478204; doi:10.3390/molecules29194608)
Supplement: Supplementary file 1 [file molecules-29-04608-s001.zip › SuppMat_Ag_NHC_antimic_REV.pdf]

SUPPLEMENTARY MATERIAL

Antimicrobial activity of anionic bis(N-heterocyclic carbene)silver complexes

Carlos J. Carrasco [1,*], Francisco Montilla [1], Eduardo Villalobo [2], Manuel Angulo [3], Eleuterio Álvarez [4] and Agustín Galindo [1,*]

1 Departamento de Química Inorgánica, Facultad de Química, Universidad de Sevilla, 41012 Sevilla, Spain
2 Departamento de Microbiología, Facultad de Biología, Universidad de Sevilla, 41012 Sevilla, Spain.
3 Servicio de Resonancia Magnética Nuclear, CITIUS, Universidad de Sevilla, Avenida Reina Mercedes, 4b, 41012 Sevilla, Spain.
4 Instituto de Investigaciones Químicas, CSIC-Universidad de Sevilla, Avda. Américo Vespucio 49, 41092 Sevilla, Spain.
* Correspondence: galindo@us.es

Figure S1. IR spectra of compounds **1f,g** and complexes **2f,g**.

Figure S2. ^{1}H NMR and ^{13}C{^{1}H] NMR spectra of compounds **1f-g**.

Figure S3. Optimized structures of compounds **1f-g** and ligands $NHC^{\beta\text{-}Me}$ and NHC^{GlyGly}.

Figure S4. Optimized structures of complexes: (a) $[Ag(NHC^{\beta\text{-}Me})_2]^{3-}$; (b) $[Ag(NHC^{GlyGly})_2]^{3-}$; and (c) $Na_3[Ag(NHC^{\beta\text{-}Me})_2]$, **2g**. Two views of each complex are shown.

Figure S5. Comparison of the calculated and experimental ^{1}H and ^{13}C NMR spectra for **2f**.

Figure S6. Comparison of the calculated and experimental ^{1}H and ^{13}C NMR spectra for **2g**.

Table S1. Comparison of experimental selected structural parameters of complexes **2c** and **2g** with the calculated parameters for the anions $[Ag(NHC^{R})_2]^{3-}$ of these complexes.

Table S2. (a) Selected structural data of complex **1f**. (b) Selected structural data of complex **2c**. (c) Selected structural data of complex **2g**. Bond distances (Å) and angles (º).

Table S3. Specific rotations $[\alpha]_D$ for complexes **2**.

Figure S7. Crystal packing views of complex **1f**.

Figure S8. Crystal packing views of complex **2c**.

Figure S9. Crystal packing views of complex **2g** viewed along *b* axis.

Table S4. Optimized structures of complexes $[Ni(CO)_3(NHC^R)]^{2-}$ and their calculated properties.

Figure S10. Correlation between calculated TEP values (ν_{CO}) of the NHC^R carbenes and the MO energy of the σ lone pair orbitals.

Table S5. Percent buried volume, $\%V_{bur}$, of NHC^R ligands.

Figure S11. Selected steric maps from optimized $[Ag(NHC^R)_2]^{3-}$ complexes viewed along the Ag-$C_{carbene}$ vector.

Figure S12. ^{1}H DOSY NMR spectra of **2b**-**c**.

Figure S13. Band selective constant time ^{1}H-^{13}C HMBC NMR spectra (resolution enhanced by NUS) of **2c**.

Figure S14. Correlation between calculated σ(^{109}Ag) and experimental δ(^{109}Ag) chemical shifts for selected NHC silver complexes.

Table S6. Calculated σ(^{109}Ag) and experimental δ(^{109}Ag) data for selected NHC silver complexes.

Table S7. Antimicrobial activities of complexes **2** evaluated by MIC and MBC (μg/mL).

Figure S15. Qualitative hemolysis test of complex **2a** and several control chemicals ($AgNO_3$, H_2O_2, sodium dodecyl sulfate, SDS and NaOH) for comparison.

Table S8. Crystal data and structure refinement for **1f**.

Table S9. Crystal data and structure refinement for **2c**.

Table S10. Crystal data and structure refinement for **2g**.

Table S11. Coordinates and optimized structures of NHC-silver complexes used for the correlation between calculated and experimental ^{109}Ag NMR chemical shifts.

Table S12. Coordinates of complexes $[Ni(CO)_3(NHC^R)]^{2-}$ used for the determination of the Tolman Electronic Parameter (TEP).

Table S13. Coordinates of the optimized structures.

Figure S1. IR spectra of compounds **1f,g** and complexes **2f,g**.

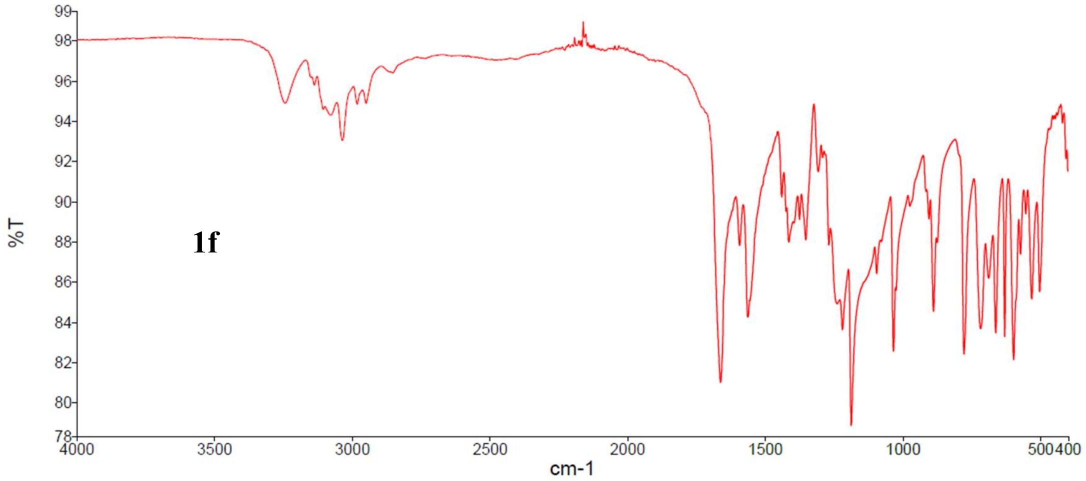

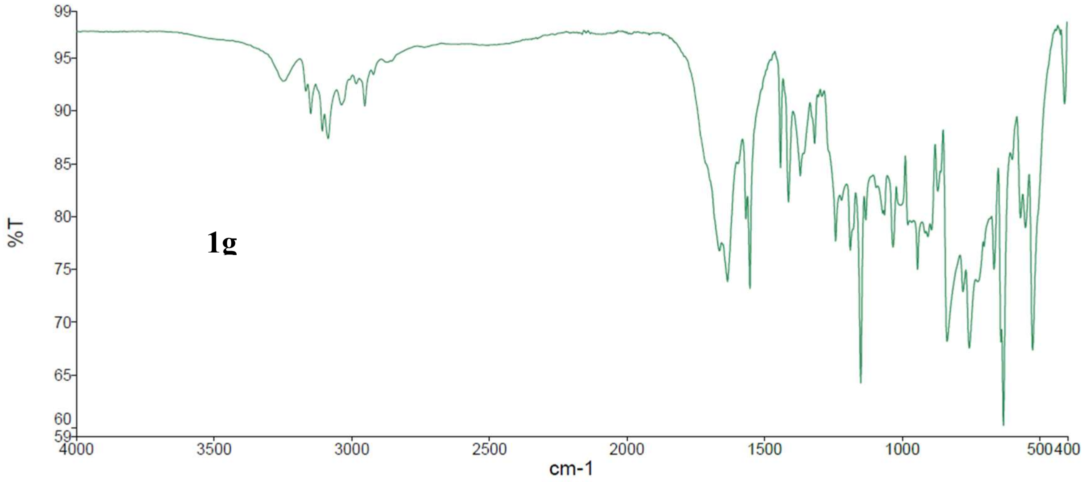

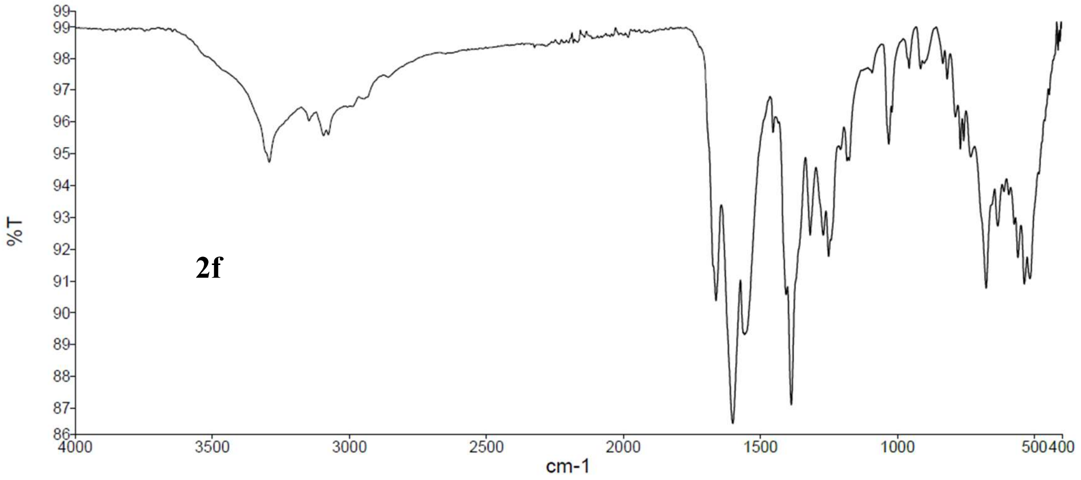

2f

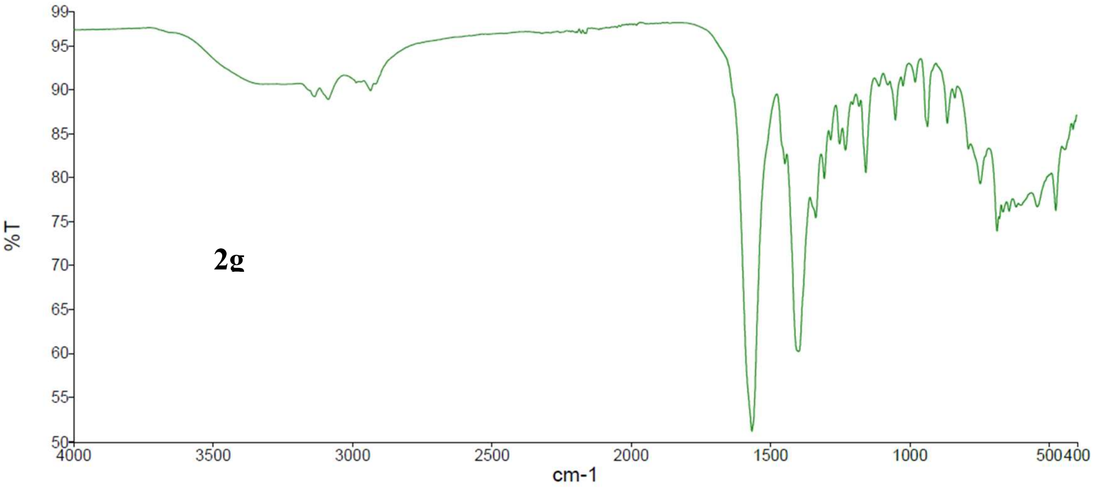

2g

Figure S2. ^{1}H NMR spectra of compounds **1f**-**g**.

1f

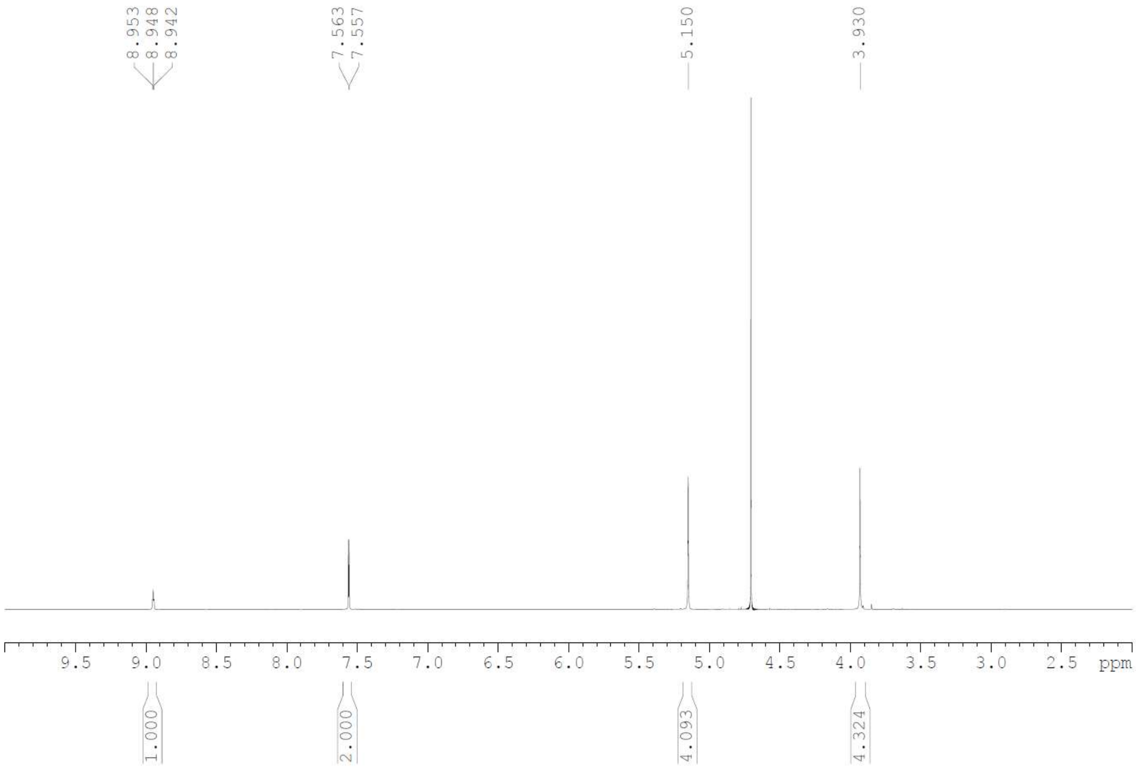

1g

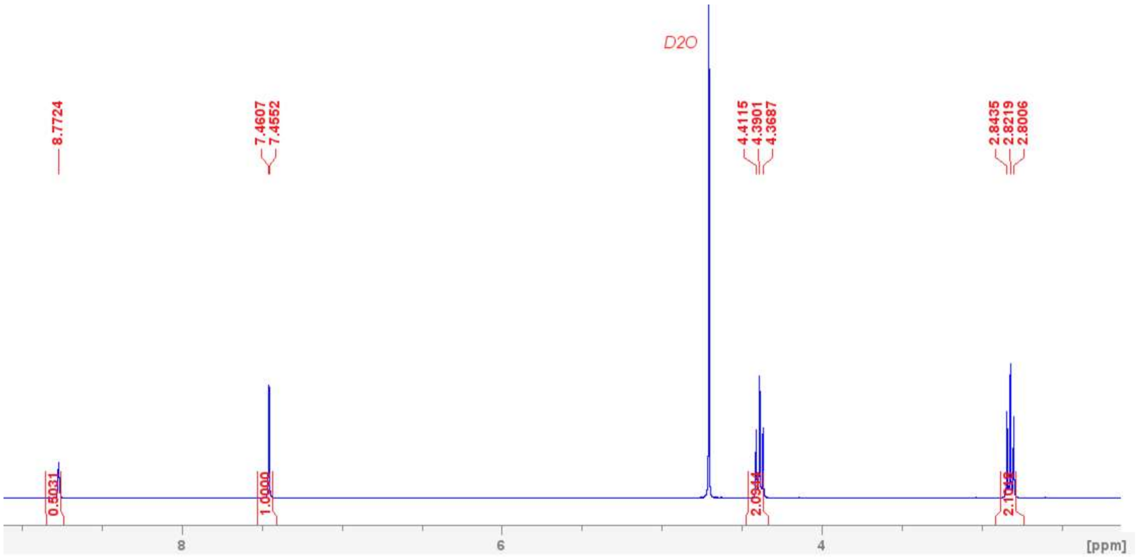

Figure S2. $^{13}C\{^1H\}$ NMR spectra of compounds **1f-g**.

1f

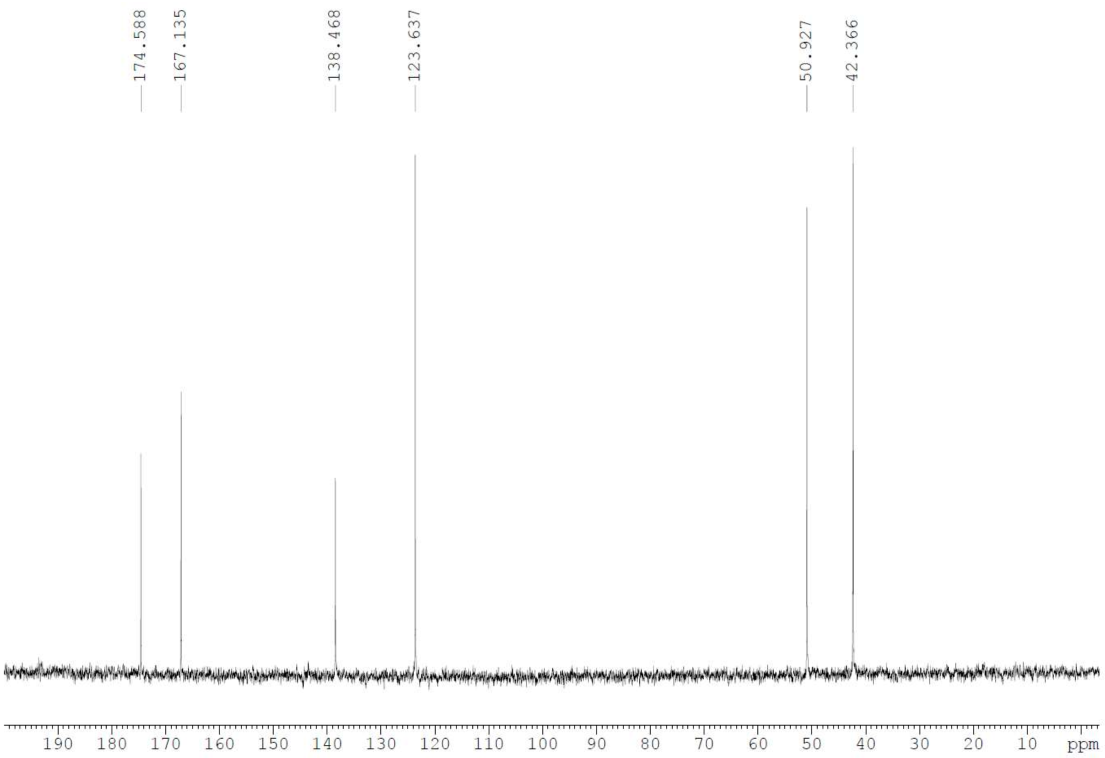

1g

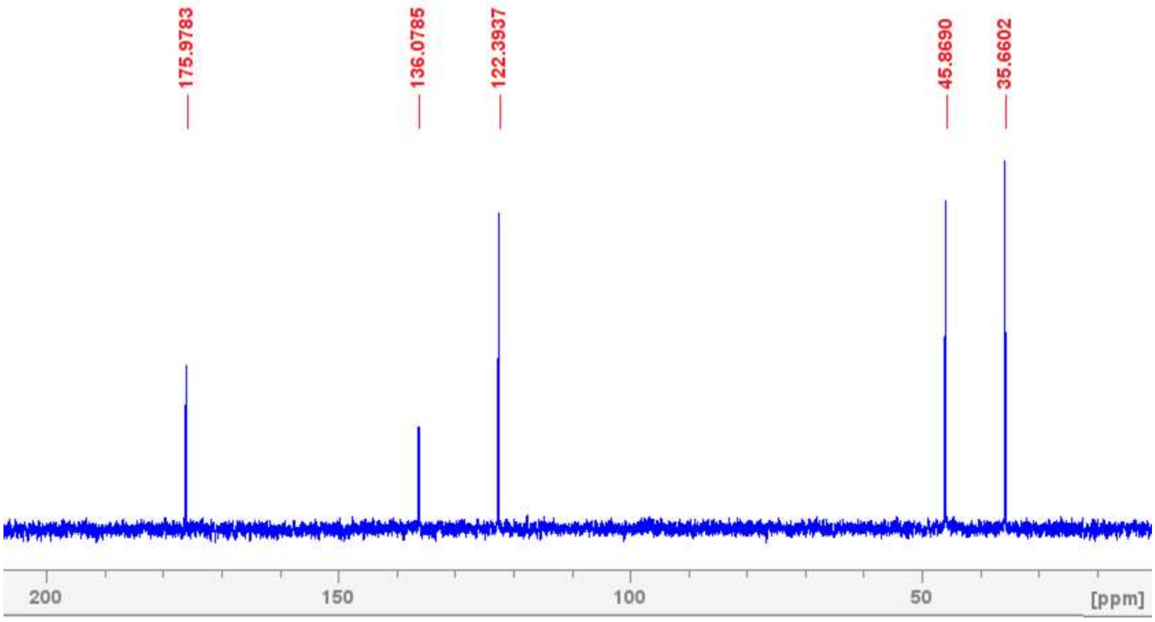

Figure S3. Optimized structures of compounds **1f-g** and ligands NHC^{GlyGly} and NHC$^{β\text{-}Me}$.

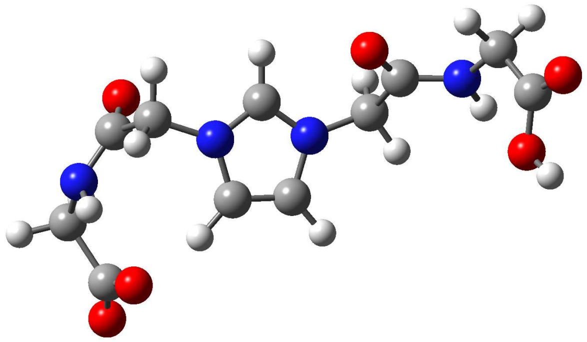

1f

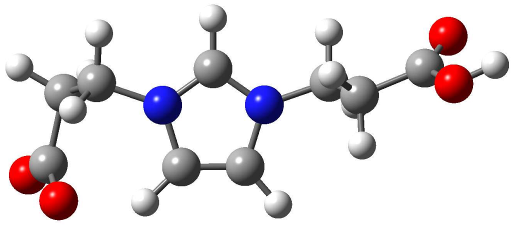

1g

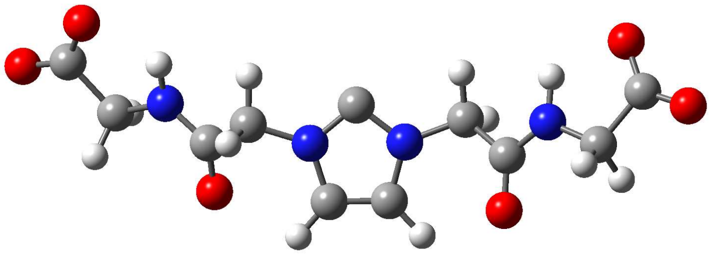

NHC^{GlyGly}

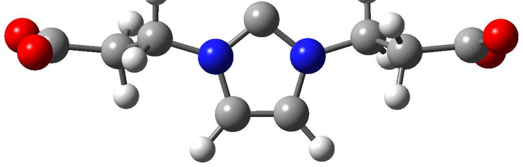

NHC$^{β\text{-}Me}$

Figure S4. Optimized structures of complexes: (a) $[Ag(NHC^{\beta\text{-}Me})_2]^{3-}$; (b) $[Ag(NHC^{GlyGly})_2]^{3-}$; and (c) $Na_3[Ag(NHC^{\beta\text{-}Me})_2]$, **2g**. Two views of each complex are shown.

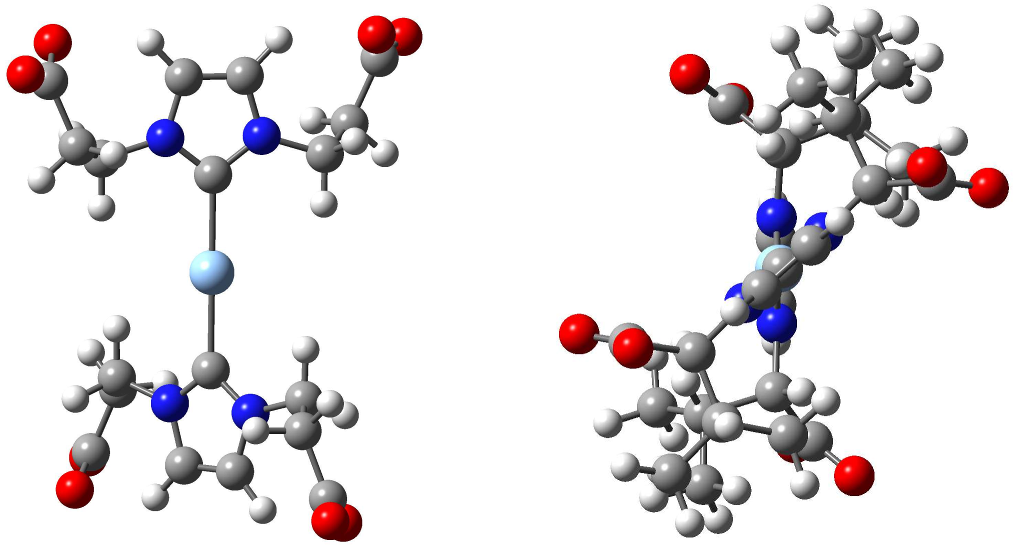

(a)

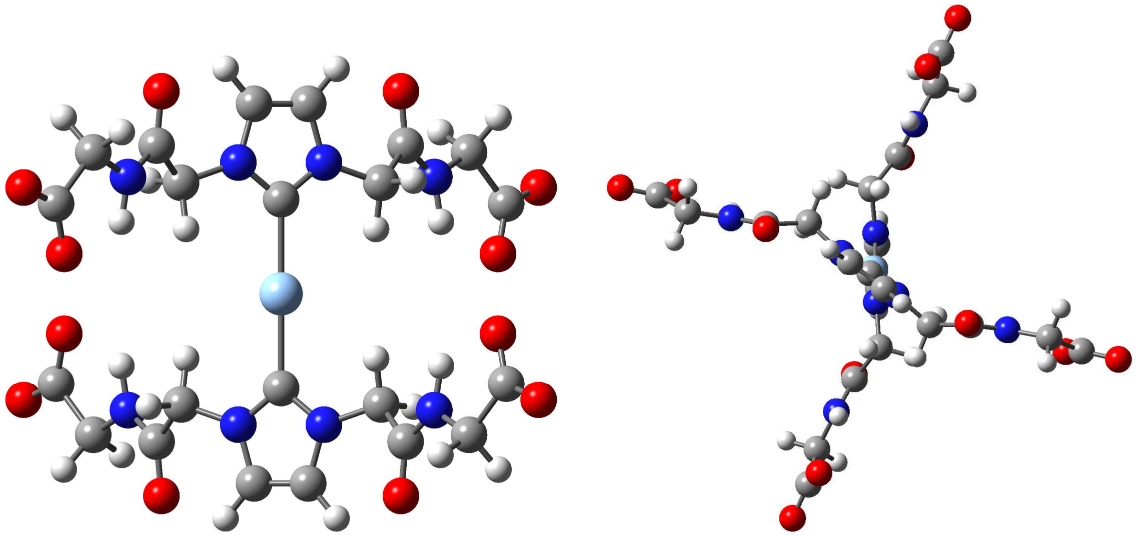

(b)

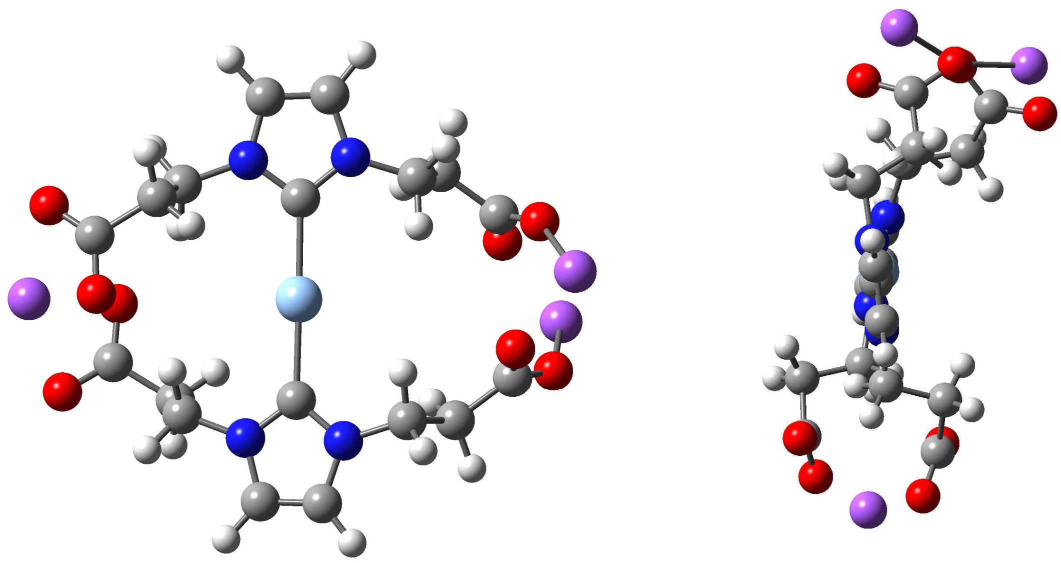

(c)

Figure S5. Comparison of the calculated and experimental ^{1}H and ^{13}C NMR spectra for **2f**.

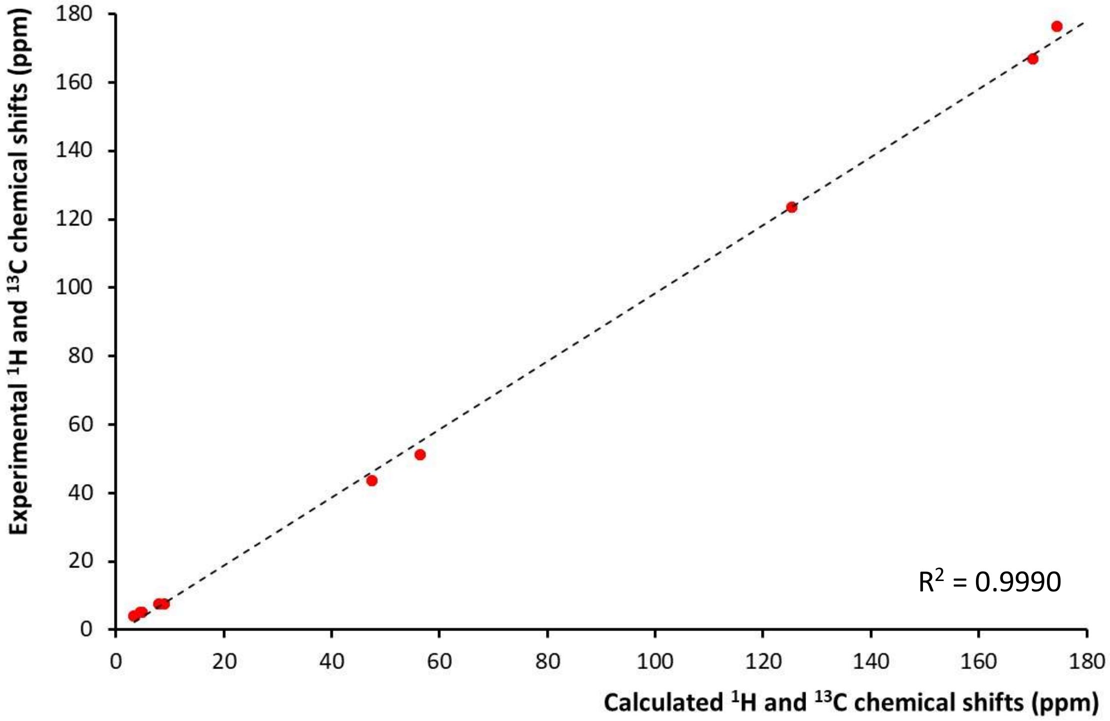

Figure S6. Comparison of the calculated and experimental ^1H and ^{13}C NMR spectra for **2g**.

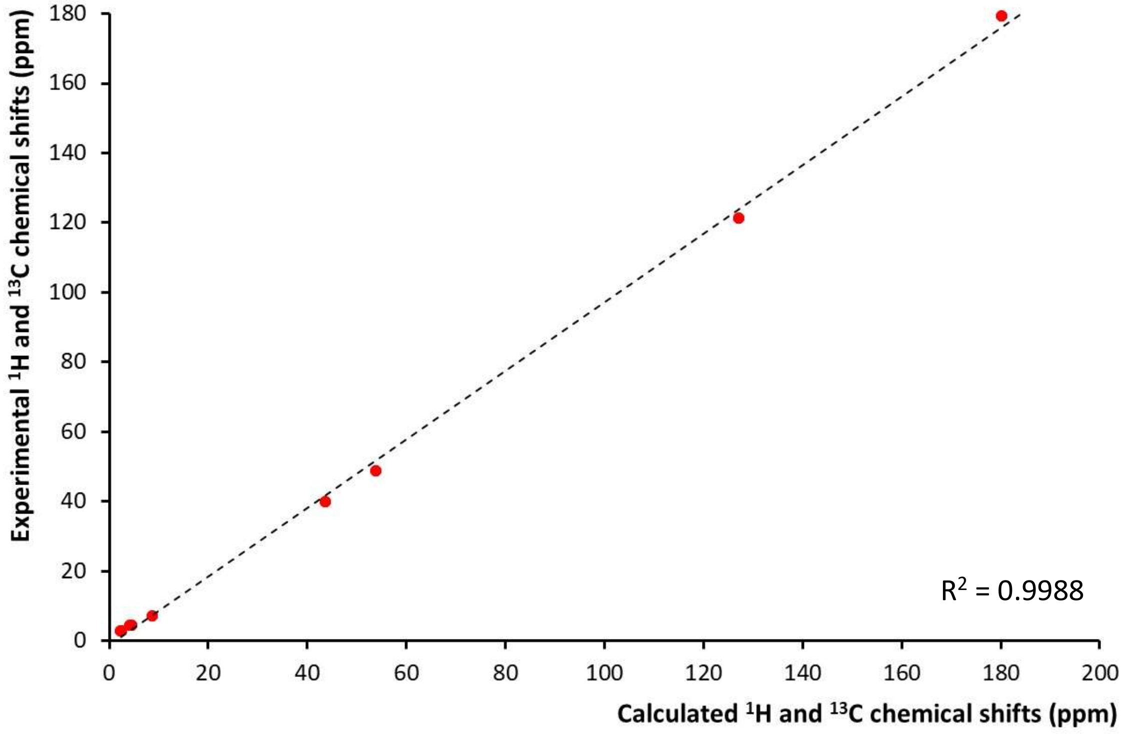

Table S1. Comparison of experimental selected structural parameters of complexes **2c** and **2g** with the calculated parameters for the anions $[Ag(NHC^{R})_2]^{3-}$ of these complexes.

**Bond distances (Å) and angles (º)**	**2c**		**2g**	
	**Exp.**	**Calc.**	**Exp.**	**Calc.**
Ag-$C_{carbene}$	2.12(1)<br>2.15(1)	2.171	2.081(2)<br>2.084(2)	2.151
C-$O_{carboxylate}$	1.24(2)<br>1.34(2)<br>1.28(1)<br>1.26(2)<br>1.28(1)<br>1.29(2)<br>1.21(2)<br>1.29(2)	1.248<br>1.258	1.245(3)<br>1.253(3)<br>1.253(3)<br>1.232(3)<br>1.249(3)<br>1.263(3)<br>1.252(3)<br>1.244(3)	1.246<br>1.259
$C_{carbene}\overset{...}{-}N$	1.41(2)<br>1.34(2)<br>1.39(1)<br>1.41(2)	1.365	1.350(3)<br>1.352(3)	1.359
$C\overset{...}{-}C$ (NHC ring)	1.37(2)<br>1.39(2)	1.355	1.348(4)<br>1.352(4)	1.358
$C_{carbene}$-Ag-$C_{carbene}$	174.5(4)	179.1	175.39(9)	179.9

Table S2. (a) Selected structural data of complex **1f** [bond distances (Å) and angles (º)].

O(1)-C(5)	1.235(8)	O(10)-C(20)	1.241(8)
O(2)-C(7)	1.231(8)	O(11)-C(22)	1.239(8)
O(3)-C(7)	1.265(8)	O(12)-C(22)	1.234(8)
O(4)-C(9)	1.251(9)		
O(5)-C(11)	1.199(9)	O(2)-C(7)-O(3)	124.5(6)
O(6)-C(11)	1.279(9)	O(5)-C(11)-O(6)	125.3(7)
O(7)-C(16)	1.241(9)	O(8)-C(18)-O(9)	125.6(7)
O(8)-C(18)	1.202(9)	O(12)-C(22)-O(11)	127.6(7)
O(9)-C(18)	1.301(9)		

Table S2. (b) Selected structural data of complex **2c** [bond distances (Å) and angles (º)].

Ag(1A)-C(14A)	2.123(11)	O(3C)-C(10C)	1.268(15)
Ag(1A)-C(1A)	2.148(12)	O(4C)-C(10C)	1.294(15)
O(1A)-C(5A)	1.241(17)	O(5C)-C(18C)	1.270(15)
O(2A)-C(5A)	1.336(17)	O(6C)-C(18C)	1.297(16)
O(3A)-C(10A)	1.278(14)	O(7C)-C(23C)	1.22(3)
O(4A)-C(10A)	1.258(15)	O(8C)-C(23C)	1.26(2)
O(5A)-C(18A)	1.280(14)	Ag(1D)-C(1D)	2.149(11)
O(6A)-C(18A)	1.287(15)	Ag(1D)-C(14D)	2.178(10)
O(7A)-C(23A)	1.21(2)	O(1D)-C(5D)	1.250(18)
O(8A)-C(23A)	1.29(2)	O(2D)-C(5D)	1.15(2)
Ag(1B)-C(14B)	2.163(11)	O(3D)-C(10D)	1.20(2)
Ag(1B)-C(1B)	2.172(12)	O(4D)-C(10D)	1.24(2)
O(1B)-C(5B)	1.189(17)	O(5D)-C(18D)	1.25(2)
O(1B)-Na(6)	2.341(15)	O(6D)-C(18D)	1.34(2)
O(2B)-C(5B)	1.306(18)	O(6D)-Na(10)	2.911(19)
O(3B)-C(10B)	1.297(15)	O(7D)-C(23D)	1.270(15)
O(3B)-Na(3)	2.603(10)	O(8D)-C(23D)	1.267(14)
O(4B)-C(10B)	1.249(15)		
C(20B)-C(19B)	1.538(18)	C(14A)-Ag(1A)-C(1A)	174.5(4)
Ag(1C)-C(1C)	2.130(11)	C(14B)-Ag(1B)-C(1B)	175.3(4)
Ag(1C)-C(14C)	2.132(12)	C(1C)-Ag(1C)-C(14C)	178.4(4)
O(1C)-C(5C)	1.303(17)	C(1D)-Ag(1D)-C(14D)	175.6(4)
O(2C)-C(5C)	1.247(17)		

Symmetry transformations used to generate equivalent atoms: #1 x+1,y+1,z; #2 x+1,y,z; #3 x-1,y-1,z; #4 x-1,y,z.

Table S2. (c) Selected structural data of complex **2g** [bond distances (Å) and angles (º)].

Ag(1)-C(1)	2.081(2)	N(1)-C(1)	1.350(3)
Ag(1)-C(10)	2.084(2)	N(1)-C(2)	1.382(3)
O(1)-C(6)	1.245(3)	N(1)-C(4)	1.472(3)
O(2)-C(6)	1.253(3)	N(2)-C(1)	1.350(3)
O(3)-C(9)	1.253(3)	N(2)-C(3)	1.381(3)
O(4)-C(9)	1.232(3)	N(2)-C(7)	1.463(3)
O(5)-C(15)	1.249(3)	N(3)-C(10)	1.350(3)
O(6)-C(15)	1.263(3)	N(3)-C(11)	1.380(3)
O(7)-C(18)	1.252(3)	N(3)-C(13)	1.467(3)
O(8)-C(18)	1.244(3)	N(4)-C(10)	1.352(3)
Na(1)-O(9)	2.303(2)	N(4)-C(12)	1.378(3)
Na(1)-O(5)	2.3284(19)	N(4)-C(16)	1.461(3)
Na(1)-O(7)#1	2.341(2)	C(2)-C(3)	1.348(4)
Na(1)-O(10)	2.388(2)	C(4)-C(5)	1.511(3)
Na(1)-O(2)#2	2.5723(19)	C(5)-C(6)	1.516(3)
Na(1)-O(14)#3	2.724(2)	C(7)-C(8)	1.521(3)
Na(2)-O(11)	2.359(2)	C(8)-C(9)	1.530(3)
Na(2)-O(12)	2.3638(19)	C(11)-C(12)	1.352(4)
Na(2)-O(11)#4	2.3880(19)	C(13)-C(14)	1.500(3)
Na(2)-O(10)	2.3963(19)	C(14)-C(15)	1.515(3)
Na(2)-O(2)#2	2.450(2)	C(16)-C(17)	1.519(4)
Na(2)-O(5)	2.492(2)	C(17)-C(18)	1.526(3)
Na(3)-O(14)	2.342(2)	C(1)-Ag(1)-C(10)	175.39(9)
Na(3)-O(12)	2.358(2)	O(1)-C(6)-O(2)	123.5(2)
Na(3)-O(13)	2.383(2)	O(4)-C(9)-O(3)	125.1(2)
Na(3)-O(9)#5	2.465(2)	O(5)-C(15)-O(6)	124.5(2)
Na(3)-O(6)	2.489(2)	O(8)-C(18)-O(7)	125.5(2)
Na(3)-O(1)#6	2.757(3)		

Symmetry transformations used to generate equivalent atoms: #1 -x+1,-y+1,-z+1; #2 -x+1,-y+1,-z; #3 x-1,y,z; #4 -x+1,-y+2,-z; #5 x+1,y,z; #6 -x+2,-y+1,-z.

Table S3. Specific rotations $[\alpha]_D$ for complexes **2**.

**Complex $Na_3[Ag(NHC^R)_2]$**	**$[\alpha]_D$**
**2b**	6.5° ± 0.6
**2b'**	-1.4° ± 0.3
**2c**	4.1° ± 0.2
**2c'**	-1.5° ± 0.2
**2d**	6.8° ± 0.2
**2e**	18.4° ± 0.3

[a] $[\alpha]_D$ determined in water at room temperature.

Figure S7. Crystal packing views of complex **1f**.

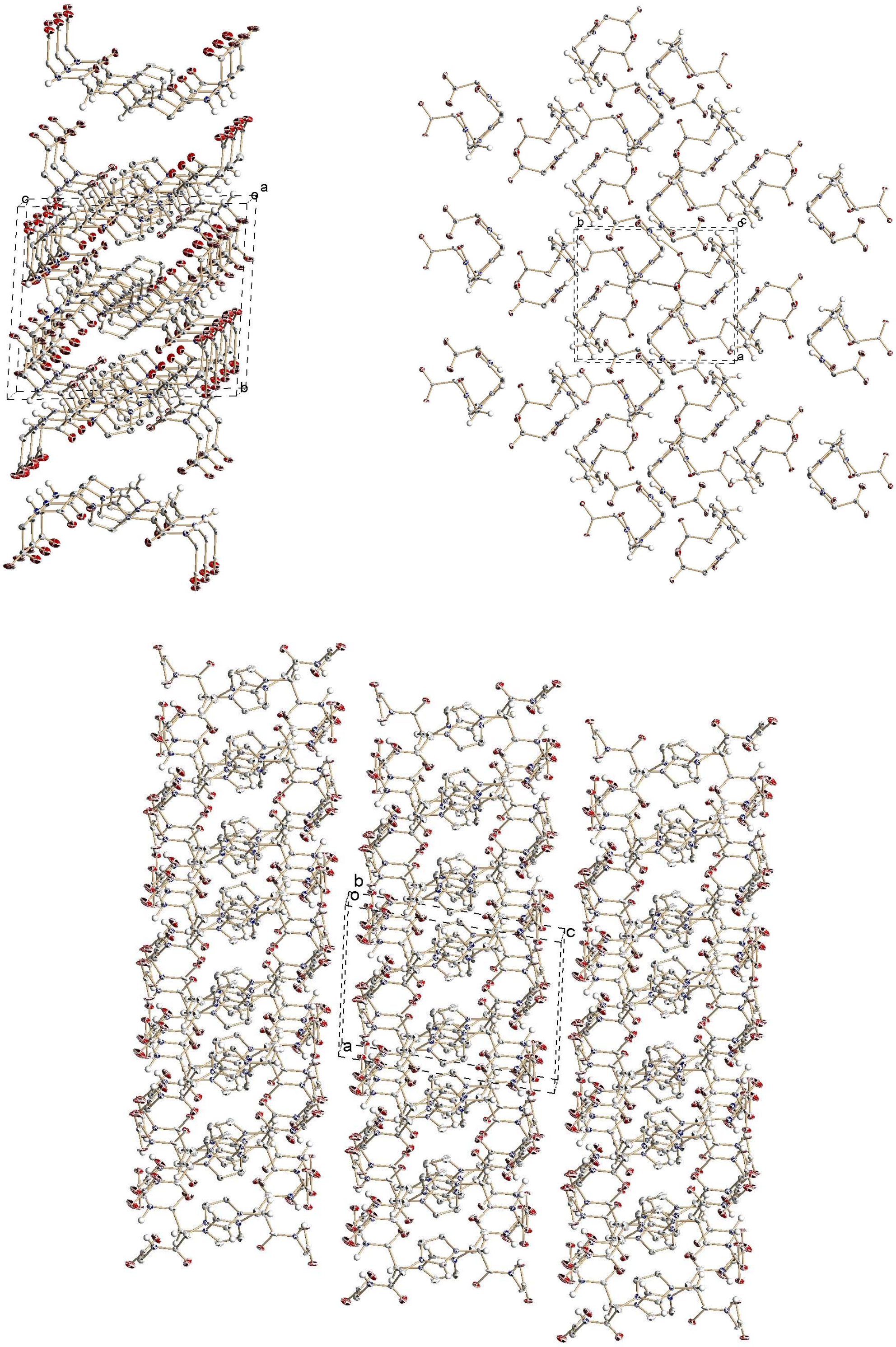

Figure S8. Crystal packing views of complex **2c**.

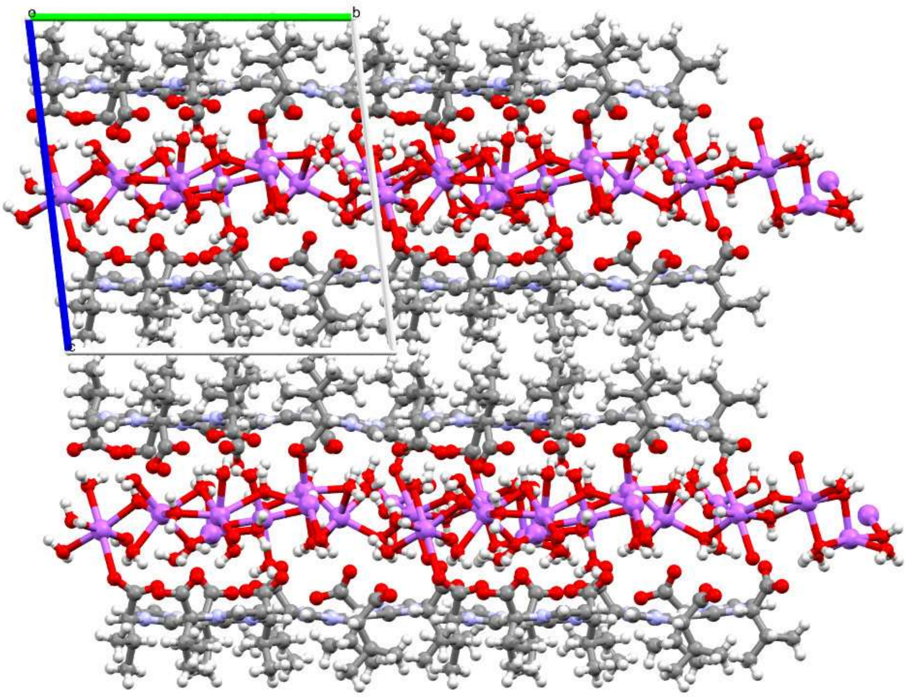

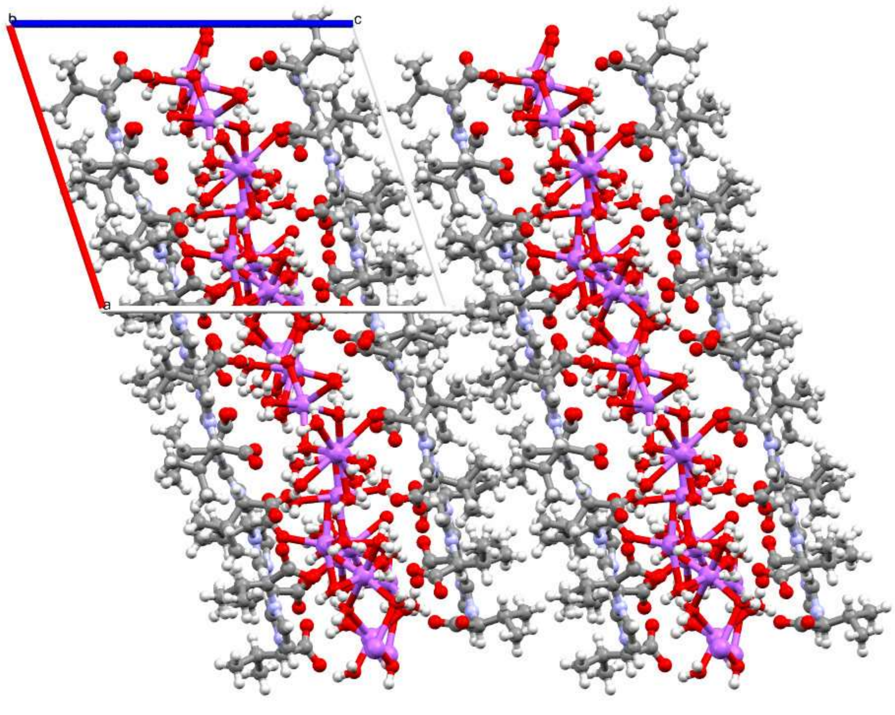

Figure S9. Crystal packing views of complex **2g** viewed along *b* axis.

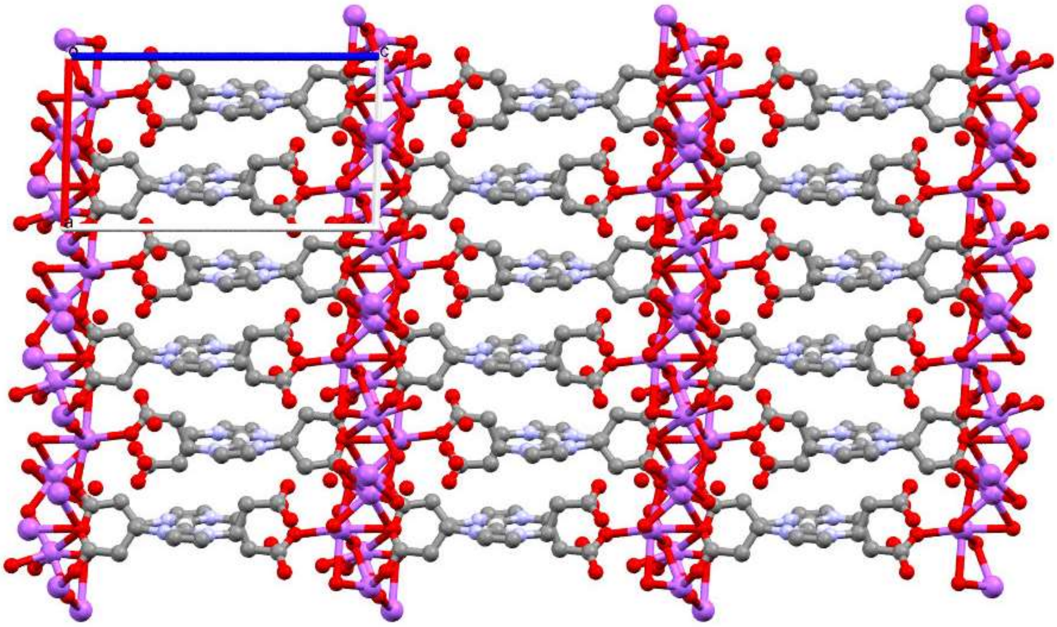

Table S4. Optimized structures of complexes $[Ni(CO)_3(NHC^R)]^{2-}$ and their calculated properties.

NHC	TEP (cm^{-1})	Unscaled ν_{CO} (A_1)	d(Ni-C) (Å)	Optimized structures[a]
NHC^{Gly}	2018.4	2115.5	2.008	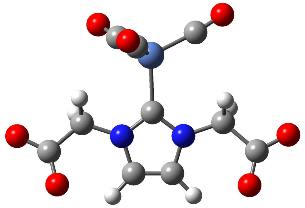
NHC^{Ala}	2019.4	2116.5	2.016	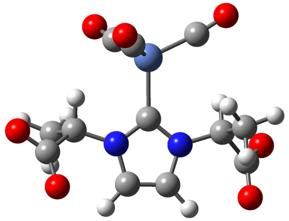
NHC^{Val}	2019.6	2116.8	2.027	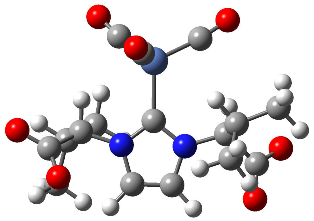
NHC^{iLeu}	2020.3	2117.5	2.026	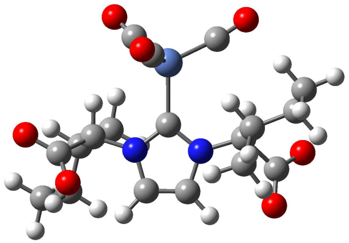

NHC^{Leu}	2021.5	2118.8	2.018	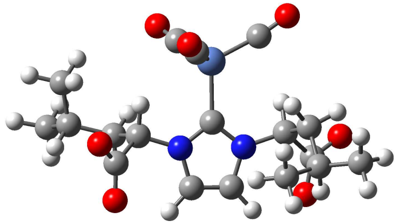
NHC$^{\beta\text{-}Me}$	2021.6	2118.9	2.015	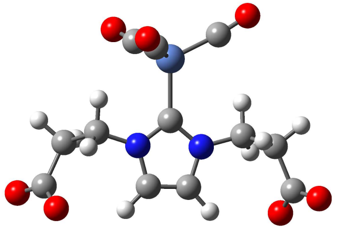
NHC^{GlyGly}	2039.6	2137.7	1.999	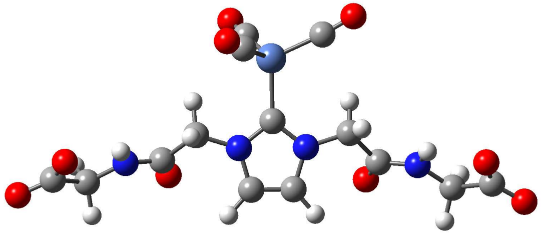
Methyl diester of NHC^{Gly}	2156.7	2057.7	1.985	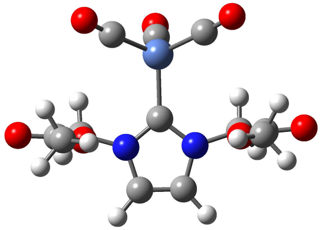

[a] The mPW1PW91 functional and the basis sets 6-311+G(2d) for Ni and 6-311+G(d,p) for all other atoms were used for calculations. Optimizations were carried out without symmetry restrictions, with tight option and the ultrafine integration grid.

Figure S10. Correlation between calculated TEP values (ν_{CO}) of the NHC^R carbenes and the MO energy of the σ lone pair orbitals.

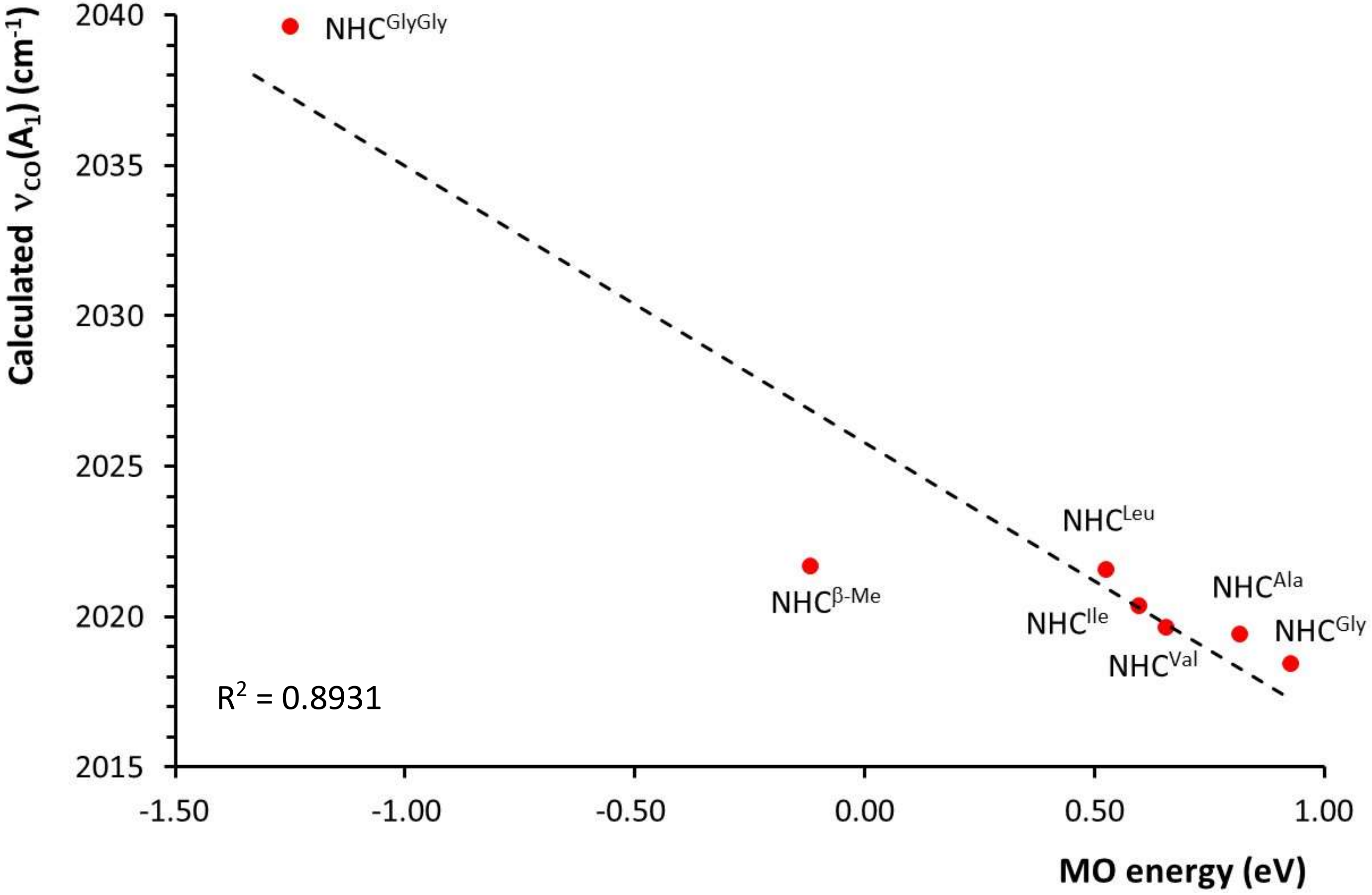

Table S5. Percent buried volume, $\%V_{\mathrm{bur}}$, of NHC^R ligands.[a]

**NHC^R**	**$\%V_{\mathrm{bur}}$**		
	Determined from optimized $[Ni(CO)_3(NHC^R)]^{2-}$	Determined from optimized $[Ag(NHC^R)_2]^{3-}$	Determined from X-ray data
NHC^{Gly}	26.2	26.3	28.9
NHC^{Ala}	28.1	29.0	-
NHC^{Val}	30.0	30.2	37.8
NHC^{iLeu}	29.9	30.4	-
NHC^{Leu}	28.9	28.6	-
$NHC^{\beta\text{-}Me}$	26.2	26.2	27.3
NHC^{GlyGly}	26.1	26.2	-

[a] Percentage buried volumes were calculated using the bond length for Ag−C fixed at 2 Å, a spherical radius of 3.5 Å, bond radii scaled by 1.17, and a mesh spacing of 0.10 (H atoms were excluded).

Figure S11. Selected steric maps from optimized $[Ag(NHC^R)_2]^{3-}$ complexes viewed along the Ag-$C_{carbene}$ vector.

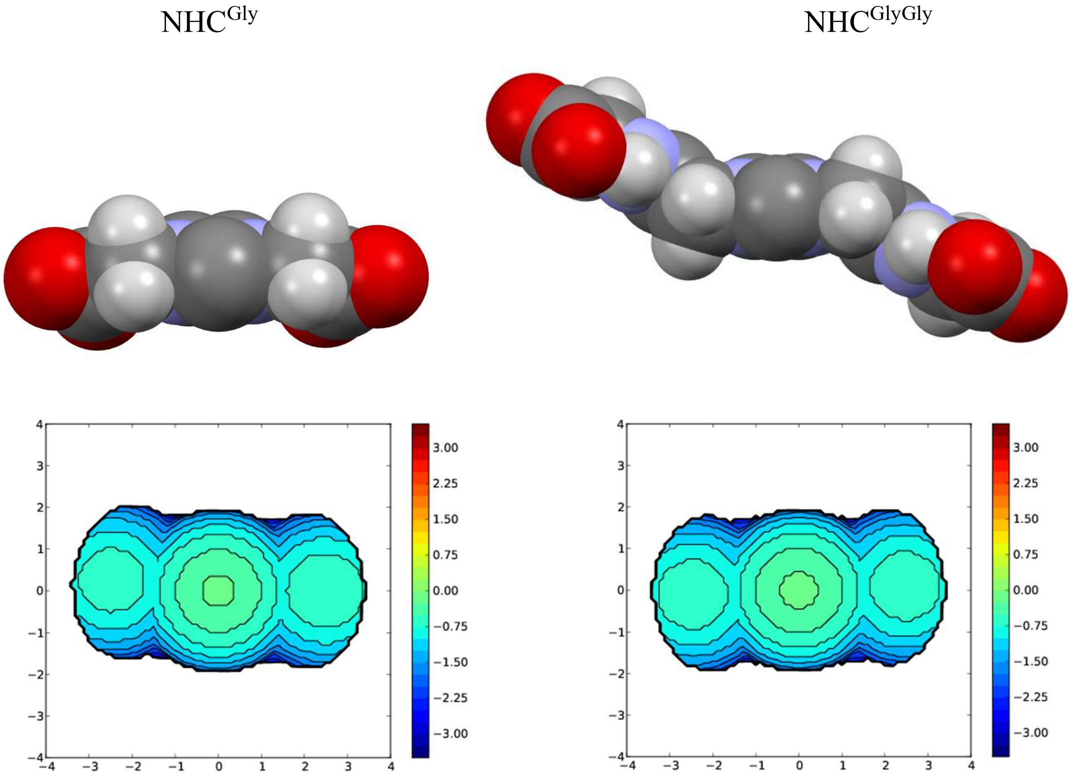

NHC^{Ala} NHC^{Val}

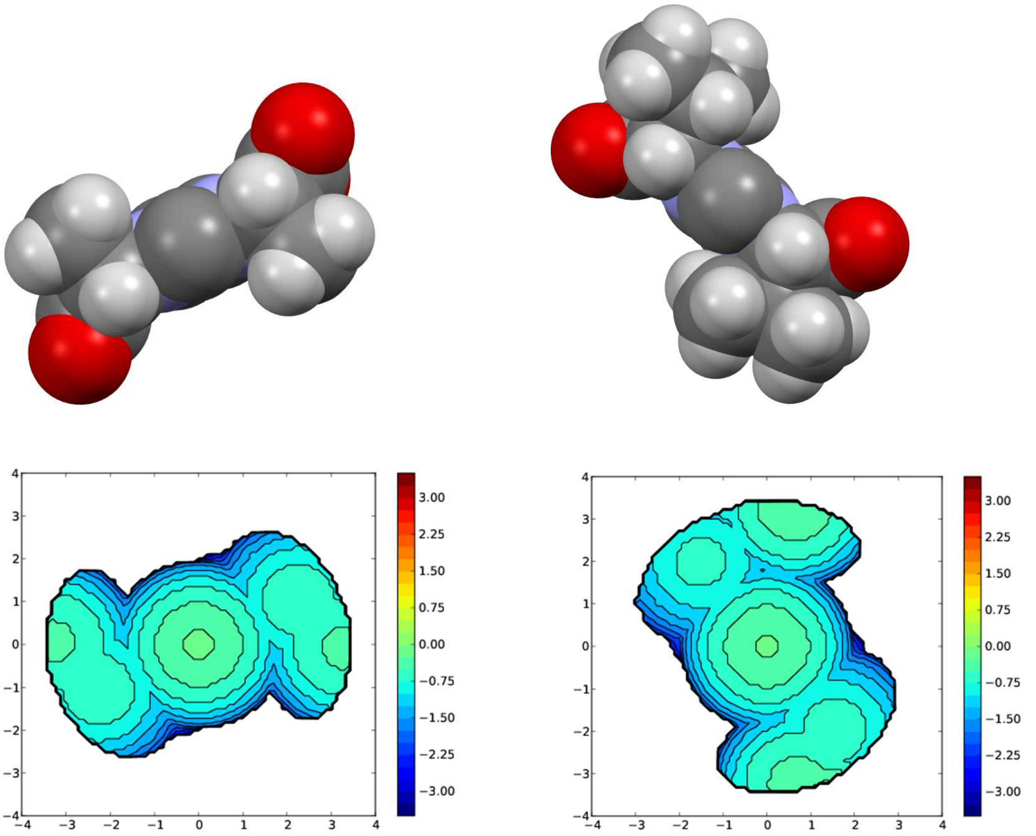

Figure S12. ^{1}H DOSY NMR spectra of **2b-c**.

2b

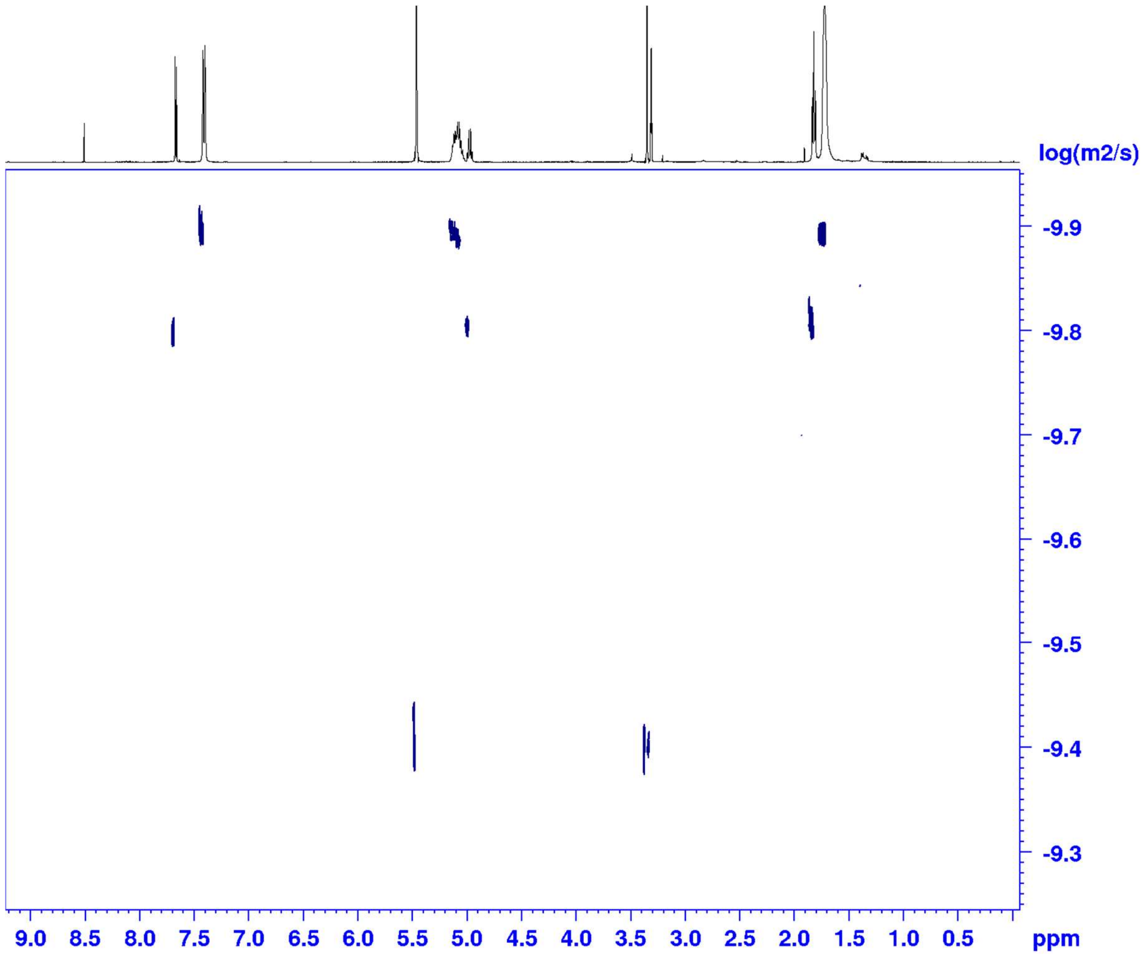

2c

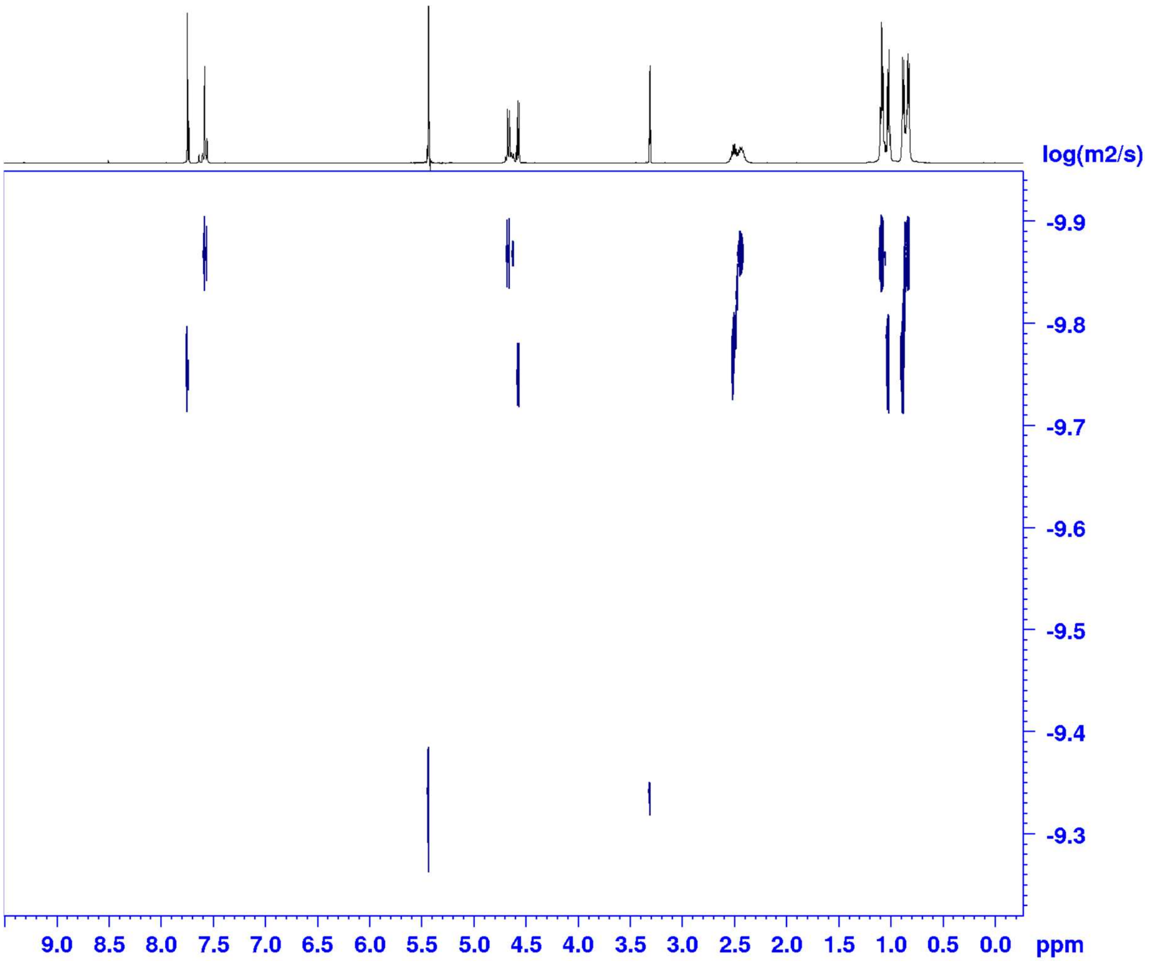
log(m2/s)
-9.9
-9.8
-9.7
-9.6
-9.5
-9.4
-9.3
9.0 8.5 8.0 7.5 7.0 6.5 6.0 5.5 5.0 4.5 4.0 3.5 3.0 2.5 2.0 1.5 1.0 0.5 0.0 ppm

Figure S13. Band selective constant time ^{1}H-^{13}C HMBC NMR spectra (resolution enhanced by NUS) of **2c**.

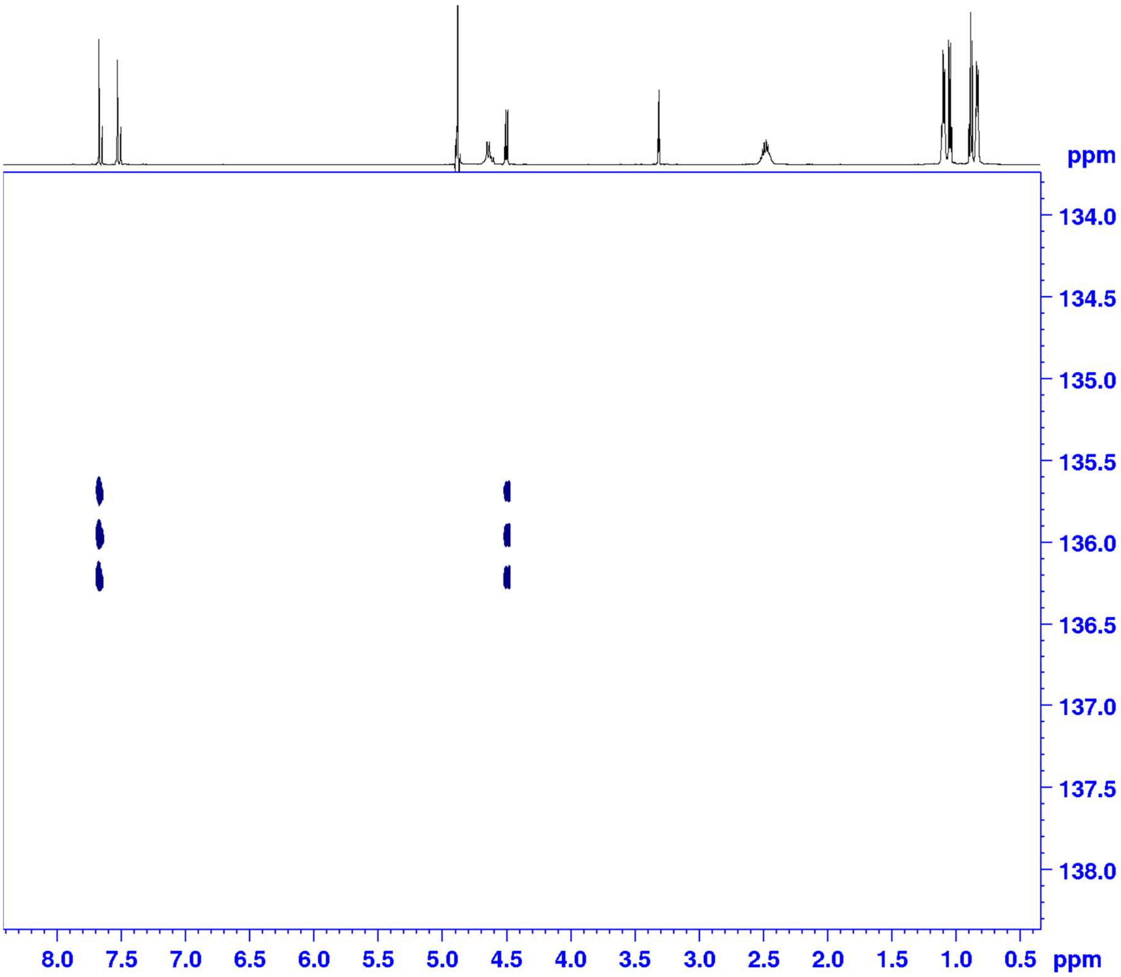

Figure S14. Correlation between calculated σ(^{109}Ag) and experimental δ(^{109}Ag) chemical shifts for selected NHC silver complexes.

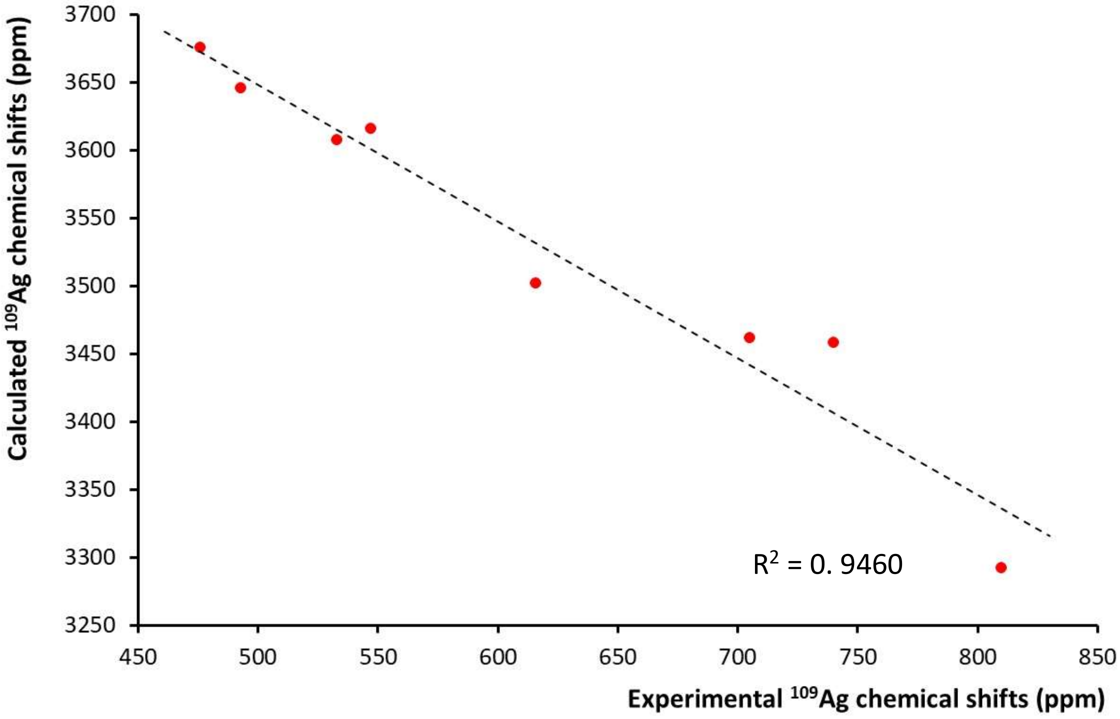

Table S6. Calculated σ(^{109}Ag) and experimental δ(^{109}Ag) data for selected NHC silver complexes.

**Complex**	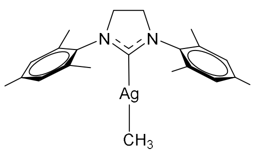	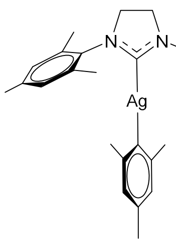	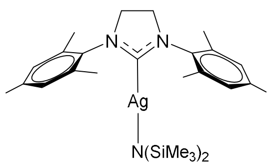	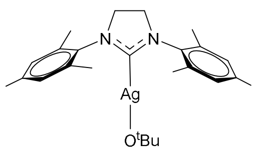	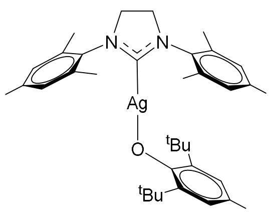
**δ_{exp}**	810	740	705	616	547
**σ_{calc}**	3292	3458	3462	3502	3616

**Complex**	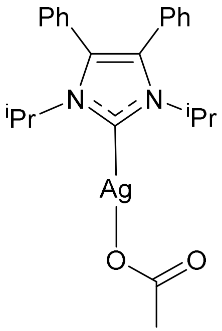	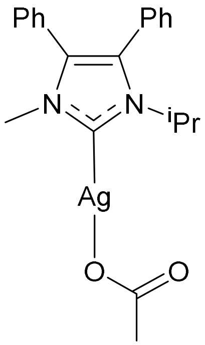	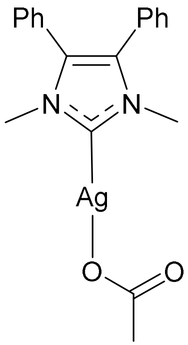
**δ_{exp}**	533	493	476
**σ_{calc}**	3608	3646	3676

Table S7. Antimicrobial activities of complexes **2** evaluated by MIC and MBC (μg/mL).

Complex	*E. coli*		*P. aeruginosa*		*S. Aureus*	
	**MIC**	**MBC**	**MIC**	**MBC**	**MIC**	**MBC**
**2a**	90.3	108.2	90.3	>216.4	90.3	144.4
**2b**	119.4	119.4	99.7	119.4	80.0	80.0
**2c**	189.3	212.7	189.3	212.7	165.2	212.7
**2c'**	141.8	141.8	141.8	141.8	141.8	165.2
**2e**	178.2	204.3	178.2	229.5	178.2	331.2
**2d**	204.3	229.5	229.5	229.5	204.3	229.5
**2f**	179.2	205.3	179.2	230.7	179.2	230.7
**2g**	139.1	159.4	119.4	179.1	119.4	277.6

Figure S15. Qualitative hemolysis test of complex **2a** and several control chemicals ($AgNO_3$, H_2O_2, sodium dodecyl sulfate, SDS and NaOH) for comparison.[a]

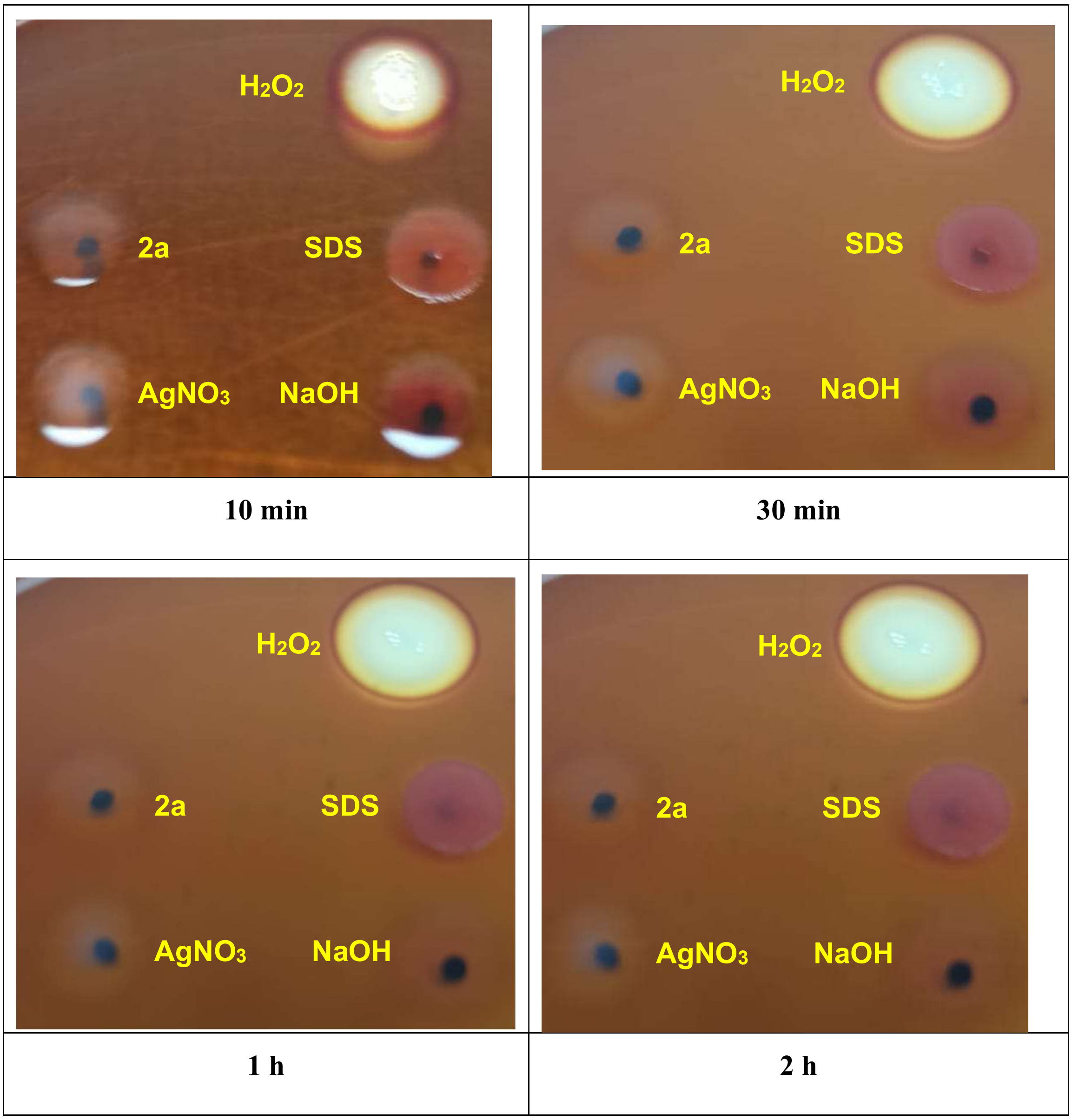

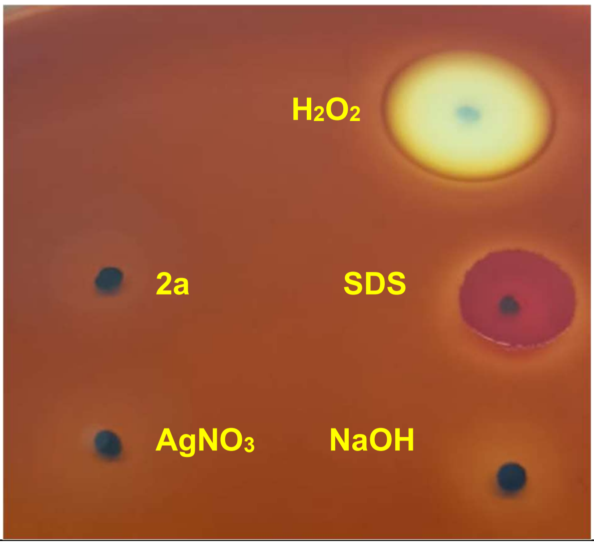
**18 h**

[a] Concentrations: **2a**, 10 mM; $AgNO_3$, 10 mM; H_2O_2, 10 %; SDS 1 %; and NaOH 0.2 M.

Table S8. Crystal data and structure refinement for **1f**.

Empirical formula	$C_{11}H_{14}N_4O_6$	
Formula weight	298.26	
Temperature	193(2) K	
Wavelength	0.71073 Å	
Crystal system	Triclinic	
Space group	$P\bar{1}$	
Unit cell dimensions	a = 9.0622(17) Å	α = 85.417(7)°
	b = 10.8523(14) Å	β = 82.590(10)°
	c = 13.124(2) Å	γ = 89.894(8)°
Volume	1275.8(4) $Å^3$	
Z	4	
Density (calculated)	1.553 Mg/m^3	
Absorption coefficient	0.128 mm^{-1}	
F(000)	624	
Crystal size	0.200 x 0.080 x 0.050 mm^3	
Theta range for data collection	2.266 to 25.249°.	
Index ranges	-10<=h<=10, -11<=k<=13, -15<=l<=15	
Reflections collected	37232	
Independent reflections	4586 [R(int) = 0.1559]	
Completeness to theta = 25.242°	99.6 %	
Absorption correction	Semi-empirical from equivalents	
Max. and min. transmission	0.7461 and 0.5847	
Refinement method	Full-matrix least-squares on F^2	
Data / restraints / parameters	4586 / 97 / 381	
Goodness-of-fit on F^2	1.162	
Final R indices [$I > 2\sigma(I)$]	$R1 = 0.1386$, $wR2 = 0.2881$	
R indices (all data)	$R1 = 0.1693$, $wR2 = 0.3012$	
Extinction coefficient	n/a	
Largest diff. peak and hole	0.973 and -0.515 e·$Å^{-3}$	

Table S9. Crystal data and structure refinement for **2c**.

Empirical formula	$C_{104}H_{224}Ag_4N_{16}Na_{12}O_{74}$	
Formula weight	3590.34	
Temperature	193(2) K	
Wavelength	0.71073 Å	
Crystal system	Triclinic	
Space group	P1	
Unit cell dimensions	a = 16.038(9) Å	α = 82.98(2)°
	b = 17.017(7) Å	β = 71.63(3)°
	c = 18.571(10) Å	γ = 88.37(2)°
Volume	4774(4) $Å^3$	
Z	1	
Density (calculated)	1.249 Mg/m^3	
Absorption coefficient	0.513 mm^{-1}	
F(000)	1872	
Crystal size	0.350 x 0.200 x 0.100 mm^3	
Theta range for data collection	1.811 to 25.250 °	
Index ranges	-19<=h<=19, -20<=k<=20, -22<=l<=22	
Reflections collected	170625	
Independent reflections	33843 [R(int) = 0.0867]	
Completeness to theta = 25.242°	98.7 %	
Absorption correction	Semi-empirical from equivalents	
Max. and min. transmission	0.7461 and 0.6494	
Refinement method	Full-matrix least-squares on F^2	
Data / restraints / parameters	33843 / 447 / 1891	
Goodness-of-fit on F^2	1.059	
Final R indices [I>2σ(I)]	$R1$ = 0.0708, w$R2$ = 0.1964	
R indices (all data)	$R1$ = 0.0887, w$R2$ = 0.2119	
Absolute structure parameter	0.034(6)	
Extinction coefficient	n/a	
Largest diff. peak and hole	2.769 and -0.975 e·$Å^{-3}$	

Table S10. Crystal data and structure refinement for **2g**.

Empirical formula	$C_{18}H_{38}AgN_4Na_3O_{17}$	
Formula weight	759.36	
Temperature	193(2) K	
Wavelength	0.71073 Å	
Crystal system	Triclinic	
Space group	$P\bar{1}$	
Unit cell dimensions	a = 8.6893(3) Å	α= 77.6730(10)°
	b = 11.9503(4) Å	β= 88.4700(10)°
	c = 15.6733(4) Å	γ = 75.6120(10)°
Volume	1539.52(8) $Å^3$	
Z	2	
Density (calculated)	1.638 mg/m^3	
Absorption coefficient	0.776 mm^{-1}	
F(000)	780	
Crystal size	0.500 x 0.400 x 0.200 mm^3	
Theta range for data collection	1.997 to 25.246°	
Index ranges	-10<=h<=10, -14<=k<=14, -17<=l<=18	
Reflections collected	31129	
Independent reflections	5521 [R(int) = 0.0426]	
Completeness to θ = 25.242°	98.9 %	
Absorption correction	Semi-empirical from equivalents	
Max. and min. transmission	0.7461 and 0.6266	
Refinement method	Full-matrix least-squares on F^2	
Data / restraints / parameters	5521 / 0 / 388	
Goodness-of-fit on F^2	1.087	
Final R indices [$I > 2\sigma(I)$]	$R1$ = 0.0335, w$R2$ = 0.0884	
R indices (all data)	$R1$ = 0.0341, w$R2$ = 0.0888	
Extinction coefficient	n/a	
Largest diff. peak and hole	0.671 and -0.761 e·$Å^{-3}$	

Table S11. Coordinates and optimized structures of NHC-silver complexes used for the correlation between calculated and experimental ^{109}Ag NMR chemical shifts.

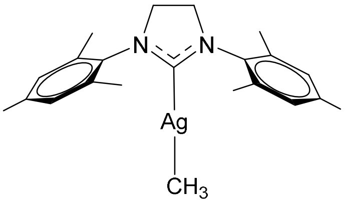

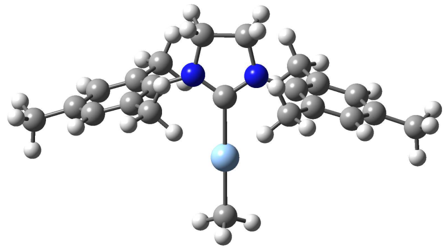

Ag	0.00007300	1.69883600	-0.00546000
N	1.08680900	-1.27546500	0.01150600
N	-1.08689100	-1.27544700	0.01146300
C	-0.00003500	-0.48907400	0.00532500
C	-0.76901400	-2.72394000	0.02223800
C	0.76890600	-2.72395100	0.02239200
C	2.44832100	-0.81673700	0.00490300
C	3.10150100	-0.62098000	-1.22115200
C	3.10806100	-0.60369700	1.22474400
C	4.43727300	-0.21775900	-1.20204700
C	4.44351500	-0.20083500	1.19275000
C	5.12628400	-0.00241100	-0.00801400
H	4.94964300	-0.05940100	-2.14719900
H	4.96099100	-0.02892900	2.13277300
C	-2.44839400	-0.81669000	0.00486300
C	-3.10819600	-0.60390200	1.22470600
C	-3.10150300	-0.62065700	-1.22119500
C	-4.44365000	-0.20101700	1.19272300
C	-4.43725900	-0.21740800	-1.20207800
C	-5.12634100	-0.00231400	-0.00803200
H	-4.96117800	-0.02931900	2.13275400
H	-4.94957200	-0.05882400	-2.14722400
C	2.37634800	-0.80035700	-2.53166600
H	3.04902100	-0.63284700	-3.37431300
H	1.95542600	-1.80448000	-2.64073200
H	1.54687600	-0.09338100	-2.61945800
C	6.55921900	0.47092000	-0.01455500
H	6.60965900	1.56500100	-0.00100800
H	7.10675300	0.11095100	0.85978400
H	7.09137400	0.13308200	-0.90687300
C	2.38970900	-0.76437600	2.54139600
H	1.56022700	-0.05667700	2.62312800
H	1.97010100	-1.76708200	2.66724300
H	3.06662600	-0.58416200	3.37801100
C	-2.37626900	-0.79975000	-2.53170300
H	-1.54681400	-0.09272900	-2.61930800
H	-1.95530800	-1.80383700	-2.64093900
H	-3.04890000	-0.63209600	-3.37435600
C	-6.55926500	0.47105000	-0.01459600
H	-7.10651200	0.11204400	0.86031400
H	-6.60967000	1.56514700	-0.00223400
H	-7.09173300	0.13226200	-0.90637100
C	-2.38993500	-0.76492700	2.54136400
H	-1.97060800	-1.76776800	2.66710700
H	-1.56026300	-0.05746700	2.62321700
H	-3.06683600	-0.58463600	3.37797500
H	1.19440600	-3.21371700	-0.85685500
H	1.19415300	-3.20044900	0.90901600
H	-1.19433900	-3.21357800	-0.85716700
H	-1.19445200	-3.20055100	0.90870600
C	0.00025600	3.82020600	-0.00953300

H	0.00153100	4.21571700	1.01184000
H	0.88455200	4.21512700	-0.52057400
H	-0.88519600	4.21530400	-0.51843100

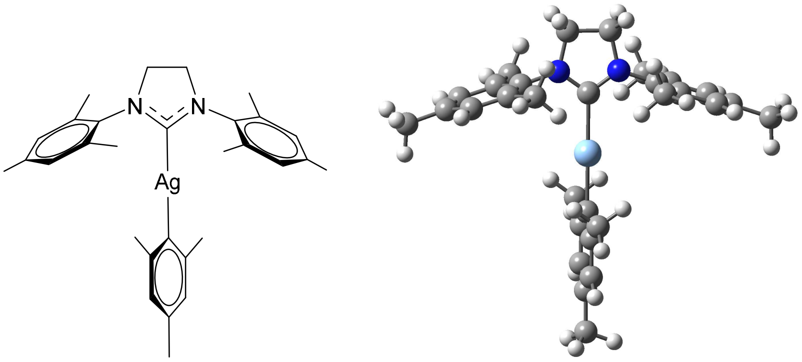

Ag	0.00131900	0.57341400	-0.01054300
N	1.08191200	-2.38556600	0.08088600
N	-1.09301600	-2.38035400	0.08151900
C	-0.00368300	-1.59911300	0.04214900
C	-0.77800400	-3.82768200	0.15249900
C	0.75998700	-3.83124200	0.15457600
C	2.44425900	-1.93071200	0.03331200
C	3.07925500	-1.79889000	-1.21073100
C	3.12224600	-1.65902600	1.23146600
C	4.41472800	-1.39425000	-1.23187400
C	4.45640500	-1.25753100	1.15878500
C	5.12070400	-1.11801300	-0.06074600
H	4.91301800	-1.28455600	-2.19122400
H	4.98826600	-1.03907700	2.08091200
C	-2.45311000	-1.91894500	0.03219400
C	-3.13326000	-1.65044300	1.22983200
C	-3.08391600	-1.77759100	-1.21294300
C	-4.46520200	-1.24198200	1.15548900
C	-4.41731500	-1.36628800	-1.23572700
C	-5.12528100	-1.09270200	-0.06517400
H	-4.99864700	-1.02578700	2.07723200
H	-4.91234100	-1.24922900	-2.19589600
C	2.33810500	-2.05093000	-2.50053000
H	2.99672500	-1.91563400	-3.35977300
H	1.93107300	-3.06528700	-2.55552700
H	1.49706100	-1.36148500	-2.61276200
C	6.55226900	-0.64363100	-0.11110300
H	6.60162600	0.45039800	-0.09722700
H	7.12675600	-1.00396700	0.74576900
H	7.05665800	-0.98007900	-1.01966500
C	2.42582800	-1.75879600	2.56589600
H	1.60197100	-1.04298300	2.63351100
H	2.00278700	-2.75250900	2.74147700
H	3.11848300	-1.54776200	3.38210100
C	-2.34046500	-2.02689500	-2.50193800
H	-1.49802100	-1.33850600	-2.61022300
H	-1.93498600	-3.04173300	-2.55910800
H	-2.99700800	-1.88779400	-3.36216500
C	-6.55432400	-0.61098100	-0.11722400
H	-7.13206500	-0.96963400	0.73814900
H	-6.59814400	0.48326500	-0.10191000
H	-7.05887300	-0.94355400	-1.02713600
C	-2.44152300	-1.76196800	2.56578300
H	-2.03330200	-2.76203000	2.74077500
H	-1.60776400	-1.05817700	2.63695100
H	-3.13324100	-1.54271900	3.38061100
H	1.18589100	-4.36000400	-0.70135600
H	1.18308600	-4.26890300	1.06188700

H	-1.20397800	-4.35211400	-0.70612300
H	-1.20557300	-4.26585400	1.05739500
C	0.00649500	2.70539800	-0.01799700
C	0.00834500	3.43693900	-1.22220100
C	0.00264500	3.43985700	1.18465300
C	0.00763500	4.83670300	-1.21299700
C	0.00195900	4.83946400	1.17252300
C	0.00714300	5.56058800	-0.02107100
H	0.00583100	5.37849700	-2.15804600
H	-0.00434300	5.38336100	2.11636700
C	0.00578000	2.72359600	-2.55966400
H	0.87939600	2.07115500	-2.66248100
H	-0.87992900	2.08863400	-2.66921800
H	0.01581400	3.42619000	-3.39848800
C	-0.00547400	2.73029700	2.52420600
H	-0.88766900	2.08964400	2.62887300
H	0.87192500	2.08426500	2.63589600
H	-0.00656200	3.43527700	3.36108300
C	0.04208500	7.07099000	-0.02222900
H	1.07082100	7.45102100	-0.01383300
H	-0.44506400	7.48485000	-0.90975100
H	-0.45983800	7.48650500	0.85631400

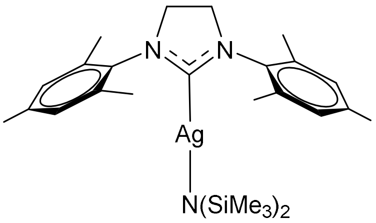

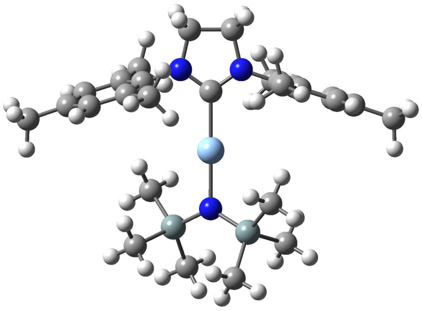

Ag	0.00005700	0.40005200	-0.00006600
N	1.08867500	-2.51472200	0.04340600
N	-1.08803100	-2.51491600	-0.04321600
C	0.00025300	-1.73201500	-0.00004200
C	-0.76500500	-3.96122900	-0.07110400
C	0.76587500	-3.96107800	0.07191300
C	2.44643400	-2.05436500	0.14553700
C	3.22243000	-1.95119200	-1.01937800
C	2.97883000	-1.74930500	1.40786500
C	4.54914400	-1.53708800	-0.89414800
C	4.31074600	-1.33957100	1.48072400
C	5.11231400	-1.22574200	0.34405000
H	5.15495900	-1.44501600	-1.79129600
H	4.73018000	-1.09645000	2.45311500
C	-2.44586500	-2.05481500	-0.14553800
C	-3.22208000	-1.95182100	1.01923700
C	-2.97810700	-1.74982600	-1.40796300
C	-4.54885300	-1.53793300	0.89377700
C	-4.31006700	-1.34031000	-1.48104400
C	-5.11185600	-1.22664300	-0.34449800
H	-5.15483200	-1.44596100	1.79082300
H	-4.72938000	-1.09724100	-2.45350200
C	2.64258400	-2.25072300	-2.37965500
H	3.35749600	-2.00795700	-3.16696200
H	2.37945800	-3.30766500	-2.49315700
H	1.73548400	-1.67019200	-2.56316100
C	6.53742600	-0.74113900	0.44766100
H	6.58409500	0.35054600	0.37397600
H	6.98938500	-1.02111700	1.40202100
H	7.16062400	-1.14728100	-0.35236100
C	2.13661300	-1.82498700	2.65726800

H	1.35852000	-1.05620800	2.65348500
H	1.63315400	-2.79027000	2.76205500
H	2.74839400	-1.67318000	3.54779600
C	-2.13567700	-1.82538700	-2.65722800
H	-1.35803100	-1.05615400	-2.65356200
H	-1.63162700	-2.79039800	-2.76164600
H	-2.74742500	-1.67419900	-3.54788500
C	-6.53702000	-0.74225100	-0.44838700
H	-7.15994300	-1.14719900	0.35244700
H	-6.58370700	0.34954400	-0.37637200
H	-6.98927500	-1.02370000	-1.40218000
C	-2.64253100	-2.25147500	2.37961400
H	-2.38144400	-3.30886700	2.49378100
H	-1.73431400	-1.67255100	2.56260200
H	-3.35685700	-2.00684900	3.16687600
H	1.27033600	-4.48242000	-0.74375400
H	1.10455300	-4.40265400	1.01299500
H	-1.10361000	-4.40324800	-1.01200500
H	-1.26938700	-4.48229400	0.74478500
N	-0.00031700	2.50066300	0.00013400
Si	1.35452500	3.25593500	-0.74281200
Si	-1.35550400	3.25529200	0.74308800
C	0.89763200	4.80408400	-1.75198700
H	0.44900700	5.59203800	-1.13939500
H	1.78297200	5.23670400	-2.23170300
H	0.18157100	4.56234800	-2.54413700
C	-0.89954200	4.80413100	1.75162700
H	-0.18314000	4.56324900	2.54372900
H	-0.45169800	5.59225900	1.13869300
H	-1.78515900	5.23617000	2.23136000
C	2.68100100	3.79153000	0.51137200
H	2.28493100	4.52400400	1.22193700
H	3.03713000	2.93663600	1.09623500
H	3.55252400	4.24461000	0.02474400
C	2.21533500	2.08062100	-1.96502400
H	2.59996300	1.18060400	-1.47355600
H	1.52858600	1.75941100	-2.75542800
H	3.06646400	2.57173500	-2.45075800
C	-2.68253000	3.78952000	-0.51110700
H	-2.28700700	4.52188500	-1.22209200
H	-3.03822400	2.93413400	-1.09552200
H	-3.55426500	4.24230600	-0.02458500
C	-2.21535700	2.07982200	1.96581800
H	-2.59966200	1.17952900	1.47460000
H	-1.52822300	1.75907800	2.75607500
H	-3.06660100	2.57058500	2.45169900

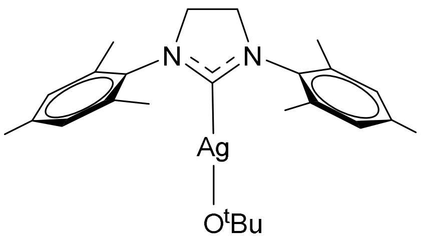

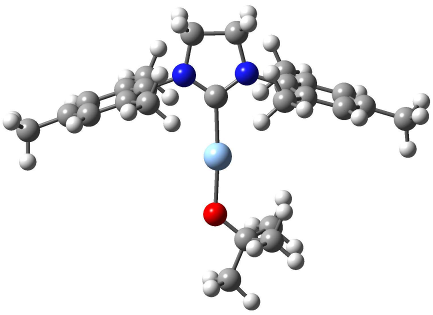

Ag	0.01642900	1.02904600	-0.00328800
N	1.48911700	-1.69359200	0.00161200
N	-0.67024000	-1.98302500	-0.00296400
C	0.30537300	-1.06291900	-0.00171600
C	-0.16081900	-3.37436000	0.01177400
C	1.36399500	-3.17025900	-0.00703600
C	2.77482400	-1.05179600	-0.00055300

C	3.39039900	-0.75008400	-1.22401500
C	3.39968000	-0.76480900	1.22325000
C	4.65579300	-0.16097900	-1.19873800
C	4.66374100	-0.17623700	1.19654100
C	5.30906900	0.13344200	-0.00211500
H	5.13778800	0.08283100	-2.14142900
H	5.15280800	0.05627000	2.13867200
C	-2.07985100	-1.70502000	0.00234200
C	-2.74870300	-1.56919600	1.22772600
C	-2.76443000	-1.60972500	-1.21915500
C	-4.12649000	-1.34516500	1.20653000
C	-4.14105500	-1.38565900	-1.18845300
C	-4.84089800	-1.25137500	0.01195400
H	-4.65273900	-1.23305500	2.15043400
H	-4.67940000	-1.30511400	-2.12893400
C	2.69541300	-1.00992600	-2.53758500
H	3.35038600	-0.77284600	-3.37735900
H	2.38405700	-2.05308600	-2.64599100
H	1.79607600	-0.39494000	-2.63252700
C	6.66003500	0.80534100	0.00171900
H	6.55825400	1.88527700	0.15240200
H	7.29636200	0.42577800	0.80518200
H	7.18702100	0.65672500	-0.94323200
C	2.71246300	-1.04117900	2.53753300
H	1.80773300	-0.43557900	2.64087300
H	2.41138400	-2.08813500	2.63895000
H	3.36867900	-0.80471400	3.37650800
C	-2.03422100	-1.70578400	-2.53568600
H	-1.30160900	-0.90049800	-2.63757600
H	-1.49131300	-2.64979900	-2.64321600
H	-2.73034100	-1.63054500	-3.37235800
C	-6.32415900	-0.97403800	0.01346600
H	-6.79259300	-1.29186000	0.94753700
H	-6.52150300	0.09695300	-0.10234800
H	-6.83034900	-1.48662800	-0.80826600
C	-2.00317300	-1.62201300	2.53814300
H	-1.44212600	-2.55333100	2.66112100
H	-1.28399300	-0.80162100	2.61317700
H	-2.69159600	-1.53930700	3.38044800
H	1.83726800	-3.58561900	-0.90007700
H	1.86185300	-3.59956900	0.86540400
H	-0.52766400	-3.92326400	-0.85841400
H	-0.50894800	-3.89572800	0.90676600
O	-0.11466800	3.06481700	-0.00369300
C	-1.28018100	3.83114200	-0.00612200
C	-0.84854500	5.31299300	-0.00885400
H	-1.70213000	6.00072200	-0.01092200
H	-0.23743400	5.51924900	0.87383800
H	-0.23610100	5.51560000	-0.89146600
C	-2.12950700	3.54907600	-1.26524300
H	-2.44723900	2.50038200	-1.27563600
H	-3.02723700	4.17653800	-1.32185800
H	-1.52888200	3.72564400	-2.16223800
C	-2.13109200	3.55419900	1.25306200
H	-2.44907500	2.50563400	1.26721300
H	-1.53150200	3.73422900	2.15005500
H	-3.02881100	4.18199200	1.30617000

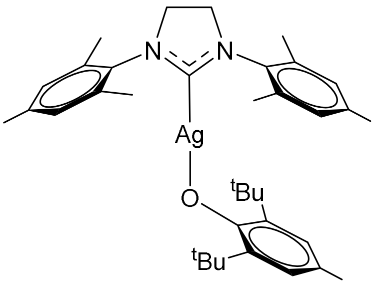

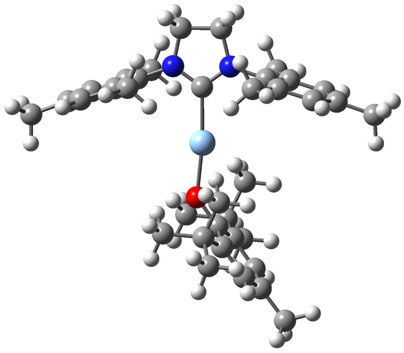

Ag	-0.32262400	-0.15206300	0.00339900
N	-3.35308300	0.42877500	0.00044000
N	-2.22534000	2.29538900	-0.00673800
C	-2.12320900	0.96038600	-0.00129400
C	-3.63045600	2.76854500	-0.00637300
C	-4.42636500	1.45111700	-0.00598800
C	-3.65071500	-0.97803600	0.00366000
C	-3.79945000	-1.65072100	-1.21858000
C	-3.80725600	-1.64318300	1.22968500
C	-4.11122200	-3.01082600	-1.18877200
C	-4.11910600	-3.00275500	1.20667000
C	-4.27346600	-3.70542500	0.01041600
H	-4.22052500	-3.54252700	-2.12982600
H	-4.23482000	-3.52853400	2.15039800
C	-1.12324200	3.21895200	-0.00818500
C	-0.60563700	3.67078000	1.21498600
C	-0.61195600	3.67389500	-1.23354500
C	0.43933000	4.59556000	1.18675100
C	0.43269500	4.59821100	-1.20881100
C	0.97337600	5.07034100	-0.01162800
H	0.85197600	4.94682300	2.12833900
H	0.84052800	4.95176000	-2.15174400
C	-3.59617600	-0.94323800	-2.53572400
H	-3.80035900	-1.61364500	-3.37163800
H	-4.24943300	-0.07239900	-2.64524800
H	-2.56752000	-0.58758200	-2.63993300
C	-4.56998000	-5.18458500	0.01632100
H	-3.64497600	-5.76654400	0.08677400
H	-5.19549000	-5.46659500	0.86658400
H	-5.08165300	-5.49676300	-0.89669800
C	-3.61137300	-0.92696600	2.54320600
H	-2.58339800	-0.57024300	2.65043200
H	-4.26548500	-0.05564800	2.64360400
H	-3.81972400	-1.59196400	3.38241400
C	-1.14540900	3.15909200	-2.54762300
H	-0.96585000	2.08581900	-2.65435100
H	-2.22338500	3.31652500	-2.64979400
H	-0.65978800	3.65971000	-3.38635200
C	2.13107400	6.03764800	-0.01532200
H	2.15813400	6.63785900	0.89681300
H	3.08443400	5.50284300	-0.08164400
H	2.08327300	6.72060300	-0.86686400
C	-1.13091500	3.15272800	2.53104600
H	-2.21121300	3.29284400	2.63274400
H	-0.93382900	2.08285700	2.64153700
H	-0.65306600	3.66392600	3.36786500
H	-5.05559100	1.33398500	-0.89116100
H	-5.06237700	1.33851800	0.87483500
H	-3.82349700	3.38219600	-0.88899400
H	-3.82253200	3.38201300	0.87671100
O	1.31572700	-1.43342300	0.00989200
C	2.64102000	-1.34239600	0.00357900
C	3.36784000	-1.34889000	-1.23343300
C	3.37787400	-1.32861900	1.23447800
C	4.76075200	-1.25585600	-1.19556400

C	4.77046500	-1.23620400	1.18374700
C	5.48568300	-1.18026400	-0.00935700
H	5.32011800	-1.25019200	-2.12418000
H	5.33733800	-1.21499600	2.10751300
C	2.66691300	-1.43442900	2.60546800
C	2.64577700	-1.47691000	-2.59672500
C	1.72667000	-2.72096800	-2.61010400
H	1.19421000	-2.79263800	-3.56632400
H	2.32142400	-3.63212800	-2.49204100
H	1.00436000	-2.68412100	-1.79840500
C	1.82534700	-0.20169400	-2.89356300
H	1.07483800	-0.01603200	-2.12433200
H	2.47844500	0.67490200	-2.94074100
H	1.30684100	-0.28848200	-3.85602800
C	3.62841700	-1.64400300	-3.77470900
H	3.06208000	-1.77773600	-4.70165600
H	4.27204500	-0.77069700	-3.91254700
H	4.26850700	-2.52180700	-3.65247300
C	1.84741600	-0.15525100	2.88758300
H	2.49965000	0.72284300	2.91441200
H	1.09032200	0.01639800	2.12160600
H	1.33710700	-0.22636400	3.85569000
C	1.74917500	-2.67878200	2.64708700
H	1.01917600	-2.65462200	1.84181900
H	2.34341000	-3.59130200	2.53730400
H	1.22589200	-2.73596900	3.60933100
C	3.65939700	-1.58045000	3.77811500
H	4.30366900	-0.70459500	3.89491800
H	3.10079900	-1.69792400	4.71193500
H	4.29893200	-2.46007900	3.66631600
C	6.98991400	-1.03712600	-0.01675500
H	7.44239000	-1.48933600	0.87098400
H	7.43681400	-1.51799500	-0.89223600
H	7.30503900	0.01414800	-0.03489400

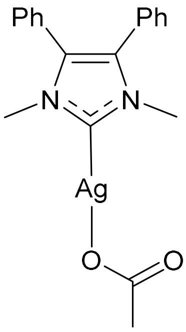

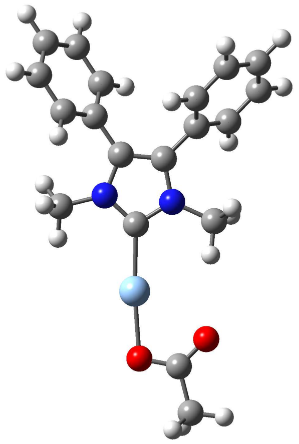

Ag	2.83995200	-0.67552000	-0.08920300
N	-0.27739200	-1.34675400	-0.08829900
N	0.11773900	0.76595000	-0.02919300
C	0.72990700	-0.44155500	-0.07987500
C	-1.27204900	0.62790600	-0.00832100
C	-1.52513100	-0.71843900	-0.04107000
O	4.98455700	-0.72105300	-0.07498900
C	0.84436600	2.03653000	0.05009400
H	1.89576000	1.83094900	0.24496800
H	0.76278700	2.58564100	-0.88832500
H	0.43366500	2.64460500	0.85589600
C	-0.05744900	-2.78619200	-0.18924900
H	0.96757500	-2.94580600	-0.51521900
H	-0.20327100	-3.27606100	0.77470300

H	-0.74415700	-3.21895500	-0.91625700
C	5.33085700	0.50189700	0.13653700
C	6.83577100	0.73942700	0.19079800
H	7.29479500	0.42476800	-0.75005600
H	7.05661500	1.79058200	0.37300600
H	7.27905200	0.12809000	0.98113900
O	4.54059900	1.44571000	0.28998900
C	-2.21712900	1.75820000	0.06513100
C	-2.21109600	2.77318200	-0.90248500
C	-3.15647300	1.82263000	1.10320900
C	-3.11702300	3.82700600	-0.82929300
H	-1.50748700	2.72705900	-1.72677200
C	-4.06494400	2.87475300	1.17158100
H	-3.16803200	1.04570000	1.85951900
C	-4.04626100	3.88090500	0.20796100
H	-3.10065000	4.60344600	-1.58706400
H	-4.78459800	2.91093200	1.98282400
H	-4.75235100	4.70282300	0.26419800
C	-2.81403100	-1.43444900	-0.05656900
C	-3.11364300	-2.40904500	0.90657700
C	-3.77449800	-1.13535700	-1.03271400
C	-4.33817100	-3.07022500	0.88930900
H	-2.39373300	-2.63421300	1.68639600
C	-5.00063100	-1.79362800	-1.04461200
H	-3.55343400	-0.38588600	-1.78436300
C	-5.28488900	-2.76472000	-0.08659400
H	-4.55586600	-3.81880200	1.64408000
H	-5.73303900	-1.55057300	-1.80737200
H	-6.23997100	-3.27943300	-0.09880200

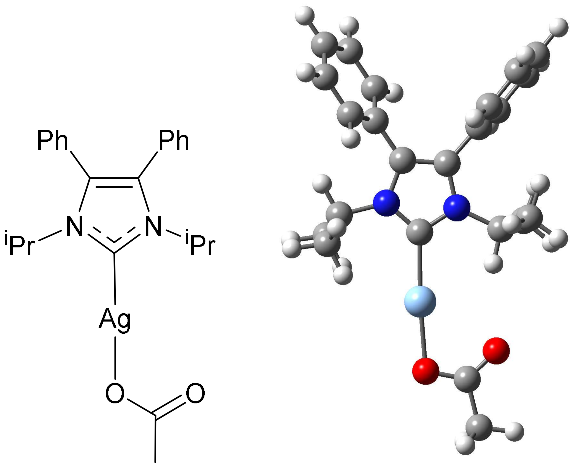

Ag	-2.77739000	-0.59981300	0.02029000
N	0.33826800	-1.31655400	0.03961300
N	-0.03825000	0.81109300	-0.00306500
C	-0.65517000	-0.39796500	0.01819300
C	1.35321600	0.65491000	0.01386500
C	1.59057500	-0.69194200	0.03453800
O	-4.91959900	-0.65067700	0.01589200
C	-0.82324100	2.07905900	-0.08311900
H	-1.85502900	1.73873700	-0.18060700
C	0.12508700	-2.78318300	0.15070900
H	1.12896400	-3.20782100	0.16523100
C	-5.29190100	0.58091300	-0.06600200
C	-6.80246000	0.78892900	-0.08349000
H	-7.24562300	0.36530700	0.82152000
H	-7.04821600	1.84820600	-0.15072400
H	-7.23992200	0.25571800	-0.93157700
O	-4.52324300	1.55142400	-0.12823200
C	2.36834000	1.73669400	-0.02248200

C	2.72082000	2.43827500	1.13722000
C	3.04197800	2.02799800	-1.21510400
C	3.70719100	3.42082500	1.10071600
H	2.22501800	2.20634800	2.07365300
C	4.03003100	3.00849100	-1.25152300
H	2.78544900	1.48321100	-2.11733800
C	4.36187500	3.71021600	-0.09465600
H	3.96809900	3.95550700	2.00823700
H	4.54096100	3.22315400	-2.18441700
H	5.13109900	4.47502800	-0.12291200
C	2.88642400	-1.40202100	0.02113500
C	3.24386600	-2.22681400	-1.05582500
C	3.79505400	-1.24239100	1.07480600
C	4.47290400	-2.87927200	-1.07322700
H	2.56022500	-2.34381600	-1.89071600
C	5.02754500	-1.89016600	1.05306100
H	3.53095000	-0.60797900	1.91343400
C	5.36845100	-2.71234900	-0.01826100
H	4.73470700	-3.51147700	-1.91543800
H	5.72060100	-1.75386500	1.87678800
H	6.32807700	-3.21855000	-0.03330800
C	-0.61234900	-3.33436800	-1.07093600
H	-1.63482100	-2.95572500	-1.12592700
H	-0.10101000	-3.06477700	-1.99824000
H	-0.65843200	-4.42481400	-1.01272000
C	-0.55899600	-3.14380800	1.47137000
H	-0.00636600	-2.73976200	2.32280400
H	-1.57972300	-2.75886600	1.51156800
H	-0.60153900	-4.23073400	1.57921000
C	-0.73904000	2.89879600	1.20517300
H	-0.93974000	2.27391700	2.07856100
H	0.23020800	3.38368100	1.32998000
H	-1.50492600	3.67705800	1.17619000
C	-0.48343500	2.88657000	-1.33608500
H	0.51356900	3.32766400	-1.29474400
H	-0.55546800	2.26571500	-2.23231600
H	-1.20775900	3.69801700	-1.43739500

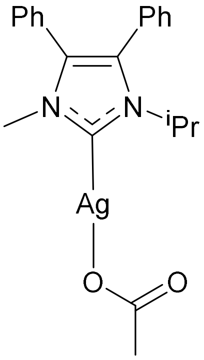

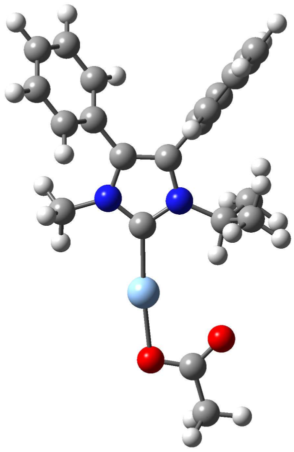

Ag	2.79602500	-0.89705400	-0.04875000
N	-0.32166200	-1.51243500	-0.06564300
N	0.09220100	0.60451000	-0.00027600
C	0.68926300	-0.61407100	-0.03838600
C	-1.30240900	0.47030800	-0.01260900
C	-1.56218000	-0.87295600	-0.04720200
O	4.93624500	-0.99584500	-0.05473200
C	0.90396100	1.85490400	0.09093000
H	1.92916600	1.49105500	0.17487600
C	5.33044400	0.22776600	0.03854000

C	6.84439100	0.40962700	0.04989700
H	7.27642200	-0.01746300	-0.85880700
H	7.10829900	1.46427700	0.12114000
H	7.27628200	-0.13477100	0.89375900
O	4.57969900	1.21155500	0.11610600
C	-2.29971000	1.56751700	0.03771900
C	-2.63913500	2.29243200	-1.11144400
C	-2.97075800	1.84944000	1.23402500
C	-3.61096700	3.28864100	-1.06068600
H	-2.14531200	2.06749700	-2.05058600
C	-3.94416200	2.84385300	1.28441900
H	-2.72411800	1.28618700	2.12763200
C	-4.26343400	3.56851900	0.13823500
H	-3.86244300	3.84151700	-1.95989700
H	-4.45355400	3.05127300	2.21975500
H	-5.02128000	4.34410700	0.17743000
C	-2.85504700	-1.58512800	-0.05468700
C	-3.18728000	-2.47836500	0.97411200
C	-3.78096200	-1.37012600	-1.08367400
C	-4.41001600	-3.14255500	0.96985600
H	-2.49321500	-2.63631500	1.79322500
C	-5.00621400	-2.03135900	-1.08384400
H	-3.53522500	-0.68385100	-1.88617800
C	-5.32274100	-2.92126700	-0.05993000
H	-4.65344700	-3.82704700	1.77590300
H	-5.71264800	-1.85296000	-1.88791000
H	-6.27694500	-3.43780500	-0.06217900
C	0.82680100	2.69535400	-1.18428400
H	1.00666300	2.07939500	-2.06845800
H	-0.13222900	3.20375400	-1.29387900
H	1.61009000	3.45586800	-1.15010100
C	0.58890700	2.64888500	1.35878800
H	-0.40169700	3.10560100	1.33330000
H	0.65952100	2.01331700	2.24471100
H	1.32636500	3.44766400	1.46552600
C	-0.11847900	-2.95477800	-0.15629900
H	0.92503300	-3.13077400	-0.40671200
H	-0.34490800	-3.44443700	0.79188100
H	-0.75696800	-3.37461100	-0.93359400

Table S12. Coordinates of complexes $[Ni(CO)_3(NHC^R)]^{2-}$ used for the determination of the Tolman Electronic Parameter (TEP).

$[Ni(CO)_3(NHC^{Gly})]^{2-}$

Ni	-0.03544600	1.64388100	0.06741900
C	-0.52967200	1.94526200	1.75782600
C	1.53055500	2.44085100	-0.27517900
C	-1.18390600	2.26711900	-1.15458200
O	-0.82239000	2.14242500	2.85034200
O	-1.86697500	2.71641400	-1.96113800
O	2.46882500	3.06208000	-0.50419700
C	0.72224400	-2.48298000	-0.39372600
C	-0.63071100	-2.49123500	-0.41967200
H	1.46488100	-3.26138900	-0.41134000
H	-1.35608000	-3.28424100	-0.45700900
C	0.02648500	-0.35434000	-0.11651700
N	1.10922800	-1.17210900	-0.19687100
N	-1.04135200	-1.18635500	-0.23851000
C	2.49645600	-0.69850500	-0.17638300
H	2.70821000	-0.20341800	-1.12653300
H	2.56804000	0.06315000	0.59876400
C	-2.43274800	-0.73649000	-0.29944200
H	-2.51873400	0.13825000	0.34248800
H	-2.64858600	-0.40299200	-1.31814300
C	3.67068700	-1.70510200	0.07145800
C	-3.58343700	-1.72158000	0.09603000
O	4.77485000	-1.13348700	-0.02107100
O	3.41946700	-2.89829600	0.32516400
O	-3.30005800	-2.87577400	0.46614100
O	-4.70073400	-1.18170900	-0.03113700

$[Ni(CO)_3(NHC^{Ala})]^{2-}$

Ni	0.08663200	1.68397600	-0.00067600
C	1.06864900	2.07594500	-1.44739200
C	-1.47149300	2.54025100	-0.19985800
C	0.80677200	2.21160800	1.54828300
O	1.63908900	2.36852900	-2.39825500
O	1.22755800	2.59543600	2.54593200
O	-2.39364400	3.21025100	-0.34433000
C	-0.80424000	-2.45542000	-0.07346700
C	0.54225200	-2.50729000	0.03422100
H	-1.57143800	-3.20711800	-0.15252400
H	1.24527300	-3.32007900	0.00722100
C	-0.05116200	-0.32746200	0.03600100
N	-1.15299300	-1.11847100	-0.06209000
N	0.98906500	-1.20299000	0.09565100
C	-2.53067900	-0.62716600	-0.21376100
H	-2.56412700	0.32290800	0.31231800
C	2.38121600	-0.82646300	0.36625900
H	2.45871500	0.22526400	0.09932300
C	-3.61799400	-1.55443100	0.44242800
C	3.48764000	-1.59158800	-0.44584900
O	-3.68022200	-2.72527700	0.00482300
O	-4.34112700	-0.99333000	1.28637300
O	3.21598500	-2.72220500	-0.89421700
O	4.56508200	-0.96368300	-0.48460800
C	-2.84917800	-0.42819700	-1.69121500
H	-2.11659100	0.23349800	-2.16198900
H	-2.84472900	-1.39460800	-2.19989400
H	-3.84468400	0.01164600	-1.79757300
C	2.67653100	-0.98442400	1.85786400

H	3.69375500	-0.64257600	2.05497100
H	1.97454900	-0.40477400	2.46343800
H	2.59941700	-2.03520300	2.15188000

$[Ni(CO)_3(NHC^{Val})]^{2-}$

Ni	0.02048500	1.84045800	-0.02349800
C	-0.00580600	2.18064400	1.73544600
C	1.49399000	2.52379700	-0.77741100
C	-1.32324300	2.68237100	-0.85048300
O	-0.00760900	2.41848600	2.85685800
O	-2.11045400	3.34058400	-1.36848900
O	2.36981700	3.10131900	-1.24460300
C	0.49684200	-2.36751500	0.02848800
C	-0.84149800	-2.29128500	0.19418200
H	1.19750600	-3.18485000	0.00467800
H	-1.57333500	-3.03648800	0.44162600
C	-0.08176100	-0.18277900	-0.09020500
N	0.94851300	-1.07329900	-0.14627200
N	-1.18545900	-0.95796800	0.10825900
C	2.36317600	-0.71174000	-0.32328900
H	2.35961000	0.17563400	-0.95157100
C	-2.56544400	-0.47509800	0.12667800
H	-2.48589900	0.60780600	0.20383000
C	3.22070200	-1.78196800	-1.09444100
C	-3.43292500	-0.95136500	1.34855700
O	3.20559800	-2.95315400	-0.65209200
O	3.88274100	-1.31578200	-2.04023500
O	-3.01961500	-1.91239200	2.02179600
O	-4.49450900	-0.30278200	1.45715500
C	3.00394500	-0.32990500	1.02923500
H	2.40487500	0.50663300	1.41058400
C	-3.33012000	-0.79340900	-1.18292500
H	-4.24151500	-0.19574100	-1.08559300
C	-3.76104500	-2.25519600	-1.31029100
H	-2.90247400	-2.91067100	-1.48197400
H	-4.27951600	-2.59511700	-0.41160000
H	-4.43840600	-2.37184500	-2.16422500
C	-2.57746200	-0.35469700	-2.43558200
H	-3.18579900	-0.53728900	-3.32913400
H	-2.32641500	0.70642300	-2.40879900
H	-1.64202400	-0.90971900	-2.54977300
C	4.43103900	0.17096300	0.81633900
H	5.07320500	-0.63088600	0.44270600
H	4.46457900	0.98064600	0.08334300
H	4.84905500	0.53991100	1.75963300
C	2.96589900	-1.44551200	2.07060200
H	3.51077500	-2.32202700	1.71346300
H	3.41475300	-1.09677000	3.00792100
H	1.94057600	-1.75367800	2.28639800

$[Ni(CO)_3(NHC^{iLeu})]^{2-}$

Ni	0.28990800	1.85492000	-0.26055500
C	0.49030400	2.39327100	1.43739400
C	1.76675500	2.21675300	-1.20584800
C	-0.99534000	2.78947900	-1.08026700
O	0.64091700	2.75492800	2.51440700
O	-1.72968600	3.49237200	-1.61690000
O	2.66934200	2.59887400	-1.80445400
C	0.15430800	-2.34705900	0.25628900
C	-1.12537300	-2.04052600	0.56044900
H	0.71886100	-3.26404200	0.25401700
H	-1.91988600	-2.63282000	0.97266200
C	-0.10877800	-0.12159000	-0.06114800

N	0.76428200	-1.16655100	-0.12312900
N	-1.27857600	-0.68565700	0.35254300
C	2.18050200	-1.05551600	-0.50260700
H	2.22198400	-0.25275700	-1.23484900
C	-2.55873100	0.00990000	0.47199900
H	-2.31633300	1.06717500	0.37876800
C	2.76597900	-2.32614300	-1.22290800
C	-3.29415300	-0.16388500	1.85087100
O	2.64077200	-3.42473200	-0.63562000
O	3.35717200	-2.07657800	-2.28984300
O	-2.91519000	-1.07797700	2.60473800
O	-4.23523200	0.64485600	1.99149700
C	3.04404700	-0.63422700	0.70804500
H	2.63684400	0.33219500	1.03554200
C	-3.54430700	-0.34233500	-0.67126800
H	-4.34098700	0.39802800	-0.54128800
C	-4.19448600	-1.72591400	-0.52340400
H	-3.48510700	-2.49923500	-0.83947800
H	-4.40714000	-1.90716100	0.53363300
C	-2.91878300	-0.16878400	-2.05293000
H	-3.65073000	-0.35496500	-2.84563300
H	-2.53095600	0.84101000	-2.19245400
H	-2.08519900	-0.86217100	-2.19618800
C	4.48967800	-0.40091100	0.25418700
H	4.94822000	-1.36609000	0.01522200
H	4.47676400	0.15675000	-0.68704900
C	2.95629300	-1.60464500	1.88381100
H	3.31055000	-2.59454700	1.58774900
H	3.54820000	-1.24376500	2.73095400
H	1.92547400	-1.70867600	2.22880300
C	5.34647900	0.34892400	1.26926300
H	5.45764800	-0.20787200	2.20412000
H	6.35206300	0.53110900	0.87564500
H	4.90605600	1.32072200	1.51517000
C	-5.49028900	-1.87374800	-1.31598400
H	-5.33513800	-1.74214300	-2.39123300
H	-5.93256000	-2.86470900	-1.16919000
H	-6.22538800	-1.13058000	-0.99155700

$[Ni(CO)_3(NHC^{Leu})]^{2-}$

Ni	-0.10104400	1.84628300	0.23126300
C	-1.34610900	2.49031900	-0.87602500
C	-0.52662300	1.92611000	1.97277400
C	1.34498800	2.88311500	0.03260300
O	-2.09772000	2.96500200	-1.60431700
O	2.18303000	3.66522800	-0.03697400
O	-0.73536900	2.03872100	3.09385300
C	-0.25960600	-2.31138300	-0.47734500
C	1.02077400	-2.09574400	-0.85264200
H	-0.86725700	-3.19671600	-0.40105500
H	1.78878700	-2.72863100	-1.26159600
C	0.15965000	-0.10553300	-0.21019100
N	-0.77034600	-1.09045100	-0.08231500
N	1.26463300	-0.74565600	-0.68596500
C	-2.17314100	-0.88793000	0.30126000
H	-2.20161300	0.07458300	0.80552200
C	2.55844700	-0.08375000	-0.91853600
H	2.30828900	0.91858200	-1.25907700
C	-2.73534300	-1.97170100	1.28914300
C	3.49012400	-0.68847300	-2.03414200
O	-2.63214100	-3.16198300	0.92116400
O	-3.29833600	-1.50709000	2.29889400
O	3.40414700	-1.90503000	-2.30160100
O	4.28481300	0.15677900	-2.48837400
C	-3.03765800	-0.85581200	-0.96482400
H	-2.68617200	-0.04256800	-1.61285800
C	3.37452200	0.06153600	0.37497500

H	4.22279700	0.69356000	0.09779600
C	3.89302400	-1.23992800	1.01297100
H	4.01585800	-1.97841300	0.21281300
C	-4.54151700	-0.70487500	-0.72013400
H	-4.85962600	-1.55992000	-0.11308300
C	-5.29223100	-0.75801600	-2.04973200
H	-5.07227800	-1.68224100	-2.59280700
H	-6.37589400	-0.70610100	-1.89610100
H	-5.00649200	0.08219400	-2.69292600
C	5.26448600	-1.00889900	1.64762300
H	5.20888500	-0.23524100	2.42327700
H	5.64464800	-1.92281000	2.11825800
H	5.99196600	-0.68250700	0.89951400
C	-4.88807400	0.56673600	0.05097500
H	-4.51984200	1.45635000	-0.47151100
H	-5.97388500	0.66738700	0.16230200
H	-4.45175400	0.53727300	1.05090200
C	2.92478300	-1.80421000	2.05186600
H	3.30049200	-2.74557600	2.46860500
H	2.79775600	-1.09919000	2.88165500
H	1.93866100	-1.99883100	1.62870600
H	2.78119400	0.61798300	1.11079600
H	-2.86566600	-1.79470700	-1.50141500

$[Ni(CO)_3(NHC^{\beta\text{-}Me})]^{2-}$

Ni	0.03340800	1.99969900	0.01102500
C	1.58566800	2.77462700	-0.41998700
C	-1.20081700	2.53618000	-1.17142200
C	-0.32833000	2.42879200	1.70968500
O	2.52478400	3.38711200	-0.67210900
O	-1.95710400	2.90469100	-1.95089400
O	-0.52553700	2.72051500	2.80193800
N	1.01791900	-0.88065900	-0.37804300
N	-1.01474100	-0.82031600	0.28664600
C	0.02599900	-0.01414500	-0.04864700
C	-0.67808400	-2.15519700	0.17543600
H	-1.40810900	-2.91555800	0.40869000
C	0.60723100	-2.19317500	-0.24440600
H	1.29704800	-2.99561400	-0.45793600
C	2.35358300	-0.52838400	-0.84900100
H	2.31593000	0.53475600	-1.07707700
C	-2.33542500	-0.37552400	0.72392300
H	-2.25557700	0.70338000	0.84992700
C	3.47430900	-0.85284400	0.12972300
H	4.31359100	-0.17517300	-0.06249200
C	-3.46839500	-0.73705900	-0.22868700
H	-3.14580100	-0.63642700	-1.26893600
H	3.15956700	-0.65546300	1.15824600
H	-4.27960800	-0.01255700	-0.09659900
H	2.53268100	-1.08636400	-1.77104100
H	-2.53771400	-0.83805600	1.69296800
C	4.05729300	-2.29886700	0.05341900
C	-4.11111700	-2.14677800	-0.04105100
O	3.58227200	-3.05031200	-0.82915600
O	4.96102100	-2.53542900	0.87943600
O	-3.65230700	-2.85545900	0.88408800
O	-5.03827800	-2.40029600	-0.83562500

$[Ni(CO)_3(NHC^{GlyGly})]^{2-}$

Ni	0.05812600	2.23990600	0.09986300
C	-1.52568700	3.05525300	-0.09867600
C	0.72861600	2.65680700	1.71222600
C	1.08336100	2.78957400	-1.26538500

O	1.12788000	2.94548200	2.74481400
O	1.69558400	3.17711300	-2.15199700
O	-2.48023900	3.67452000	-0.23275700
N	1.01961500	-0.60811200	-0.09323200
N	-1.11541400	-0.57601500	0.01397900
C	-0.03215400	0.24401100	0.02255300
C	-0.74616800	-1.90043700	-0.12194600
H	-1.46218100	-2.70060700	-0.08713200
C	0.60052300	-1.91987400	-0.18846300
H	1.29212300	-2.74147900	-0.22613800
C	2.40090200	-0.18963500	-0.21578100
H	2.48268800	0.79283300	0.25009200
C	-2.48857200	-0.10542300	0.02969800
H	-2.77824900	0.21060700	-0.97537700
C	3.39086400	-1.13509800	0.47671800
C	-3.48495700	-1.12954400	0.58279900
H	2.65883500	-0.07634200	-1.27251700
H	-2.52510400	0.77040600	0.67761600
O	-3.19573100	-1.85532200	1.52703800
O	3.07297200	-1.78195100	1.46642300
N	-4.67157400	-1.09842000	-0.02523000
N	4.60156900	-1.12974300	-0.08403400
H	-4.88927800	-0.51953100	-0.83872600
H	4.83909400	-0.62241300	-0.93878500
C	5.76129200	-1.84810300	0.39137800
H	6.05014800	-1.51539300	1.39202800
H	5.56087400	-2.92087800	0.45256100
C	-5.83299400	-1.87452200	0.34303700
H	-5.61589300	-2.94531900	0.31123000
H	-6.15771000	-1.64052800	1.36045800
C	-6.98575900	-1.55071400	-0.65619800
C	6.94157600	-1.59828900	-0.59681100
O	-6.69671300	-0.71808100	-1.54800500
O	8.00363400	-2.16619800	-0.29935800
O	6.67148600	-0.85436700	-1.56954300
O	-8.04728500	-2.15703600	-0.44571000

Table S13. Coordinates of the optimized structures.

Precursors compounds

1f

N	0.44617500	-0.02918500	0.63505700
N	-1.23506300	-0.68701700	-0.56487100
C	-0.14841900	-1.09742800	0.08593800
C	-1.36874500	0.69020300	-0.42249500
H	-2.26592600	1.23047000	-0.76030300
C	-0.30664400	1.09569200	0.32025000
H	-0.04125100	2.07643700	0.67675200
C	1.67447000	-0.04610800	1.41555000
H	1.53852400	-0.68540600	2.29220900
C	-2.24123400	-1.57837600	-1.16777800
H	-1.82352300	-2.58310400	-1.20129600
C	2.86242800	-0.59010400	0.59859400
C	-3.47642600	-1.54536500	-0.23121300
H	0.20720900	-2.10970700	0.14474100
H	1.84696800	0.96829600	1.77477700
H	-2.44011600	-1.23190100	-2.18176100
O	-3.40635300	-2.12175900	0.84246700
O	2.69578500	-1.42329600	-0.27057100
N	-4.47356300	-0.77041000	-0.69576500
N	4.07278700	-0.12123000	0.99651800
H	-4.21669600	-0.17261000	-1.47993000
H	4.11313100	0.66997700	1.61995000
C	5.29923000	-0.51467700	0.33058300
H	6.03223200	-0.88546600	1.05249300
H	5.06438700	-1.33933100	-0.34206500
C	-5.31197600	0.04134800	0.19197900
H	-6.36809800	-0.06757200	-0.06398500
H	-5.17446900	-0.28485800	1.22171000
C	-4.87519700	1.55236600	0.02457100
C	5.97687900	0.57716700	-0.48575800
O	-4.02177500	1.75018300	-0.90351400
O	6.97271500	0.39687500	-1.12855800
O	5.34970700	1.77002500	-0.38284600
H	5.84395300	2.40567600	-0.92400900
O	-5.40193900	2.34743300	0.79462300

1g

N	-0.75379000	0.00655600	0.28463000
N	1.24975300	0.37504300	-0.48265900
C	0.08276500	0.94261800	-0.18854800
C	1.17677000	-0.97594300	-0.19059900
H	2.08199800	-1.56879300	-0.32310600
C	-0.07835100	-1.20407400	0.28662800
H	-0.53960500	-2.11085800	0.63996600
C	-2.14455700	0.22940200	0.69029800
H	-2.28492500	1.29834800	0.84520900
C	2.45104000	1.08871300	-1.00229400
H	2.09720000	2.07808900	-1.30614200
C	-3.13683600	-0.29821400	-0.34693300
H	-2.94901000	0.14673800	-1.32933100
C	3.58506400	1.13340000	0.01275200
H	3.24927600	1.53310400	0.97493400
H	-3.04265800	-1.37867000	-0.48446100
H	4.34174500	1.83068300	-0.36271100
H	-0.15479900	1.98701700	-0.30324000

H	-2.29856400	-0.24946900	1.65771800
H	2.78104800	0.53030900	-1.87696000
C	-4.55991700	0.02151700	0.05845300
C	4.28716100	-0.26732000	0.23446900
O	-4.87323700	0.69108800	1.00896200
O	-5.44446200	-0.53440100	-0.78931400
O	3.91741400	-1.17034700	-0.57309500
O	5.10066300	-0.29841400	1.15601600
H	-6.33529700	-0.29167500	-0.49158500

Carbene ligands

NHC^{GlyGly}

N	1.02061100	0.42888300	0.30441500
N	-1.02034300	0.42836600	-0.30372800
C	0.00009800	-0.43212400	0.00119500
C	-0.64664800	1.76707400	-0.19958300
H	-1.34684700	2.56605500	-0.36558500
C	0.64704600	1.76742100	0.19754200
H	1.34723200	2.56668700	0.36223400
C	2.30340900	-0.04593900	0.80208100
H	2.28649000	-1.12938600	0.69223500
C	-2.30308000	-0.04725600	-0.80071400
H	-2.28610300	-1.13054500	-0.68929000
C	3.53853100	0.58009800	0.12442700
C	-3.53825900	0.57953900	-0.12383500
H	2.38264300	0.17688000	1.87461600
H	-2.38227700	0.17399100	-1.87357100
O	-3.55476800	1.76297200	0.21490200
O	3.55543700	1.76361100	-0.21403000
N	-4.58056800	-0.25712800	-0.02198700
N	4.58026200	-0.25714000	0.02146300
H	-4.56818400	-1.21607700	-0.37009900
H	4.56777700	-1.21586500	0.37016800
C	5.93237600	0.10945200	-0.36763500
H	5.98726700	0.33637100	-1.43790800
H	6.25648100	1.01071400	0.16226300
C	-5.93266500	0.10988900	0.36669600
H	-5.98771200	0.33731000	1.43686800
H	-6.25656600	1.01099700	-0.16359400
C	-6.90883800	-1.08272800	0.03871400
C	6.90841300	-1.08316800	-0.03918200
O	-6.36059900	-2.09893900	-0.45869000
O	8.10055800	-0.86610800	-0.31759800
O	6.36011400	-2.09894300	0.45902700
O	-8.10102100	-0.86528600	0.31667500

NHC$^{\beta\text{-}Me}$

N	1.04640800	-0.23627100	0.03574500
N	-1.04493200	0.21625900	0.07539300
C	0.00136000	0.06764700	-0.79853300
C	-0.66319100	0.01315200	1.39349400
H	-1.35470000	0.08420600	2.21915100
C	0.66276400	-0.27398700	1.36832000
H	1.35315700	-0.49414300	2.16822700
C	2.42199200	-0.50416700	-0.41606500
H	2.36124300	-0.59784800	-1.50075500
C	-2.41932700	0.56412900	-0.32281300
H	-2.35907400	0.83437700	-1.37745200
C	3.45463500	0.54785800	-0.01488300
H	3.30344500	1.48084100	-0.56618000

C	-3.45823700	-0.53322900	-0.09824100
H	-3.33721200	-0.95059100	0.91183900
H	3.32772800	0.79726900	1.04854200
H	-3.30859000	-1.36679200	-0.79115300
H	2.75562400	-1.46700600	-0.02308200
H	-2.74560400	1.45173900	0.22348200
C	4.95071600	0.07229200	-0.19452600
C	-4.95165900	-0.02833700	-0.20420800
O	5.78337200	0.99493100	-0.33025700
O	5.13895200	-1.16789100	-0.14480400
O	-5.13434200	1.19015200	0.03596700
O	-5.78806600	-0.91520700	-0.48163800

NHC^{Gly}

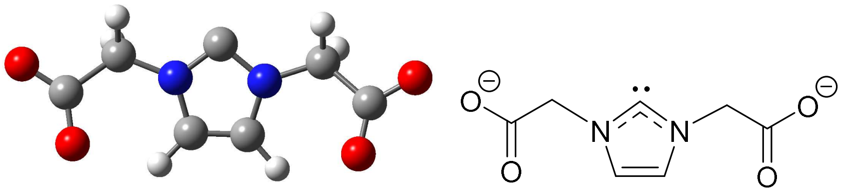

O	-3.43047500	1.32293100	-0.35157000
O	3.43048700	1.32293400	-0.35155200
O	4.72066500	-0.53555800	-0.34024100
N	-1.07063000	-0.35794200	0.17966300
N	1.07062900	-0.35793700	0.17967700
C	0.00000200	-1.18559700	-0.04666300
C	0.67902100	0.92311300	0.55386200
H	1.41196500	1.70081100	0.68197100
C	-0.67903000	0.92312200	0.55381000
H	-1.41197400	1.70082800	0.68187400
C	-2.45349200	-0.85256400	0.17895800
H	-2.46685000	-1.72541900	-0.47309600
C	-3.64473200	0.09744800	-0.22916500
C	2.45349200	-0.85255600	0.17897700
H	2.69679000	-1.22009000	1.18587400
C	3.64473500	0.09744900	-0.22915700
O	-4.72067200	-0.53554900	-0.34020200
H	2.46685200	-1.72542100	-0.47306400
H	-2.69679300	-1.22011400	1.18584800

(*S*,*S*)-NHC^{Ala}

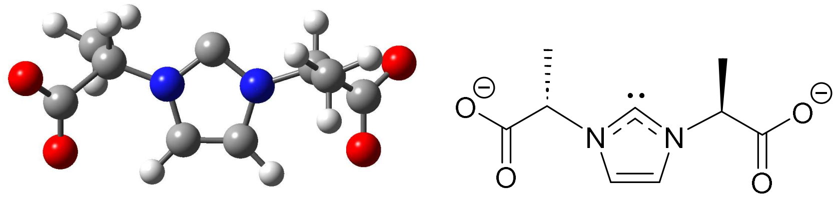

O	-3.59414600	1.40635000	-0.20795600
O	3.59419200	1.40664700	0.20558500
O	4.42681800	-0.52252700	1.04354500
N	-1.06760300	-0.17920800	0.07740300
N	1.06760600	-0.17935800	-0.07699300
C	-0.00001800	-1.03681600	0.00076100
C	0.67637300	1.15682900	-0.05614200
H	1.41556700	1.93895200	-0.09139400
C	-0.67630400	1.15693700	0.05480800

H	-1.41544300	1.93916000	0.08901800
C	-2.44565700	-0.66735000	0.27865300
H	-2.45666400	-1.66714700	-0.15232900
C	-3.59223700	0.16818100	-0.41912100
C	2.44564600	-0.66782200	-0.27762200
H	2.45671900	-1.66694800	0.15492000
C	3.59228800	0.16881500	0.41872800
O	-4.42672400	-0.52414500	-1.04290400
C	2.74968600	-0.76374900	-1.77928000
H	1.99074500	-1.36455600	-2.29180600
H	2.77007500	0.23749400	-2.21865400
H	3.73380500	-1.22241300	-1.93014700
C	-2.74987600	-0.76097700	1.78042900
H	-3.73404000	-1.21935200	1.93186500
H	-1.99104300	-1.36105600	2.29397300
H	-2.77027300	0.24093700	2.21825200

(*S*,*S*)-NHC^{Val}

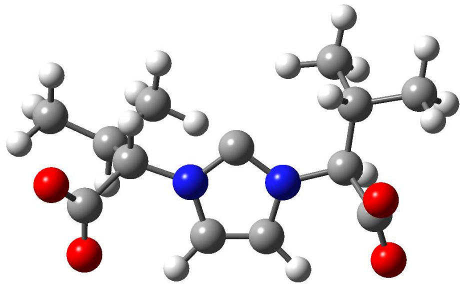

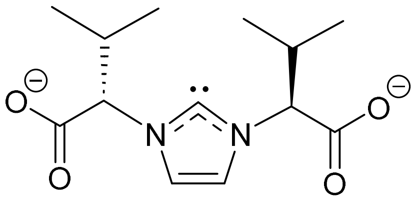

O	-3.47374300	-1.99112100	-0.19682900
O	3.74828500	-1.45879900	1.16223700
O	3.54492300	-2.00287800	-1.02447700
N	-1.02077400	-0.31480600	-0.02032600
N	1.09790300	-0.25490200	-0.33906400
C	0.11335500	0.32517600	0.41680100
C	0.59054600	-1.21994500	-1.20885300
H	1.25490700	-1.81611600	-1.81251200
C	-0.74952000	-1.25490200	-1.01007600
H	-1.53717700	-1.87833700	-1.39740000
C	-2.37117800	0.06054600	0.43305300
H	-2.21435800	0.61002800	1.36142600
C	-3.33127900	-1.16098200	0.73454900
C	-3.07896200	0.98364300	-0.59917400
H	-3.29155800	0.35148300	-1.47044400
C	-2.21848800	2.16896100	-1.05159300
H	-2.75644600	2.77802600	-1.79185300
H	-1.27909400	1.84040800	-1.49805600
H	-1.96150800	2.81528900	-0.20521900
C	-4.41984100	1.48545900	-0.04192700
H	-4.25717800	2.13015200	0.83071000
H	-5.05398300	0.66042000	0.28410800
H	-4.96157800	2.07327300	-0.79542200
C	2.55105500	0.05806700	-0.31730500
H	2.81507300	0.33053700	-1.34865900
C	3.37196300	-1.27905300	-0.01552000
C	2.89686700	1.25128900	0.59219500
H	2.51351500	1.01595500	1.58895400
C	2.25979700	2.56596900	0.11494100
H	2.56146600	3.40162200	0.76229300
H	1.17329700	2.49004600	0.13292400
H	2.58457900	2.80980200	-0.90642100
C	4.42173200	1.42504600	0.68788700
H	4.87361100	0.53616100	1.12481400
H	4.67048600	2.29894500	1.30510900

H	4.86398600	1.59080400	-0.30465800
O	-3.91374500	-1.11047600	1.83984000

(*S*,*S*)-NHC^{Leu}

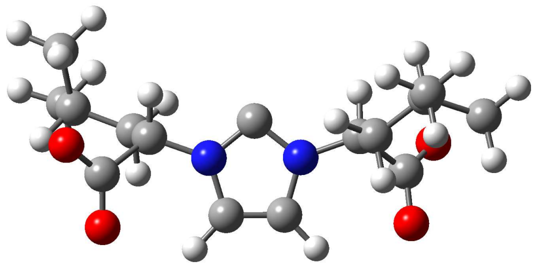

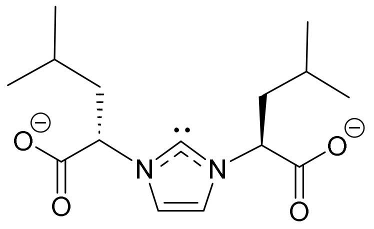

O	-3.26383100	1.02174800	2.19012900
O	3.44770300	-1.57726600	1.57557400
O	3.57875900	-2.55825600	-0.45792200
N	-1.03909600	0.18184800	0.39351500
N	1.03381800	-0.33079300	0.25660700
C	-0.01620200	0.09360300	-0.51545700
C	0.67765300	-0.49206200	1.59325000
H	1.40204500	-0.82543200	2.31692500
C	-0.64008400	-0.17980800	1.67766200
H	-1.33426300	-0.12805800	2.49865600
C	-2.41446600	0.51940000	-0.01853300
H	-2.28864000	1.09140200	-0.93583200
C	-3.24834400	1.39536600	0.99380100
C	2.39839300	-0.47505800	-0.29028500
H	2.25389500	-0.68810400	-1.34955500
C	3.20837200	-1.67325800	0.34715600
O	-3.88652000	2.33316900	0.45748900
C	3.14564400	0.86584500	-0.13096600
H	2.42466200	1.66458000	-0.34303700
H	3.44195300	0.96258800	0.92074900
C	-3.18069500	-0.78492900	-0.32504800
H	-2.60540600	-1.34509000	-1.07564100
H	-3.18547300	-1.38872000	0.58996700
C	4.37283400	1.07861300	-1.04025200
H	4.04503900	0.90508500	-2.07628100
C	4.84554500	2.53788000	-0.94564000
H	5.16995900	2.77424800	0.07513500
H	5.69383100	2.73225800	-1.61469800
H	4.04440500	3.23801600	-1.20496900
C	5.53695200	0.11934800	-0.75411700
H	6.39203600	0.34022700	-1.40874800
H	5.87233200	0.21315400	0.28477700
H	5.24069400	-0.91970800	-0.90464500
C	-4.63362900	-0.61567200	-0.80646700
H	-5.19701200	-0.14423800	0.00711800
C	-4.74836200	0.30551600	-2.02802400
H	-5.77886100	0.33087500	-2.40787400
H	-4.46428400	1.32020000	-1.74540100
H	-4.09904700	-0.03382400	-2.84517200
C	-5.26011200	-1.98958400	-1.08588200
H	-5.20000700	-2.64235200	-0.20804600
H	-6.31798400	-1.90128400	-1.36537300
H	-4.74281600	-2.49956600	-1.90826100

(*S*,*S*)-NHC^{iLeu}

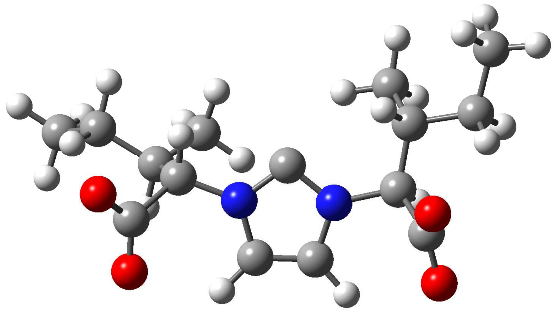

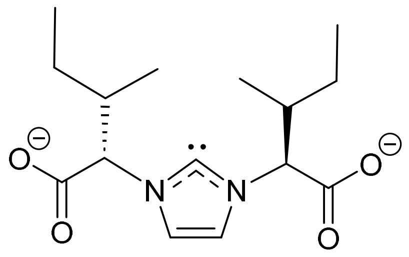

O	-3.44662200	-2.20792400	0.15727800
O	3.78916500	-1.67824100	0.99102000
O	3.45677100	-2.37426000	-1.13594500
N	-0.99469000	-0.53906100	0.03346000
N	1.09338800	-0.52285100	-0.44798000
C	0.17623100	0.10440600	0.35324000
C	0.51115500	-1.51861500	-1.23126500
H	1.12081200	-2.15511300	-1.85116800
C	-0.81005300	-1.52654600	-0.92948100
H	-1.62936700	-2.15863800	-1.22603100
C	-2.29912600	-0.12626200	0.57633300
H	-2.05824800	0.46881400	1.45737800
C	-3.23582000	-1.32332400	1.02167700
C	-3.09755800	0.74920000	-0.43457000
H	-3.48006800	0.05286100	-1.19241300
C	-2.23774300	1.81077800	-1.13282600
H	-2.84073500	2.43348100	-1.80673200
H	-1.44211000	1.35562200	-1.72487800
H	-1.75561200	2.46677600	-0.40106300
C	-4.30824100	1.39205900	0.27670700
H	-3.95671800	2.26375100	0.84776000
H	-4.69847300	0.68827200	1.01583200
C	2.55074400	-0.24214500	-0.53725800
H	2.75569300	-0.06360600	-1.60160100
C	3.35579300	-1.57437300	-0.17673100
C	2.95937700	1.02123600	0.24758300
H	2.60217200	0.88685000	1.27333700
C	2.31209900	2.28659500	-0.33919400
H	2.56828600	3.18162200	0.24038300
H	1.22757400	2.18901800	-0.33214100
H	2.65021100	2.45336000	-1.37174100
C	4.49668300	1.15349300	0.28583200
H	4.89211100	0.21383800	0.67105700
H	4.87932700	1.27195600	-0.73995500
O	-3.73653500	-1.20456300	2.16299700
C	-5.43853200	1.82210400	-0.66515500
H	-6.27089600	2.27410200	-0.11109400
H	-5.10780100	2.55532500	-1.40989500
H	-5.83766100	0.95911900	-1.20884600
C	5.01714800	2.30633300	1.15301600
H	6.11236100	2.28419900	1.21853000
H	4.73504000	3.29308000	0.76998600
H	4.62803000	2.22896900	2.17470400

Silver complexes

$[Ag(NHC^{GlyGly})_2]^{3-}$

Ag	0.00000000	0.00000000	0.00022100
N	-0.96920700	2.97112200	0.46942700
N	0.96929300	2.97090500	-0.46928600
C	0.00000000	2.14120300	0.00016900
C	0.61321200	4.29982000	-0.28649800
H	1.25674000	5.09034900	-0.62502900
C	-0.61282100	4.29995900	0.28663800
H	-1.25619300	5.09063800	0.62510500
C	-2.26617900	2.51376100	0.98401300
H	-2.15051000	1.46723400	1.26302100
C	2.26610600	2.51328800	-0.98402100
H	2.15022900	1.46678100	-1.26302400
C	-2.81981800	3.35499300	2.15694900
C	2.81972000	3.35448200	-2.15698600
H	-2.99967300	2.56332300	0.17211600
H	2.99969400	2.56273600	-0.17220400
O	2.71216300	4.58214200	-2.16294500
O	-2.71230000	4.58265600	2.16289200
N	3.46852400	2.64255900	-3.07930200
N	-3.46871700	2.64309400	3.07921400
H	3.61787200	1.63081500	-3.03742500
H	-3.61806900	1.63135000	3.03734000
C	-4.25586200	3.20004200	4.17016000
H	-3.60830700	3.65733800	4.92505300
H	-4.92290800	3.98497400	3.80194000
C	4.25557700	3.19948000	-4.17032700
H	3.60795800	3.65677200	-4.92516800
H	4.92266400	3.98441000	-3.80217900
C	5.09686600	2.04101900	-4.82948300
C	-5.09722600	2.04160100	4.82925400
O	4.91178500	0.90492800	-4.32516300
O	-5.83200900	2.40564500	5.76161700
O	-4.91209800	0.90549800	4.32497700
O	5.83156700	2.40504100	-5.76191900
N	0.96920700	-2.97112200	0.46942700
N	-0.96929300	-2.97090500	-0.46928600
C	0.00000000	-2.14120300	0.00016900
C	-0.61321200	-4.29982000	-0.28649800
H	-1.25674000	-5.09034900	-0.62502900
C	0.61282100	-4.29995900	0.28663800
H	1.25619300	-5.09063800	0.62510500
C	2.26617900	-2.51376100	0.98401300
H	2.15051000	-1.46723400	1.26302100
C	-2.26610600	-2.51328800	-0.98402100
H	-2.15022900	-1.46678100	-1.26302400
C	2.81981800	-3.35499300	2.15694900
C	-2.81972000	-3.35448200	-2.15698600
H	2.99967300	-2.56332300	0.17211600
H	-2.99969400	-2.56273600	-0.17220400
O	-2.71216300	-4.58214200	-2.16294500
O	2.71230000	-4.58265600	2.16289200
N	-3.46852400	-2.64255900	-3.07930200
N	3.46871700	-2.64309400	3.07921400
H	-3.61787200	-1.63081500	-3.03742500
H	3.61806900	-1.63135000	3.03734000
C	4.25586200	-3.20004200	4.17016000
H	3.60830700	-3.65733800	4.92505300
H	4.92290800	-3.98497400	3.80194000
C	-4.25557700	-3.19948000	-4.17032700
H	-3.60795800	-3.65677200	-4.92516800
H	-4.92266400	-3.98441000	-3.80217900
C	-5.09686600	-2.04101900	-4.82948300
C	5.09722600	-2.04160100	4.82925400

O	-4.91178500	-0.90492800	-4.32516300
O	5.83200900	-2.40564500	5.76161700
O	4.91209800	-0.90549800	4.32497700
O	-5.83156700	-2.40504100	-5.76191900

$[Ag(NHC^{\beta\text{-}Me})_2]^{3-}$

Ag	0.00001400	-0.00007800	0.00001900
N	2.98257200	-0.98176100	-0.43996400
N	2.98246700	0.98179900	0.44003000
C	2.15158000	-0.00005800	0.00009800
C	4.31114700	0.62048800	0.27615000
H	5.08754100	1.30803100	0.57851600
C	4.31121400	-0.62020200	-0.27631900
H	5.08767700	-1.30761900	-0.57879900
C	2.55662100	-2.27485600	-1.00018600
H	1.46713300	-2.28117600	-0.92408800
C	2.55637700	2.27477200	1.00042200
H	1.46687600	2.28090600	0.92450700
C	3.02408400	-2.53073900	-2.43610100
H	2.32506600	-3.22954900	-2.91118300
C	3.02402500	2.53063000	2.43628200
H	2.96464500	1.60962200	3.02680200
H	2.96450600	-1.60977100	-3.02666200
H	2.32498400	3.22932600	2.91150200
H	2.96150400	-3.06037500	-0.35834400
H	2.96100600	3.06041800	0.35857400
C	4.46663000	-3.14628000	-2.60357500
C	4.46651100	3.14635400	2.60358300
O	5.14003400	-3.28389300	-1.54881300
O	4.76848100	-3.43738800	-3.77706200
O	5.13970300	3.28422300	1.54872200
O	4.76854800	3.43729100	3.77706800
N	-2.98245600	0.98173800	-0.44012500
N	-2.98253000	-0.98177900	0.43997000
C	-2.15155900	-0.00006000	-0.00009500
C	-4.31117900	-0.62028700	0.27622200
H	-5.08763800	-1.30770900	0.57869700
C	-4.31113300	0.62036000	-0.27634600
H	-5.08753200	1.30784800	-0.57882800
C	-2.55639600	2.27471800	-1.00052500
H	-1.46689500	2.28089000	-0.92460300
C	-2.55655800	-2.27480500	1.00032900
H	-1.46706500	-2.28108600	0.92429600
C	-3.02406500	2.53055000	-2.43638100
H	-2.32505400	3.22926000	-2.91162100
C	-3.02409300	-2.53058300	2.43623900
H	-2.96456700	-1.60956800	3.02673300
H	-2.96467500	1.60953600	-3.02689000
H	-2.32508100	-3.22933800	2.91141300
H	-2.96106200	3.06034900	-0.35868400
H	-2.96136700	-3.06040100	0.35853300
C	-4.46657700	3.14622300	-2.60365000
C	-4.46663100	-3.14615000	2.60368400
O	-5.13975900	3.28404300	-1.54877300
O	-4.76862700	3.43721500	-3.77711600
O	-5.13994300	-3.28393600	1.54889000
O	-4.76856500	-3.43709900	3.77719200

$Na_3[Ag(NHC^{\beta\text{-}Me})_2]$, **2g**

Ag	-0.26441100	0.06107000	-0.39706300
N	0.60971900	3.10257800	-0.79568700
N	-1.41552600	2.95369200	-0.08765300
C	-0.35441000	2.20330900	-0.46339500

C	-1.12131300	4.30075400	-0.16967000
H	-1.84406100	5.06080600	0.07653200
C	0.15619400	4.39889200	-0.61591800
H	0.76359900	5.26070800	-0.83784300
C	1.93290700	2.73776700	-1.30501300
H	2.21968200	3.46655500	-2.06481900
C	-2.72329900	2.40065100	0.31705100
H	-2.94918800	1.56736300	-0.35096300
C	2.99928700	2.62875900	-0.21182400
H	3.35692600	3.61040400	0.11378200
C	-2.78218100	1.95320900	1.78177200
H	-2.80674700	2.82247300	2.44576400
H	2.56576600	2.15797900	0.67659800
H	-1.91483200	1.33758700	2.02921800
H	1.83181300	1.78210000	-1.81265900
H	-3.47760800	3.16373100	0.12961500
C	4.19843300	1.77895400	-0.64498500
C	-4.07318500	1.11837500	1.91971900
O	4.20628900	1.29876200	-1.80159700
O	5.10415100	1.55559900	0.22331500
O	-5.15585300	1.74918300	1.79505200
O	-3.94559900	-0.12926500	2.00893500
N	-1.28516000	-2.89111200	-0.31692400
N	0.86317600	-2.92885600	-0.41051600
C	-0.20037900	-2.08323400	-0.33865000
C	0.44151900	-4.24864600	-0.43620200
H	1.12899200	-5.07681900	-0.48379900
C	-0.91350400	-4.21956300	-0.38343700
H	-1.63759500	-5.01736000	-0.38453500
C	-2.67812800	-2.41127300	-0.22060800
H	-2.69107900	-1.57847000	0.48505700
C	2.26020700	-2.50507700	-0.31391300
H	2.27336200	-1.44274300	-0.55597300
C	-3.28762300	-1.99497000	-1.56362200
H	-2.60679900	-1.33561900	-2.10587200
C	2.84248700	-2.75049800	1.08783100
H	3.12310700	-3.80279200	1.20742300
H	-3.49807000	-2.87645700	-2.17660700
H	2.08864500	-2.53759500	1.84661400
H	-3.27173400	-3.20748900	0.22684000
H	2.83939300	-3.02150900	-1.08179800
C	-4.58913300	-1.23881500	-1.22126200
C	4.06719600	-1.88449000	1.39031700
O	-4.57702400	0.01252600	-1.34118800
O	-5.51422400	-1.93017100	-0.71904100
O	4.87211300	-1.62347300	0.43568300
O	4.19421100	-1.44507000	2.55414700
Na	5.52029600	-0.39525500	-1.21349200
Na	5.37448000	0.33210000	1.99708400
Na	-5.89429300	-0.10653800	0.64034200

$[Ag(NHC^{Gly})_2]^{3-}$

Ag	0.00001400	-0.00007800	0.00001900
N	2.98257200	-0.98176100	-0.43996400
N	2.98246700	0.98179900	0.44003000
C	2.15158000	-0.00005800	0.00009800
C	4.31114700	0.62048800	0.27615000
H	5.08754100	1.30803100	0.57851600
C	4.31121400	-0.62020200	-0.27631900
H	5.08767700	-1.30761900	-0.57879900
C	2.55662100	-2.27485600	-1.00018600
H	1.46713300	-2.28117600	-0.92408800
C	2.55637700	2.27477200	1.00042200
H	1.46687600	2.28090600	0.92450700
C	3.02408400	-2.53073900	-2.43610100
H	2.32506600	-3.22954900	-2.91118300
C	3.02402500	2.53063000	2.43628200

H	2.96464500	1.60962200	3.02680200
H	2.96450600	-1.60977100	-3.02666200
H	2.32498400	3.22932600	2.91150200
H	2.96150400	-3.06037500	-0.35834400
H	2.96100600	3.06041800	0.35857400
C	4.46663000	-3.14628000	-2.60357500
C	4.46651100	3.14635400	2.60358300
O	5.14003400	-3.28389300	-1.54881300
O	4.76848100	-3.43738800	-3.77706200
O	5.13970300	3.28422300	1.54872200
O	4.76854800	3.43729100	3.77706800
N	-2.98245600	0.98173800	-0.44012500
N	-2.98253000	-0.98177900	0.43997000
C	-2.15155900	-0.00006000	-0.00009500
C	-4.31117900	-0.62028700	0.27622200
H	-5.08763800	-1.30770900	0.57869700
C	-4.31113300	0.62036000	-0.27634600
H	-5.08753200	1.30784800	-0.57882800
C	-2.55639600	2.27471800	-1.00052500
H	-1.46689500	2.28089000	-0.92460300
C	-2.55655800	-2.27480500	1.00032900
H	-1.46706500	-2.28108600	0.92429600
C	-3.02406500	2.53055000	-2.43638100
H	-2.32505400	3.22926000	-2.91162100
C	-3.02409300	-2.53058300	2.43623900
H	-2.96456700	-1.60956800	3.02673300
H	-2.96467500	1.60953600	-3.02689000
H	-2.32508100	-3.22933800	2.91141300
H	-2.96106200	3.06034900	-0.35868400
H	-2.96136700	-3.06040100	0.35853300
C	-4.46657700	3.14622300	-2.60365000
C	-4.46663100	-3.14615000	2.60368400
O	-5.13975900	3.28404300	-1.54877300
O	-4.76862700	3.43721500	-3.77711600
O	-5.13994300	-3.28393600	1.54889000
O	-4.76856500	-3.43709900	3.77719200

$[Ag(NHC^{Ala})_2]^{3-}$

Ag	0.00001400	-0.00007800	0.00001900
N	2.98257200	-0.98176100	-0.43996400
N	2.98246700	0.98179900	0.44003000
C	2.15158000	-0.00005800	0.00009800
C	4.31114700	0.62048800	0.27615000
H	5.08754100	1.30803100	0.57851600
C	4.31121400	-0.62020200	-0.27631900
H	5.08767700	-1.30761900	-0.57879900
C	2.55662100	-2.27485600	-1.00018600
H	1.46713300	-2.28117600	-0.92408800
C	2.55637700	2.27477200	1.00042200
H	1.46687600	2.28090600	0.92450700
C	3.02408400	-2.53073900	-2.43610100
H	2.32506600	-3.22954900	-2.91118300
C	3.02402500	2.53063000	2.43628200
H	2.96464500	1.60962200	3.02680200
H	2.96450600	-1.60977100	-3.02666200
H	2.32498400	3.22932600	2.91150200
H	2.96150400	-3.06037500	-0.35834400
H	2.96100600	3.06041800	0.35857400
C	4.46663000	-3.14628000	-2.60357500
C	4.46651100	3.14635400	2.60358300
O	5.14003400	-3.28389300	-1.54881300
O	4.76848100	-3.43738800	-3.77706200
O	5.13970300	3.28422300	1.54872200
O	4.76854800	3.43729100	3.77706800
N	-2.98245600	0.98173800	-0.44012500
N	-2.98253000	-0.98177900	0.43997000
C	-2.15155900	-0.00006000	-0.00009500

C	-4.31117900	-0.62028700	0.27622200
H	-5.08763800	-1.30770900	0.57869700
C	-4.31113300	0.62036000	-0.27634600
H	-5.08753200	1.30784800	-0.57882800
C	-2.55639600	2.27471800	-1.00052500
H	-1.46689500	2.28089000	-0.92460300
C	-2.55655800	-2.27480500	1.00032900
H	-1.46706500	-2.28108600	0.92429600
C	-3.02406500	2.53055000	-2.43638100
H	-2.32505400	3.22926000	-2.91162100
C	-3.02409300	-2.53058300	2.43623900
H	-2.96456700	-1.60956800	3.02673300
H	-2.96467500	1.60953600	-3.02689000
H	-2.32508100	-3.22933800	2.91141300
H	-2.96106200	3.06034900	-0.35868400
H	-2.96136700	-3.06040100	0.35853300
C	-4.46657700	3.14622300	-2.60365000
C	-4.46663100	-3.14615000	2.60368400
O	-5.13975900	3.28404300	-1.54877300
O	-4.76862700	3.43721500	-3.77711600
O	-5.13994300	-3.28393600	1.54889000
O	-4.76856500	-3.43709900	3.77719200

$[Ag(NHC^{Val})_2]^{3-}$

Ag	0.00000000	0.00000000	0.01860000
O	2.20820000	-3.28380000	3.25440000
O	5.08150000	2.34400000	-1.81400000
O	3.32010000	2.75470000	-3.16890000
N	2.84230000	-1.39980000	0.43420000
N	3.10870000	0.55320000	-0.45220000
C	2.14930000	-0.30680000	0.00240000
C	4.37290000	0.00230000	-0.30430000
H	5.23880000	0.55500000	-0.62530000
C	4.20480000	-1.22420000	0.24750000
H	4.89540000	-1.97910000	0.58090000
C	2.25790000	-2.66350000	0.93460000
H	1.21450000	-2.44220000	1.16190000
C	2.96130000	-3.15750000	2.26850000
C	2.84100000	1.91230000	-0.98520000
H	1.86330000	1.84740000	-1.46160000
C	3.85910000	2.36510000	-2.11240000
O	4.18720000	-3.42130000	2.16590000
O	-5.08140000	-2.34410000	-1.81400000
O	-2.20820000	3.28380000	3.25430000
O	-4.18720000	3.42140000	2.16590000
N	-3.10860000	-0.55320000	-0.45230000
N	-2.84240000	1.39980000	0.43410000
C	-2.14930000	0.30680000	0.00240000
C	-4.20480000	1.22410000	0.24750000
H	-4.89540000	1.97900000	0.58080000
C	-4.37290000	-0.00230000	-0.30430000
H	-5.23870000	-0.55500000	-0.62530000
C	-2.84100000	-1.91230000	-0.98520000
H	-1.86330000	-1.84740000	-1.46150000
C	-3.85900000	-2.36520000	-2.11240000
C	-2.25800000	2.66350000	0.93450000
H	-1.21450000	2.44230000	1.16180000
C	-2.96130000	3.15760000	2.26840000
O	-3.31990000	-2.75480000	-3.16880000
C	-2.74010000	-2.95050000	0.16850000
H	-1.89430000	-2.62690000	0.78920000
C	-2.34830000	3.77220000	-0.14550000
H	-3.41770000	3.96650000	-0.28790000
C	-1.73900000	3.35980000	-1.49180000
H	-2.23010000	2.48000000	-1.91190000
H	-1.83470000	4.17550000	-2.21810000
H	-0.67360000	3.12890000	-1.39690000

C	-1.70350000	5.06870000	0.36810000
H	-2.17680000	5.40920000	1.29120000
H	-0.63780000	4.92330000	0.57100000
H	-1.79490000	5.86120000	-0.38410000
C	-3.97880000	-3.01640000	1.07340000
H	-4.86260000	-3.29590000	0.49500000
H	-3.81640000	-3.75530000	1.86720000
H	-4.17760000	-2.05480000	1.55230000
C	-2.40770000	-4.34040000	-0.39440000
H	-1.53670000	-4.31050000	-1.05330000
H	-2.19520000	-5.03850000	0.42260000
H	-3.24500000	-4.73270000	-0.97890000
C	2.74020000	2.95050000	0.16850000
H	1.89440000	2.62690000	0.78910000
C	3.97890000	3.01640000	1.07330000
H	4.86270000	3.29590000	0.49490000
H	3.81660000	3.75530000	1.86710000
H	4.17770000	2.05480000	1.55230000
C	2.40780000	4.34040000	-0.39450000
H	1.53680000	4.31040000	-1.05340000
H	2.19520000	5.03850000	0.42250000
H	3.24510000	4.73270000	-0.97900000
C	2.34810000	-3.77220000	-0.14530000
H	3.41760000	-3.96650000	-0.28780000
C	1.73880000	-3.35980000	-1.49160000
H	2.23000000	-2.48000000	-1.91180000
H	1.83450000	-4.17550000	-2.21800000
H	0.67340000	-3.12890000	-1.39680000
C	1.70340000	-5.06870000	0.36830000
H	2.17670000	-5.40920000	1.29130000
H	0.63760000	-4.92320000	0.57120000
H	1.79470000	-5.86120000	-0.38390000

$[Ag(NHC^{iLeu})_2]^{3-}$

Ag	0.00530000	0.03910000	0.09040000
O	-3.18560000	-3.04690000	2.36820000
O	4.16080000	-4.04880000	-0.69610000
O	4.40750000	-2.21330000	-1.99030000
N	-0.55830000	-3.08640000	0.19040000
N	1.56250000	-2.74280000	-0.04540000
C	0.36830000	-2.09300000	0.07310000
C	1.38010000	-4.11690000	0.00200000
H	2.22780000	-4.77390000	-0.08610000
C	0.04900000	-4.33180000	0.14580000
H	-0.53680000	-5.22450000	0.27840000
C	-2.02410000	-2.90560000	0.27040000
H	-2.18260000	-1.84510000	0.46770000
C	-2.63500000	-3.72920000	1.47990000
C	2.87840000	-2.06160000	-0.16270000
H	2.67190000	-1.13510000	-0.69780000
C	3.90950000	-2.86790000	-1.04900000
O	-2.53240000	-4.97870000	1.38650000
O	-4.24980000	3.97640000	-0.82830000
O	2.96040000	3.36980000	2.55050000
O	2.35550000	5.22030000	1.39690000
N	-1.60110000	2.76800000	-0.11780000
N	0.49240000	3.19170000	0.21570000
C	-0.39160000	2.16370000	0.06700000
C	-0.15650000	4.41340000	0.11990000
H	0.39210000	5.32810000	0.26320000
C	-1.47040000	4.14780000	-0.08400000
H	-2.33760000	4.77160000	-0.21640000
C	-2.88660000	2.04270000	-0.28880000
H	-2.63150000	1.12950000	-0.82550000
C	-3.91710000	2.82280000	-1.20600000
C	1.95730000	3.06910000	0.38560000

H	2.14200000	2.02770000	0.65090000
C	2.46850000	3.98080000	1.58150000
O	-4.32250000	2.17800000	-2.19560000
C	-3.48370000	1.62830000	1.08680000
H	-2.75190000	0.94160000	1.53430000
C	2.70280000	3.43020000	-0.92690000
H	2.45310000	4.47800000	-1.13550000
C	2.25730000	2.56870000	-2.12390000
H	1.16590000	2.51920000	-2.14730000
H	2.60750000	1.53930000	-1.98200000
C	4.22160000	3.34550000	-0.70370000
H	4.53170000	3.99400000	0.11740000
H	4.52390000	2.32190000	-0.45960000
H	4.76880000	3.64990000	-1.60170000
C	-3.67580000	2.80350000	2.05720000
H	-4.35400000	3.54470000	1.62730000
H	-4.08310000	2.44810000	3.00920000
H	-2.72620000	3.29920000	2.27390000
C	-4.79270000	0.83930000	0.87460000
H	-4.67310000	0.18320000	0.00660000
H	-5.58530000	1.54570000	0.60120000
C	3.41110000	-1.67400000	1.24710000
H	2.61280000	-1.05720000	1.68010000
C	3.61950000	-2.86430000	2.19550000
H	4.32200000	-3.58800000	1.77850000
H	3.99740000	-2.50720000	3.16130000
H	2.67700000	-3.38500000	2.38130000
C	4.65140000	-0.74880000	1.17630000
H	4.47900000	0.00360000	0.40010000
H	4.69550000	-0.19400000	2.12110000
C	-2.70610000	-3.30970000	-1.06420000
H	-2.47620000	-4.37250000	-1.21080000
C	-2.16480000	-2.51710000	-2.26950000
H	-1.07250000	-2.50930000	-2.23540000
H	-2.48220000	-1.47070000	-2.18760000
C	-4.23200000	-3.17430000	-0.93810000
H	-4.60770000	-3.75710000	-0.09540000
H	-4.52100000	-2.12970000	-0.78480000
C	6.01490000	-1.41020000	0.92730000
H	6.04950000	-1.91970000	-0.03650000
H	6.80050000	-0.64490000	0.93430000
H	6.25810000	-2.13840000	1.70820000
C	2.74370000	3.08300000	-3.48560000
H	2.35040000	2.46190000	-4.29700000
H	2.40490000	4.11140000	-3.65830000
H	3.83490000	3.07170000	-3.56620000
C	-5.22100000	-0.00870000	2.07950000
H	-5.36330000	0.60260000	2.97790000
H	-6.17390000	-0.51100000	1.87500000
H	-4.48730000	-0.78870000	2.30570000
C	-2.59920000	-3.06930000	-3.63380000
H	-2.30300000	-4.11950000	-3.74340000
H	-3.68220000	-3.00950000	-3.77800000
H	-2.13060000	-2.50470000	-4.44650000
H	-4.73250000	-3.52830000	-1.84500000

$[Ag(NHC^{Leu})_2]^{3-}$

Ag	-0.03520000	0.03180000	0.10020000
O	0.49900000	-3.89370000	-3.34620000
O	-4.34800000	-2.35730000	2.89790000
O	-4.50380000	-0.12640000	3.22070000
N	-0.93700000	-2.98940000	-0.34710000
N	-2.45440000	-1.92270000	0.76460000
C	-1.24420000	-1.76100000	0.15390000
C	-2.88750000	-3.23430000	0.64630000
H	-3.79350000	-3.55250000	1.12750000
C	-1.93330000	-3.90540000	-0.04530000

H	-1.84870000	-4.92330000	-0.38400000
C	0.33840000	-3.34550000	-1.01750000
H	0.73600000	-2.40850000	-1.40800000
C	0.10780000	-4.32300000	-2.24020000
C	-3.23930000	-0.81100000	1.35430000
H	-2.49540000	-0.09300000	1.70250000
C	-4.11150000	-1.15640000	2.63130000
O	-0.39280000	-5.44060000	-1.95820000
O	4.35210000	2.16030000	2.92170000
O	-0.46150000	4.15360000	-3.28280000
O	0.59630000	5.56650000	-1.86810000
N	2.43220000	1.88030000	0.78640000
N	0.97570000	3.04780000	-0.30560000
C	1.21760000	1.79410000	0.16870000
C	2.01660000	3.90460000	0.02120000
H	1.98710000	4.93180000	-0.29860000
C	2.93210000	3.17020000	0.69990000
H	3.85270000	3.42980000	1.18860000
C	3.16140000	0.71400000	1.34230000
H	2.38330000	0.02900000	1.68250000
C	4.06150000	0.98000000	2.61870000
C	-0.26360000	3.48480000	-0.99330000
H	-0.69450000	2.58160000	-1.42190000
C	0.00640000	4.50070000	-2.17820000
O	4.41300000	-0.08470000	3.17080000
C	3.94030000	-0.02270000	0.22660000
H	3.20590000	-0.35400000	-0.51650000
C	-1.22010000	4.10760000	0.04420000
H	-0.66220000	4.88310000	0.57940000
C	-2.49860000	4.74420000	-0.53160000
H	-2.18350000	5.53230000	-1.22480000
C	5.05180000	0.74870000	-0.51710000
H	4.65580000	1.73280000	-0.79500000
C	-4.06940000	-0.09220000	0.26450000
H	-3.36240000	0.28880000	-0.48120000
C	-5.15410000	-0.90170000	-0.47870000
H	-4.71900000	-1.86470000	-0.77130000
C	1.30330000	-3.93120000	0.03400000
H	0.92240000	-4.91310000	0.33910000
C	2.78160000	-4.05810000	-0.39140000
H	3.07070000	-3.11610000	-0.87510000
H	1.25840000	-3.27920000	0.91410000
H	4.36390000	-0.92060000	0.68680000
H	-1.49150000	3.33790000	0.77890000
H	-4.52860000	0.77520000	0.74940000
C	3.66520000	-4.22830000	0.85540000
H	3.39310000	-5.14210000	1.39930000
H	4.72300000	-4.30910000	0.57990000
H	3.56420000	-3.38550000	1.54450000
C	3.04530000	-5.19740000	-1.38830000
H	2.74720000	-6.16110000	-0.95890000
H	2.49310000	-5.06180000	-2.31870000
H	4.11530000	-5.24940000	-1.62740000
C	-5.55180000	-0.16920000	-1.77090000
H	-6.31020000	-0.73390000	-2.32640000
H	-5.97020000	0.81930000	-1.54540000
H	-4.69060000	-0.02190000	-2.42890000
C	-6.40150000	-1.18240000	0.37460000
H	-6.16290000	-1.74810000	1.27630000
H	-6.87280000	-0.24320000	0.68720000
H	-7.13850000	-1.75290000	-0.20590000
C	-3.30800000	5.40120000	0.59770000
H	-2.70730000	6.14230000	1.13650000
H	-4.19600000	5.90910000	0.20210000
H	-3.64790000	4.65440000	1.32430000
C	-3.36300000	3.74590000	-1.31380000
H	-2.82490000	3.36490000	-2.18390000
H	-3.65800000	2.90040000	-0.68340000
H	-4.28010000	4.22830000	-1.67360000
C	6.31770000	0.96660000	0.32730000
H	6.11170000	1.53150000	1.23760000

H	6.75170000	0.00520000	0.62540000
H	7.07260000	1.51220000	-0.25390000
C	5.40530000	0.01470000	-1.82140000
H	6.18210000	0.55260000	-2.37810000
H	5.78310000	-0.99340000	-1.61130000
H	4.53240000	-0.08770000	-2.47250000
